# Adiposity, metabolites, and colorectal cancer risk: Mendelian randomization study

**DOI:** 10.1186/s12916-020-01855-9

**Published:** 2020-12-17

**Authors:** Caroline J. Bull, Joshua A. Bell, Neil Murphy, Eleanor Sanderson, George Davey Smith, Nicholas J. Timpson, Barbara L. Banbury, Demetrius Albanes, Sonja I. Berndt, Stéphane Bézieau, D. Timothy Bishop, Hermann Brenner, Daniel D. Buchanan, Andrea Burnett-Hartman, Graham Casey, Sergi Castellví-Bel, Andrew T. Chan, Jenny Chang-Claude, Amanda J. Cross, Albert de la Chapelle, Jane C. Figueiredo, Steven J. Gallinger, Susan M. Gapstur, Graham G. Giles, Stephen B. Gruber, Andrea Gsur, Jochen Hampe, Heather Hampel, Tabitha A. Harrison, Michael Hoffmeister, Li Hsu, Wen-Yi Huang, Jeroen R. Huyghe, Mark A. Jenkins, Corinne E. Joshu, Temitope O. Keku, Tilman Kühn, Sun-Seog Kweon, Loic Le Marchand, Christopher I. Li, Li Li, Annika Lindblom, Vicente Martín, Anne M. May, Roger L. Milne, Victor Moreno, Polly A. Newcomb, Kenneth Offit, Shuji Ogino, Amanda I. Phipps, Elizabeth A. Platz, John D. Potter, Conghui Qu, J. Ramón Quirós, Gad Rennert, Elio Riboli, Lori C. Sakoda, Clemens Schafmayer, Robert E. Schoen, Martha L. Slattery, Catherine M. Tangen, Kostas K. Tsilidis, Cornelia M. Ulrich, Fränzel J. B. van Duijnhoven, Bethany van Guelpen, Kala Visvanathan, Pavel Vodicka, Ludmila Vodickova, Hansong Wang, Emily White, Alicja Wolk, Michael O. Woods, Anna H. Wu, Peter T. Campbell, Wei Zheng, Ulrike Peters, Emma E. Vincent, Marc J. Gunter

**Affiliations:** 1 MRC Integrative Epidemiology Unit at the University of Bristol, Oakfield House, Bristol, UK; 2 Population Health Sciences, Bristol Medical School, University of Bristol, Bristol, UK; 3 School of Cellular and Molecular Medicine, University of Bristol, Bristol, UK; 4 Nutrition and Metabolism Section, International Agency for Research on Cancer, World Health Organization, Lyon, France; 5 Public Health Sciences Division, Fred Hutchinson Cancer Research Center, Seattle, WA USA; 6 Division of Cancer Epidemiology and Genetics, National Cancer Institute, National Institutes of Health, Bethesda, MD USA; 7 Service de Génétique Médicale, Centre Hospitalier Universitaire (CHU) Nantes, Nantes, France; 8 Leeds Institute of Cancer and Pathology, University of Leeds, Leeds, UK; 9 Division of Clinical Epidemiology and Aging Research, German Cancer Research Center (DKFZ), Heidelberg, Germany; 10 Division of Preventive Oncology, German Cancer Research Center (DKFZ) and National Center for Tumor Diseases (NCT), Heidelberg, Germany; 11 German Cancer Consortium (DKTK), German Cancer Research Center (DKFZ), Heidelberg, Germany; 12 Colorectal Oncogenomics Group, Department of Clinical Pathology, The University of Melbourne, Parkville, Victoria Australia; 13 Victorian Comprehensive Cancer Centre, University of Melbourne Centre for Cancer Research, Parkville, Victoria Australia; 14 Genomic Medicine and Family Cancer Clinic, The Royal Melbourne Hospital, Parkville, Victoria Australia; 15 Institute for Health Research, Kaiser Permanente Colorado, Denver, CO USA; 16 Center for Public Health Genomics, University of Virginia, Charlottesville, VA USA; 17 Gastroenterology Department, Hospital Clínic, Institut d’Investigacions Biomèdiques August Pi i Sunyer (IDIBAPS), Centro de Investigación Biomédica en Red de Enfermedades Hepáticas y Digestivas (CIBEREHD), University of Barcelona, Barcelona, Spain; 18 Division of Gastroenterology, Massachusetts General Hospital and Harvard Medical School, Boston, MA USA; 19 Channing Division of Network Medicine, Brigham and Women’s Hospital and Harvard Medical School, Boston, MA USA; 20 Clinical and Translational Epidemiology Unit, Massachusetts General Hospital and Harvard Medical School, Boston, MA USA; 21 Broad Institute of Harvard and MIT, Cambridge, MA USA; 22 Division of Cancer Epidemiology, German Cancer Research Center (DKFZ), Heidelberg, Germany; 23 University Cancer Centre Hamburg (UCCH), University Medical Centre Hamburg-Eppendorf, Hamburg, Germany; 24 Department of Epidemiology and Biostatistics, Imperial College London, Norfolk Place, London, UK; 25 Department of Cancer Biology and Genetics and the Comprehensive Cancer Center, The Ohio State University, Columbus, OH USA; 26 Department of Medicine, Samuel Oschin Comprehensive Cancer Institute, Cedars-Sinai Medical Center, Los Angeles, CA USA; 27 Department of Preventive Medicine, Keck School of Medicine, University of Southern California, Los Angeles, CA USA; 28 Lunenfeld Tanenbaum Research Institute, Mount Sinai Hospital, University of Toronto, Toronto, Ontario Canada; 29 Epidemiology Research Program, American Cancer Society, Atlanta, GA USA; 30 Cancer Epidemiology Division, Cancer Council Victoria, Melbourne, Victoria Australia; 31 Centre for Epidemiology and Biostatistics, Melbourne School of Population and Global Health, The University of Melbourne, Melbourne, Victoria Australia; 32 Precision Medicine, School of Clinical Sciences at Monash Health, Monash University, Clayton, Victoria Australia; 33 Department of Preventive Medicine & USC Norris Comprehensive Cancer Center, Keck School of Medicine, University of Southern California, Los Angeles, CA USA; 34 Institute of Cancer Research, Department of Medicine I, Medical University Vienna, Vienna, Austria; 35 Department of Medicine I, University Hospital Dresden, Technische Universität Dresden (TU Dresden), Dresden, Germany; 36 Division of Human Genetics, Department of Internal Medicine, The Ohio State University Comprehensive Cancer Center, Columbus, OH USA; 37 Department of Biostatistics, University of Washington, Seattle, WA USA; 38 Department of Epidemiology, Johns Hopkins Bloomberg School of Public Health, Baltimore, MD USA; 39 Center for Gastrointestinal Biology and Disease, University of North Carolina, Chapel Hill, NC USA; 40 Department of Preventive Medicine, Chonnam National University Medical School, Gwangju, South Korea; 41 Jeonnam Regional Cancer Center, Chonnam National University Hwasun Hospital, Hwasun, South Korea; 42 University of Hawaii Cancer Center, Honolulu, HI USA; 43 Department of Family Medicine, University of Virginia, Charlottesville, VA USA; 44 Department of Clinical Genetics, Karolinska University Hospital, Stockholm, Sweden; 45 Department of Molecular Medicine and Surgery, Karolinska Institutet, Stockholm, Sweden; 46 CIBER Epidemiología y Salud Pública (CIBERESP), Madrid, Spain; 47 Biomedicine Institute (IBIOMED), University of León, León, Spain; 48 Julius Center for Health Sciences and Primary Care, University Medical Center Utrecht, Utrecht University, Utrecht, The Netherlands; 49 Oncology Data Analytics Program, Catalan Institute of Oncology-IDIBELL, L’Hospitalet de Llobregat, Barcelona, Spain; 50 Department of Clinical Sciences, Faculty of Medicine, University of Barcelona, Barcelona, Spain; 51 ONCOBEL Program, Bellvitge Biomedical Research Institute (IDIBELL), L’Hospitalet de Llobregat, Barcelona, Spain; 52 School of Public Health, University of Washington, Seattle, WA USA; 53 Clinical Genetics Service, Department of Medicine, Memorial Sloan-Kettering Cancer Center, New York, NY USA; 54 Department of Medicine, Weill Cornell Medical College, New York, NY USA; 55 Program in MPE Molecular Pathological Epidemiology, Department of Pathology, Brigham and Women’s Hospital, Harvard Medical School, Boston, MA USA; 56 Department of Epidemiology, Harvard T.H. Chan School of Public Health, Boston, MA USA; 57 Cancer Immunology and Cancer Epidemiology Programs, Dana-Farber Harvard Cancer Center, Boston, MA USA; 58 Broad Institute of MIT and Harvard, Cambridge, MA USA; 59 Department of Epidemiology, University of Washington, Seattle, WA USA; 60 University of Washington, Seattle, WA USA; 61 Centre for Public Health Research, Massey University, Wellington, New Zealand; 62 Health Sciences Centre, University of Canterbury, Christchurch, New Zealand; 63 Public Health Directorate, Asturias, Spain; 64 Department of Community Medicine and Epidemiology, Lady Davis Carmel Medical Center, Haifa, Israel; 65 Ruth and Bruce Rappaport Faculty of Medicine, Technion-Israel Institute of Technology, Haifa, Israel; 66 Clalit National Cancer Control Center, Haifa, Israel; 67 Department of Epidemiology and Biostatistics, School of Public Health, Imperial College London, London, UK; 68 Division of Research, Kaiser Permanente Northern California, Oakland, CA USA; 69 Department of General Surgery, University Hospital Rostock, Rostock, Germany; 70 Department of Medicine and Epidemiology, University of Pittsburgh Medical Center, Pittsburgh, PA USA; 71 Department of Internal Medicine, University of Utah, Salt Lake City, UT USA; 72 SWOG Statistical Center, Fred Hutchinson Cancer Research Center, Seattle, WA USA; 73 Department of Hygiene and Epidemiology, University of Ioannina School of Medicine, Ioannina, Greece; 74 Huntsman Cancer Institute and Department of Population Health Sciences, University of Utah, Salt Lake City, UT USA; 75 Division of Human Nutrition and Health, Wageningen University & Research, Wageningen, The Netherlands; 76 Department of Radiation Sciences, Oncology Unit, Umeå University, Umeå, Sweden; 77 Wallenberg Centre for Molecular Medicine, Umeå University, Umeå, Sweden; 78 Department of Molecular Biology of Cancer, Institute of Experimental Medicine of the Czech Academy of Sciences, Prague, Czech Republic; 79 Institute of Biology and Medical Genetics, First Faculty of Medicine, Charles University, Prague, Czech Republic; 80 Faculty of Medicine and Biomedical Center in Pilsen, Charles University, Pilsen, Czech Republic; 81 Department of Epidemiology, University of Washington School of Public Health, Seattle, WA USA; 82 Institute of Environmental Medicine, Karolinska Institutet, Stockholm, Sweden; 83 Discipline of Genetics, Memorial University of Newfoundland, St John’s, Canada; 84 University of Southern California, Preventative Medicine, CA, Los Angeles USA; 85 Behavioral and Epidemiology Research Group, American Cancer Society, Atlanta, GA USA; 86 Division of Epidemiology, Department of Medicine, Vanderbilt-Ingram Cancer Center, Vanderbilt Epidemiology Center, Vanderbilt University School of Medicine, Nashville, TN USA

**Keywords:** Body mass index, Waist-to-hip ratio, Colorectal cancer, Mendelian randomization, Metabolism, NMR, Epidemiology, GECCO, CORECT, CCFR

## Abstract

**Background:**

Higher adiposity increases the risk of colorectal cancer (CRC), but whether this relationship varies by anatomical sub-site or by sex is unclear. Further, the metabolic alterations mediating the effects of adiposity on CRC are not fully understood.

**Methods:**

We examined sex- and site-specific associations of adiposity with CRC risk and whether adiposity-associated metabolites explain the associations of adiposity with CRC. Genetic variants from genome-wide association studies of body mass index (BMI) and waist-to-hip ratio (WHR, unadjusted for BMI; *N* = 806,810), and 123 metabolites from targeted nuclear magnetic resonance metabolomics (*N* = 24,925), were used as instruments. Sex-combined and sex-specific Mendelian randomization (MR) was conducted for BMI and WHR with CRC risk (58,221 cases and 67,694 controls in the Genetics and Epidemiology of Colorectal Cancer Consortium, Colorectal Cancer Transdisciplinary Study, and Colon Cancer Family Registry). Sex-combined MR was conducted for BMI and WHR with metabolites, for metabolites with CRC, and for BMI and WHR with CRC adjusted for metabolite classes in multivariable models.

**Results:**

In sex-specific MR analyses, higher BMI (per 4.2 kg/m^2^) was associated with 1.23 (95% confidence interval (CI) = 1.08, 1.38) times higher CRC odds among men (inverse-variance-weighted (IVW) model); among women, higher BMI (per 5.2 kg/m^2^) was associated with 1.09 (95% CI = 0.97, 1.22) times higher CRC odds. WHR (per 0.07 higher) was more strongly associated with CRC risk among women (IVW OR = 1.25, 95% CI = 1.08, 1.43) than men (IVW OR = 1.05, 95% CI = 0.81, 1.36). BMI or WHR was associated with 104/123 metabolites at false discovery rate-corrected *P* ≤ 0.05; several metabolites were associated with CRC, but not in directions that were consistent with the mediation of positive adiposity-CRC relations. In multivariable MR analyses, associations of BMI and WHR with CRC were not attenuated following adjustment for representative metabolite classes, e.g., the univariable IVW OR for BMI with CRC was 1.12 (95% CI = 1.00, 1.26), and this became 1.11 (95% CI = 0.99, 1.26) when adjusting for cholesterol in low-density lipoprotein particles.

**Conclusions:**

Our results suggest that higher BMI more greatly raises CRC risk among men, whereas higher WHR more greatly raises CRC risk among women. Adiposity was associated with numerous metabolic alterations, but none of these explained associations between adiposity and CRC. More detailed metabolomic measures are likely needed to clarify the mechanistic pathways.

**Supplementary information:**

The online version contains supplementary material available at 10.1186/s12916-020-01855-9.

## Background

Colorectal cancer (CRC) is one of the most commonly diagnosed cancers among adults globally [1–3]. Obesity is viewed as a likely cause of CRC by the International Agency for Research on Cancer (IARC), the American Institute for Cancer Research (AICR), and the World Cancer Research Fund (WCRF) [3, 4], based largely on positive associations between adiposity and CRC risk from observational epidemiology. Further, the limited data available from observational studies suggest that intentional weight loss lowers the risk of CRC in postmenopausal women [5]. Mendelian randomization (MR) studies, which use genetic variants as instruments (proxies) for adiposity given their randomly allocated and fixed nature [6], further support causality [7–9]. Despite this growing consensus, it remains unclear whether the effect of adiposity on CRC risk differs among men and women, whether the relationship varies by CRC sub-site, and what the underlying biological mechanisms are. These are important to clarify given the ongoing obesity epidemic and difficulties in reducing adiposity itself [10, 11].

Observationally, body mass index (BMI) relates more strongly to CRC risk among men and waist-to-hip ratio (WHR) relates similarly to CRC risk among men and women [12]. However, recent MR studies suggest that higher BMI more greatly raises CRC risk among women, while higher WHR more greatly raises CRC risk among men [7, 8]. Whether these MR estimates are robust is unclear because they were based on relatively small sample sizes, genetic instruments that were not sex-specific, and genetic instruments for WHR that were conditioned on BMI—all potential sources of bias [13–17].

Adiposity alters the systemic metabolism [18–20], but evidence for the effects of adiposity-altered metabolites on CRC is scarce. One MR study suggested that total cholesterol raises CRC risk [21], while others suggested no effect of blood glucose [22] and mixed support for fatty acids [23]. Overall, the scope of metabolic traits examined has also been narrow. Targeted metabolomics allows deeper phenotyping at a large scale [24], and its recent integration with genotype data [25] enables us to examine the associations of metabolites with CRC using MR. Expanded genotype data for CRC is also available [26], affording a sample size six times larger than used in previous MR studies (58,221 cases, 67,694 controls).

This study has two aims. First, we aimed to better estimate sex-specific effects of adiposity on CRC risk using two-sample MR. We examined associations of BMI and WHR with CRC risk using expanded GWAS data and genetic instruments for exposures that were sex-specific and were not mutually conditioned, to reduce bias [13–17]. Second, we aimed to identify potential metabolic mediators of effects of adiposity on CRC risk using two-step MR (by examining associations of BMI and WHR with metabolites, and of BMI- or WHR-related metabolites with CRC risk) and multivariable MR (by adjusting associations of BMI and WHR with CRC for representative metabolites).

## Methods

### Study design

**Fig. 1:**
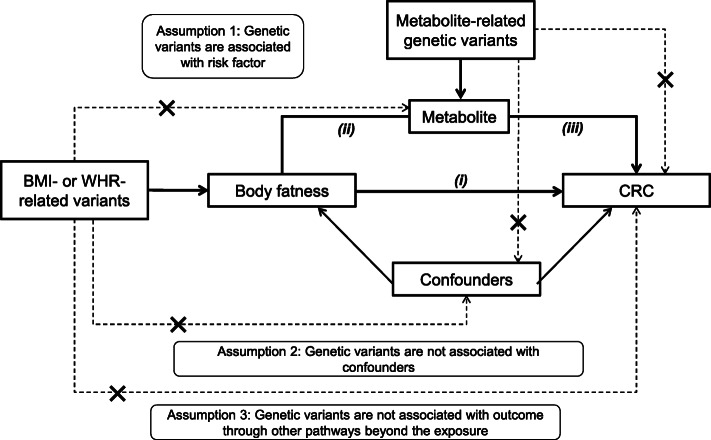
Study aims and assumptions. Study aims are to (1) estimate the total effect of adiposity on CRC risk using genetic instruments for BMI and WHR ((i) unadjusted for BMI) and (2) estimate the mediated effect of adiposity on CRC risk by metabolites from targeted NMR metabolomics. Aim 2 is addressed using two approaches: (1) two-step MR wherein effects are examined of adiposity on metabolites (ii) and of adiposity-related metabolites on CRC risk (iii) and (2) multivariable MR wherein effects of adiposity on CRC (i) are examined with adjustment for the effect of representative metabolite classes on CRC (iii). Sex-specific analyses were performed when sex-specific GWAS estimates for exposure and outcome were both available. When ≥ 2 SNP instruments were available, up to 4 MR models were applied: the inverse-variance-weighted (IVW) model which assumes that none of the SNPs are pleiotropic [28], the weighted median (WM) model which allows up to half of the included SNPs to be pleiotropic and is less influenced by outliers [28], the weighted mode model which assumes that the most common effect is consistent with the true causal effect [29], and the MR-Egger model which provides an estimate of association magnitude allowing all SNPs to be pleiotropic [30]. Analyses with metabolites as outcomes were conducted within discovery aims wherein *P* value thresholds are applied to prioritize traits with the strongest evidence of association to be taken forward into further stages of analysis (with CRC risk). Analyses with CRC as outcomes were conducted within estimation aims wherein *P* values are interpreted as continuous indicators of evidence strength and focus is on effect size and precision [31, 32]

We used two-sample MR to examine the associations (pertaining to estimates of the effect predicted from genetic variants used as instruments) of adiposity with CRC risk, of adiposity with metabolites, of adiposity-associated metabolites with CRC risk, and finally of adiposity with CRC risk adjusted for representative metabolites. In two-sample MR, SNP-exposure and SNP-outcome associations are obtained from different study sources and combined as a ratio to estimate the effects of exposures on outcomes [13, 27]. Our study aims and assumptions are shown in Fig. 1.

### Adiposity instruments

We identified SNPs that were independently associated (low linkage disequilibrium (LD), *R*^2^ < 0.001) with BMI and WHR (unadjusted for BMI) at *P* < 5 × 10^−8^ from a recent large-scale genome-wide association study (GWAS) meta-analysis of 221,863 to 806,810 male and female adults of European ancestry from the Genetic Investigation of ANthropometric Traits (GIANT) consortium and the UK Biobank [33] (Additional file 1: Table S1). BMI and WHR are expressed in standard deviation (SD) units. For sex-combined analyses of BMI and WHR, 312 and 209 SNPs were used, respectively. For sex-specific analyses of BMI, 185 and 152 SNPs were used for women and men, respectively. For sex-specific analyses of WHR, 153 and 64 SNPs were used for women and men, respectively. The proportion of variance explained in adiposity traits by instruments ranged from 0.3 to 5.04% (these were based on approximations for BMI using the equation described by Shim et al. [34]), and *F*-statistics (a formal test of whether variance explained is sufficiently high to avoid weak instrument bias) for adiposity instruments ranged from 75.81 to 124.49 (Additional file 1: Table S2) which indicated instrument strength above the recommended minimum levels [35].

### Metabolite instruments

We identified SNPs that were independently associated (*R*^2^ < 0.001 and *P* < 5 × 10^−8^) with metabolites from a GWAS of 123 traits from targeted nuclear magnetic resonance (NMR) metabolomics (Additional file 1: Table S1); these included lipoprotein subclass-specific lipids, amino acids, fatty acids, inflammatory glycoproteins, and others [25]. Between 13,476 and 24,925 adults (men and women combined) of European ancestry were included. Metabolic traits are expressed in SD units. The proportion of variance explained in metabolites by instruments ranged from 0.44 to 12.49%, and *F*-statistics for metabolite instruments ranged from 30.2 to 220.8 (Additional file 1: Table S2) which indicated sufficient instrument strength for univariable analyses.

### Colorectal cancer GWAS data

We obtained SNP estimates from the most comprehensive GWAS of CRC to date [26], including 58,221 cases and 67,694 controls (sexes combined) from 45 studies within 3 consortia: Genetics and Epidemiology of Colorectal Cancer Consortium (GECCO), Colorectal Cancer Transdisciplinary Study (CORECT), and Colon Cancer Family Registry (CCFR). Across these studies, there were 28,207 CRC cases and 22,204 controls among men, and 24,568 CRC cases and 23,736 controls among women. Cases were diagnosed by a physician and recorded overall and by site (colon, proximal colon, distal colon, rectum). Approximately 92% of the participants were White-European (~ 8% were East Asian). Case distributions are outlined in Additional file 1: Table S3; other study characteristics are detailed elsewhere [26]. Ethics were approved by respective institutional review boards.

### Statistical approach

First, we examined the associations of BMI and WHR with overall and site-specific CRC using SNP estimates from sex-combined GWAS of exposures as well as outcomes. We then examined the associations of BMI and WHR with overall CRC based on SNP estimates from sex-specific GWAS of exposure as well as outcome (sex-specific GWAS were not available and thus not used for site-specific CRC). Summary statistics were harmonized using the harmonise_data function within the TwoSampleMR R package [36]. All GWAS were assumed to be coded on the forward strand, and harmonization was confirmed as consistent using option 2 of the “action” argument. As sensitivity analyses, up to four MR methods were used to generate effect estimates using the TwoSampleMR R package [36] which make differing pleiotropy assumptions (detailed in Fig. 1 legend) [29, 36, 37]. When only a single SNP was available, the Wald ratio was used [38]. When ≥ 2 SNPs were available, random-effects inverse-variance-weighted (IVW) [36], MR-Egger [30], weighted median (WM) [28], and weighted mode [29] models were used. Cochrane’s *Q*-statistic was used to assess the heterogeneity of SNP effects (smaller *P* values indicating higher heterogeneity and higher potential for directional pleiotropy [39]). Scatter plots were used to compare MR models, and “leave-one-SNP-out” analyses were used to detect SNP outliers [40].

Second, we examined associations of BMI and WHR with metabolites using results from sex-combined GWAS for exposures as well as outcomes (sex-specific GWAS were not available for metabolites, and so sex-specific analyses were not conducted) and the MR models described above. Each metabolite (analyzed as an outcome) that was associated with either BMI or WHR based on an IVW model *P* value ≤ 0.05 following a false discovery rate (FDR) correction (Benjamini-Hochberg method [41]) was taken forward and examined for association with CRC risk using the IVW model (if ≥ 2 SNPs) or the Wald ratio (if 1 SNP). Multivariable MR [42] was also used to examine the associations of BMI and WHR with CRC risk, adjusting for single metabolites that were representative of various metabolite classes based on previous network analyses [43] and that had the highest instrument strength based on the *F*-statistic (Additional file 1: Table S2). As a positive control, we adjusted BMI for WHR as a covariate (which is expected to attenuate the association of BMI with CRC risk), and likewise, we adjusted WHR for BMI as a covariate with the same expectation. A smaller set of SNPs for BMI and WHR based on earlier GWAS [44, 45] was used for these multivariable models to avoid a relative dilution of metabolite instrument strength given that the number of SNPs for BMI and WHR from expanded GWAS far outnumbered those for metabolites. Conditional *F*-statistics were calculated for exposures in multivariable models [46].

In each instance, MR estimates are interpreted as the change in outcome per SD unit change in the exposure. Estimates for metabolite outcomes reflect SD unit change, and estimates for CRC outcomes reflect odds ratios (OR). Statistical analyses were performed using R (version 3.5.2).

## Results

### Associations of BMI and WHR with CRC risk

**Fig. 2:**
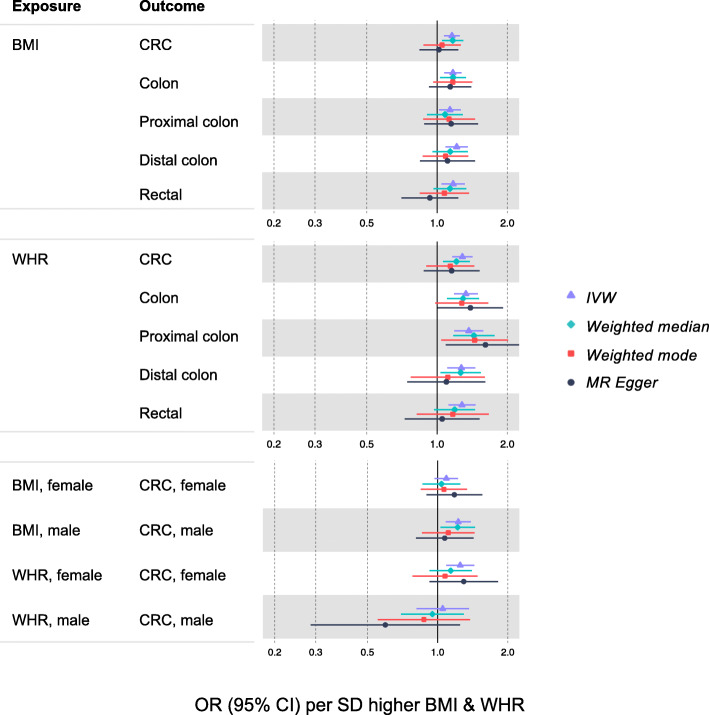
Associations of BMI and WHR with CRC risk based on two-sample MR. Sex-combined estimates are based on GWAS done among women and men together (for both exposure and outcome). Sex-specific estimates are based on GWAS done separately among women and men (for exposure as well as outcome)

In sex-combined analyses (Fig. 2; Additional file 1: Table S4), higher BMI (per 4.8 kg/m^2^) was associated with a higher risk of overall CRC (IVW OR = 1.16, 95% CI = 1.07, 1.26). The WM estimate was similar, but the MR-Egger and weighted mode estimates were both reduced (e.g., MR-Egger OR = 1.02, 95% CI = 0.84, 1.25). BMI associations were consistent across CRC sites. Associations were directionally consistent for WHR as for BMI but were marginally stronger—e.g., higher WHR (per 0.09 ratio) was associated with 1.28 (95% CI = 1.16, 1.42) times higher odds of CRC in an IVW model (MR-Egger and weighted mode estimates were each positive but of a smaller magnitude with wide intervals spanning the null). WHR associations were more consistent for colon rather than rectal sub-sites. SNP heterogeneity was similarly high for BMI and WHR (*P* value range across models = 9.54 × 10^−10^ to 1.97 × 10^−8^).

In sex-specific IVW models (Fig. 2; Additional file 1: Table S4), higher BMI (per 4.2 kg/m^2^) was associated with 1.23 (95% CI = 1.08, 1.38) times higher odds of CRC among men and 1.09 (95% CI = 0.97, 1.22) times higher odds of CRC (per 5.2 kg/m^2^) among women. In a WM model, this BMI estimate was robust among men (OR = 1.22, 95% CI = 1.02, 1.46) but reduced among women (OR = 1.04, 95% CI = 0.86, 1.26). MR-Egger and weighted mode estimates were similarly imprecise among men and women, and SNP heterogeneity was similar for both. In IVW models, higher WHR (per 0.07 ratio) was associated with 1.25 (95% CI = 1.08, 1.43) times higher odds of CRC among women; this estimate was 1.05 (95% CI = 0.81, 1.36) among men (per 0.07 ratio). This pattern was also supported by WM estimates (OR = 1.14, 95% CI = 0.91, 1.42 among women and OR = 0.95, 95% CI = 0.90, 1.29 among men), and by MR-Egger and weighted mode estimates. SNP heterogeneity was similarly high among men and women.

Scatter plots comparing different MR models and results of the “leave-one-SNP-out” analyses are presented in Additional file 2: Figures S1-42.

### Associations of BMI and WHR with metabolites

In sex-combined analyses, higher BMI (per 4.8 kg/m^2^) or WHR (per 0.09 ratio) was associated with 104 metabolites based on FDR-corrected *P* value ≤ 0.05 in IVW models (Additional file 2: Figures S43-47; Additional file 1: Table S5). Evidence was strong in relation to lipids including total cholesterol and triglycerides in very low-density lipoproteins (VLDL), low-density lipoproteins (LDL), and high-density lipoproteins (HDL)—e.g., 0.23 SD (95% CI = 0.15, 0.31) higher triglycerides in large VLDL from higher BMI. Associations of higher BMI were also strong with lactate, pyruvate, and branched-chain amino acids—e.g., 0.19 SD (95% CI = 0.13, 0.25) higher isoleucine—and with inflammatory glycoproteins (0.28 SD, 95% CI = 0.20, 0.36 higher). Similar patterns were seen for WHR.

### Associations of BMI- or WHR-related metabolites with CRC

**Fig. 3:**
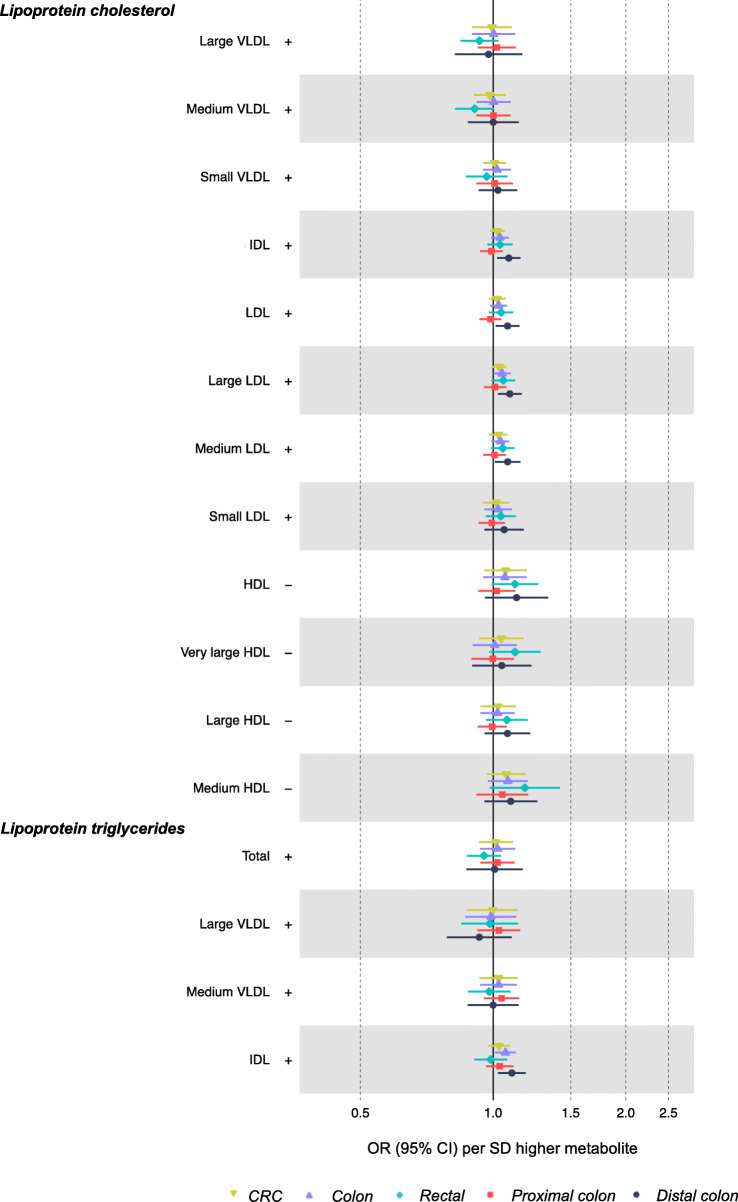
Associations of BMI- or WHR-related lipid metabolites with CRC risk based on two-sample MR (IVW method). Estimates reflect the OR (95% CI) for CRC per SD higher metabolite that is associated (as an outcome) with BMI or WHR. +/− symbols indicate the direction of association of BMI or WHR with that metabolite

**Fig. 4:**
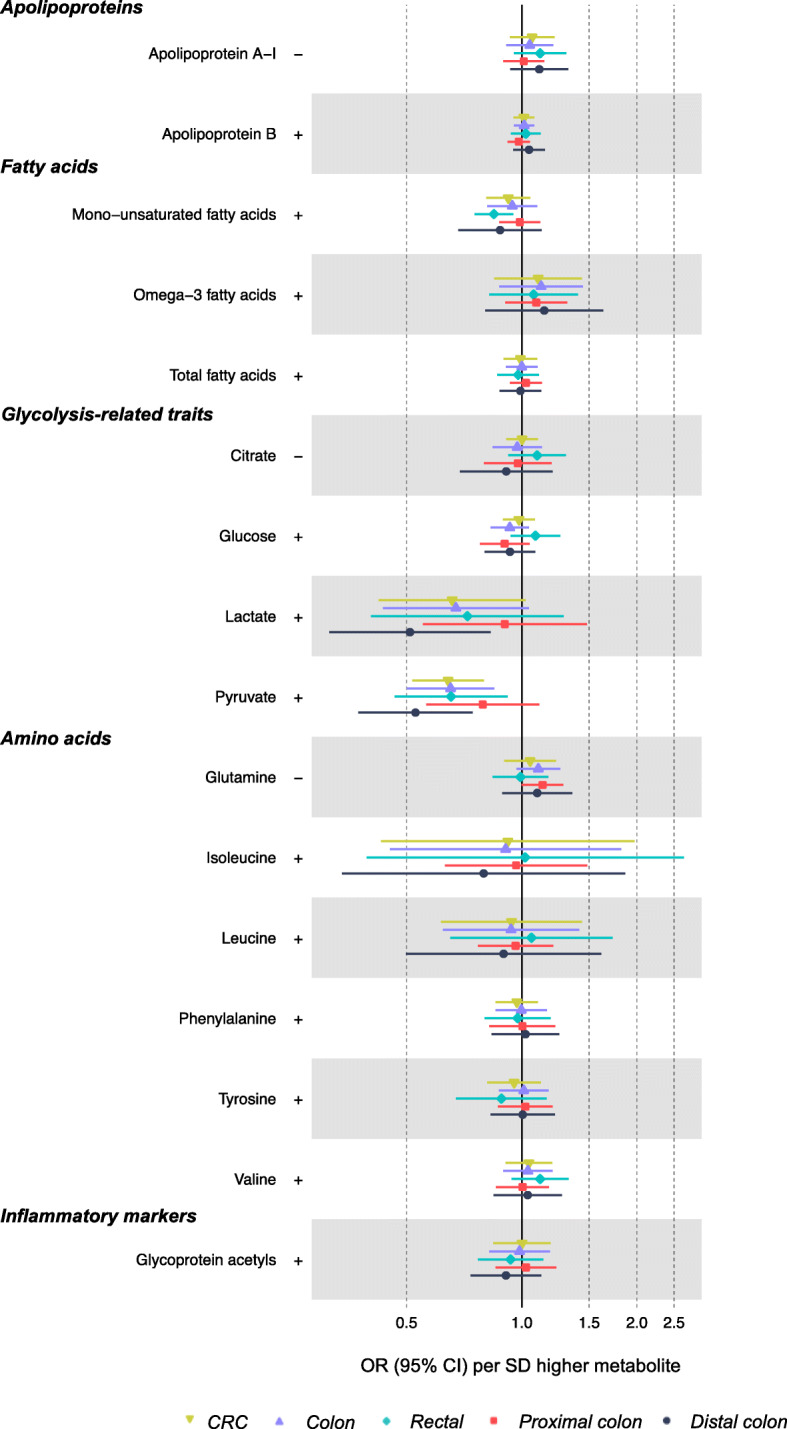
Associations of BMI- or WHR-related non-lipid metabolites with CRC risk based on two-sample MR (IVW method). Estimates reflect the OR (95% CI) for CRC per SD higher metabolite that is associated (as an outcome) with BMI or WHR. +/− symbols indicate the direction of association of BMI or WHR with that metabolite

Of 104 metabolites associated (as outcomes) with BMI or WHR in sex-combined analyses, 100 had SNPs for use in Wald or IVW models. As shown in Additional file 1: Table S1, 321 unique SNPs were used to instrument 100 metabolites (3 metabolites had 1 SNP, 13 metabolites had < 5 SNPs, and 51 metabolites had < 10 SNPs; SNP counts across metabolites ranged from 1 to 26). Lipid traits showed generally weak associations with CRC which were also in directions inconsistent with the mediation of the adiposity-CRC relationship—e.g., lipids in medium HDL were positively associated with CRC, but these had been negatively associated with BMI or WHR (Fig. 3; Additional file 1: Table S6). In contrast, there was more consistent evidence of a positive association of lipids in intermediate-density lipoprotein (IDL), VLDL, and LDL with a risk of distal colon cancer, and these lipids had been positively associated with higher BMI or WHR. For example, higher total lipids in IDL (per SD) were associated with 1.09 (95% CI = 1.02, 1.15) times higher odds of distal colon cancer. Lipids were unassociated with the risk of proximal colon cancer. Fatty acids were unassociated with CRC risk except for higher monounsaturated fatty acid levels which were associated with a lower risk of rectal cancer (IVW OR = 0.85, 95% CI = 0.75, 0.95; Fig. 4). Lactate and pyruvate were inversely associated with CRC at 0.66 (95% CI = 0.42, 1.03) times lower odds and 0.64 (95% CI = 0.52, 0.80) times lower odds, respectively. However, these metabolites were positively associated with BMI, and so directions were inconsistent with the mediation of the adiposity-CRC relationship. Amino acids and glycoprotein acetyls were unassociated with CRC risk.

### Associations of BMI and WHR with CRC risk independent of metabolites

**Fig. 5:**
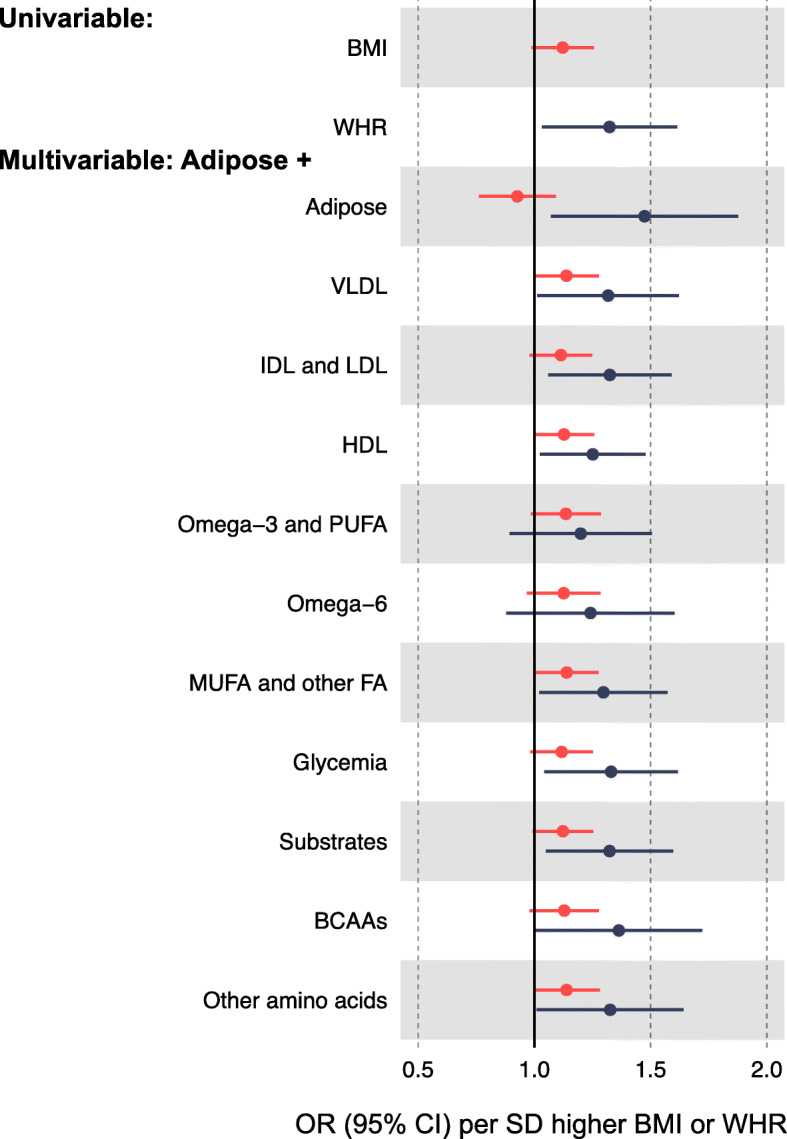
Associations of BMI and WHR with CRC risk independent of various metabolite classes based on multivariable MR. Metabolite classes are based on a single representative metabolite from a previous network analysis [43], as follows: VLDL (triglycerides in small VLDL); IDL and LDL (total cholesterol in medium LDL), HDL (triglycerides in very large HDL), Omega-3 and PUFA (other polyunsaturated fatty acids than 18:2), Omega-6 (18:2, linoleic acid), MUFA and other fatty acids (Omega-9 and saturated fatty acids), glycemia (glucose), substrates (citrate), branched-chain amino acids (leucine), and other amino acids (glutamine). Adipose adjustments include the alternative adiposity trait (WHR or BMI) as a positive control

The association of BMI with overall CRC was not attenuated following adjustment for various metabolite classes (Fig. 5; Additional file 1: Table S7). The univariable IVW OR for BMI (per 4.77 kg/m^2^ higher, based on 67 SNPs) in relation to CRC was 1.12 (95% CI = 1.00, 1.26), whereas this IVW OR was 1.14 (95% CI = 1.01, 1.29) adjusting for VLDL lipids and 1.11 (95% CI = 0.99, 1.26) adjusting for IDL and LDL lipids. Attenuation was greater when adjusting the BMI-CRC association for WHR (positive control), at IVW OR = 0.93 (95% CI = 0.78, 1.11). Results for WHR in relation to CRC were directionally consistent as seen for BMI, with a lack of attenuation upon adjustment for metabolite classes.

## Discussion

We aimed to better estimate sex-specific effects of adiposity on CRC risk and to identify potential metabolic mediators of the effects of adiposity on CRC, using two-sample MR methods and expanded sample sizes. Our results, based on genetic instruments for adiposity that were sex-specific and were not mutually conditioned, suggest that higher BMI more greatly raises CRC risk among men, whereas higher WHR more greatly raises CRC risk among women. In sex-combined mediation analyses, adiposity was associated with numerous metabolic alterations, but none of these alterations explained the associations between adiposity and CRC. More detailed metabolomic measures are likely needed to clarify the mechanistic pathways.

Observational [3, 47] and MR [7–9] studies have suggested adverse effects of adiposity on CRC risk, but causal evidence has been lacking regarding sex specificity. Previous MR studies suggested stronger effects of BMI on CRC risk among women [7–9], which contradicts observational suggestions of stronger effects among men [12]. The genetic regulation of BMI and WHR shows strong sexual dimorphism, thought attributable to the influence of sex hormones, namely estrogen, and it is important to capture these differences in MR estimates [48, 49]. Our new results are based on instruments for BMI and WHR that were sex-specific and a sample size for CRC that was six times larger than used previously which enabled higher power relative to two previous MR studies of BMI, WHR, and CRC risk [7, 8] (Additional file 2: Figure S48). These new results suggest that BMI more greatly raises CRC risk among men—a reversal of previous MR estimates. This new pattern for BMI and CRC (22% higher risk among men per 4.2 kg/m^2^ and 9% higher risk among women per 5.2 kg/m^2^) is highly consistent with observational estimates reviewed by IARC (22% higher risk in men and 9% higher risk in women per 5 kg/m^2^ [4]). Our results also support a reversal of previous MR estimates for WHR, with risk now appearing higher among women than among men. This is unexpected since BMI and abdominal fat measures correlate highly [50, 51]; however, given that fat storage is more peripheral in women [18, 19], WHR (unadjusted for BMI) may be a better proxy for the extremeness of fat volume among women since fat may be stored more abdominally only when peripheral fat stores are overwhelmed. As a post hoc comparison, we repeated analyses of the main effects of adiposity on CRC using the sex-combined adiposity instruments in relation to split samples of men and women (Additional file 1: Table S8, A) to examine the potential for biased results. These suggest that use of sex-combined instruments for BMI and WHR would lead to the conclusion that both are associated with higher CRC risk in males as well as females, but with still higher risk with BMI among males and with WHR among females, in contrast to previous MR studies [7, 8]. This suggests that discrepancies in the result patterns are most likely due to the differences in the power of the main adiposity-CRC relationship (Additional file 2: Figure S48).

SNP heterogeneity was high for BMI and WHR with CRC, although this was similar between sexes and directions of effect from sensitivity models were consistent, suggesting balanced SNP heterogeneity. One cause of heterogeneity may be pleiotropy in violation of the exclusion restriction criteria (assumption 3, Fig. 1). This is not unexpected due to the large number of SNPs included in the adipose trait instruments and the many underlying biological pathways that explain variation in adiposity. A future approach to minimizing heterogeneity in instrument selection could be to analyze the association between subsets of genetic variants related to specific pathways of BMI and WHR in relation to CRC; this requires more biological knowledge of these genetic variants than currently exists.

Given the difficulty of weight loss [11] and the ongoing obesity epidemic, it is increasingly important to identify the biological pathways which explain the effect of adiposity on the risk of chronic diseases including CRC [10]. Adipose tissue is highly metabolically active and secretes pro-inflammatory cytokines such as interleukin (IL)-6 and tumor necrosis factor (TNF)-alpha which may promote tumor initiation [52]. Adipose tissue-derived inflammation also promotes insulin resistance in glucose storage tissues that can lead to hyperinsulinemia [53], and insulin and insulin-like growth factors (IGF) such as IGF-1 have pro-mitogenic and anti-apoptotic effects that are cancer promotive [47, 54–58]. Our current results suggest effects of BMI or WHR on numerous lipids and pre-glycemic traits; however, few of these traits had any strong association with CRC risk, and the few that did were in a direction that was inconsistent with a mediating role in the adiposity-CRC relationship. Results of a series of multivariable MR models, which adjusted for various metabolites considered representative of broader metabolite classes [42], suggested that associations of BMI and WHR with CRC risk were highly independent of these metabolites. However, this analysis may be limited by weak instrument bias [59] given that *F*-statistics for metabolite instruments included in each multivariable MR model were relatively low. Nevertheless, the results of two complementary approaches to mediation (two-step MR and multivariable MR) provide little evidence that the effects of adiposity on CRC risk are mediated by adiposity-related metabolites that are detectable by NMR metabolomics. Future studies could examine metabolites, proteins, hormones, and inflammatory factors that are detectable by other metabolomic and proteomic platforms.

The few traits that did show consistent directions of effect included total lipids in IDL, LDL, and VLDL particles which were raised by BMI and which in turn raised the risk of distal colon cancer specifically (not proximal colon or rectal cancer). If robust, this pattern may reflect differential sensitivity of colon regions to lipid exposure owing to divergent functions (the distal colon functions primarily in the storage of resultant fecal matter whereas the proximal colon functions primarily in water absorption and fecal solidification [60]), or it may reflect differential detectability through screening (proximal colon tumors tend to be detected in older ages and at more advanced stages [60]). Colorectal anatomical regions may also have distinct molecular features [61], e.g., the distal colon may be more susceptible to p53 mutations and chromosomal instability [62], whereas the proximal colon may be more mucinous and susceptible to microsatellite instability and B-Raf proto-oncogene expression [63, 64]. Several meta-analyses of long-term follow-ups of randomized controlled trials of LDL cholesterol-lowering statin use suggested no strong evidence of a protective effect of statin used on CRC risk [65–67]; CRC sub-sites were largely unexamined. One previous MR study suggested an adverse effect of higher LDL cholesterol, and a protective effect of genetically proxied statin use, on overall CRC risk [21]; again, CRC sub-sites were not examined. Prospective observational evidence for LDL cholesterol and CRC risk is less consistent than for total cholesterol or triglycerides; heterogeneity in meta-analyzed effect estimates is much higher for LDL cholesterol (82.7% based on an *I*^2^ statistic) compared with total cholesterol and triglycerides (46.7% and 47.8%, respectively) [68]. Prospective estimates of lipoprotein subclass measures from metabolomic platforms are lacking as these are only recently available at scale.

The limitations of this study include the non-specificity of genetic variants used as instruments for some metabolites which stems from their expectedly correlated nature (e.g., rs1260326, a SNP in *GCKR*, was included in genetic instruments for 54 metabolites). A total of 321 unique SNPs was used to instrument 100 metabolites, but the number of instruments available for a given metabolite was typically small. This limits causal inference for individual traits but should not prevent the identification of relevant classes of traits (e.g., lipid, amino acid). It should also be stressed that genetic variants used for metabolites may alter the enzyme expression and so serve as instruments for the metabolizing enzyme itself, not factors influenced downstream of that enzyme. Since inference in MR applies to the most proximal trait that the genetic variant relates to [15], directing inference to specific glycolytic traits as distinct from their downstream consequences like insulin resistance [69] (a key result of higher fatness and trigger of tumorigenesis [61]) is difficult and requires stronger genetic instruments alongside mechanistic insights from preclinical studies [70]. Adiposity was measured indirectly using BMI and WHR because these correlate highly with more objectively measured fat indexes [50, 51] and allow much larger GWAS sample sizes than otherwise possible (comparably strong GWAS were unavailable for waist circumference). UK Biobank data are included within GWAS for both the exposure and outcome used for MR estimates of adiposity for CRC risk. Sample overlap in a two-sample MR setting is reported to contribute to weak instrument bias and inflated type one error rates, resulting in MR estimates that are biased towards confounding-prone observational estimates [71]. However, given that the proportion of sample overlap is presently low (< 5%) and estimated *F*-statistics are relatively high (each > 70 for adiposity traits), we do not expect considerable bias here. As a post hoc comparison, we obtained CRC summary GWAS statistics with UK Biobank excluded and repeated MR analyses of adiposity for CRC risk. Estimates were largely consistent with or without the inclusion of UK Biobank data (Additional file 1: Table S8, B). Our sex-specific MR investigations were confined to effects of adiposity on overall CRC because sex-specific GWAS were unavailable for site-specific CRC and metabolite outcomes. Sex-stratified GWAS of such outcomes would enable these in the future.

## Conclusions

Our results based on sex-specific MR instruments and expanded sample sizes suggest that higher BMI more greatly raises CRC risk among men, whereas higher WHR more greatly raises CRC risk among women. In sex-combined mediation analyses, adiposity was associated with numerous metabolic alterations, but none of these alterations explained the associations between adiposity and CRC. More detailed metabolomic measures are likely needed to clarify the mechanistic pathways.

## Supplementary Information

**Additional file 1: Table S1.** Genetic variants used to instrument BMI, WHR and metabolites. **Table S2.** Assesment of instrument strength. **Table S3.** Colorectal cancer case distributions by study, sex and site. **Table S4.** LogOR colorectal cancer per SD higher BMI or WHR. **Table S5.** Beta change in NMR-detected metabolite per SD higher BMI or WHR. **Table S6.** LogOR colorectal cancer per SD higher BMI or WHR-driven NMR-detected metabolite. **Table S7.** Risk of overall colorectal cancer per SD higher adipose or metabolite trait, estimated using multivariable Mendelian randomization. **Table S8.** Posthoc investigations.

**Additional file 2:** **Figure S1.** Scatter plot of SNP-BMI and SNP-CRC associations. **Figure S2.** Scatter plot of SNP-BMI and SNP-CRC associations (female specific). **Figure S3.** Scatter plot of SNP-BMI and SNP-CRC associations (male specific). **Figure S4.** Scatter plot of SNP-BMI and SNP-colon cancer associations. **Figure S5.** Scatter plot of SNP-BMI and SNP-proximal colon cancer associations. **Figure S6.** Scatter plot of SNP-BMI and SNP-distal colon cancer associations. **Figure S7.** Scatter plot of SNP-BMI and SNP-rectal cancer associations. **Figure S8.** Forest plot showing individual SNP (black) and combined MR estimates (red; Egger and IVW) for the effect of BMI on CRC. **Figure S9.** Forest plot showing individual SNP (black) and combined MR estimates (red; Egger and IVW) for the effect of BMI on CRC (female specific). **Figure S10.** Forest plot showing individual SNP (black) and combined MR estimates (red; Egger and IVW) for the effect of BMI on CRC (male specific). **Figure S11.** Forest plot showing individual SNP (black) and combined MR estimates (red; Egger and IVW) for the effect of BMI on colon cancer. **Figure S12.** Forest plot showing individual SNP (black) and combined MR estimates (red; Egger and IVW) for the effect of BMI on proximal colon cancer. **Figure S13.** Forest plot showing individual SNP (black) and combined MR estimates (red; Egger and IVW) for the effect of BMI on distal colon cancer. **Figure S14.** Forest plot showing individual SNP (black) and combined MR estimates (red; Egger and IVW) for the effect of BMI on rectal cancer. **Figure S15.** Leave-one-out plot showing the association between BMI and CRC, following SNP-by-SNP removal from the model. **Figure S16.** Leave-one-out plot showing the association between BMI and CRC (femalespecific), following SNP-by-SNP removal from the model. **Figure S17.** Leave-one-out plot showing the association between BMI and CRC (malespecific), following SNP-by-SNP removal from the model. **Figure S18.** Leave-one-out plot showing the association between BMI and colon cancer, following SNP-by-SNP removal from the model. **Figure S19.** Leave-one-out plot showing the association between BMI and proximal colon cancer, following SNP-by-SNP removal from the model. **Figure S20.** Leave-one-out plot showing the association between BMI and distal colon cancer, following SNP-by-SNP removal from the model. **Figure S21.** Leave-one-out plot showing the association between BMI and rectal cancer, following SNP-by-SNP removal from the model. **Figure S22.** Scatter plot of SNP-WHR and SNP-CRC associations. **Figure S23.** Scatter plot of SNP-WHR and SNP-CRC associations (female specific). **Figure S24.** Scatter plot of SNP-WHR and SNP-CRC associations (male specific). **Figure S25.** Scatter plot of SNP-WHR and SNP-colon cancer associations. **Figure S26.** Scatter plot of SNP-WHR and SNP-proximal colon cancer associations. **Figure S27.** Scatter plot of SNP-WHR and SNP-distal colon cancer associations. **Figure S28.** Scatter plot of SNP-WHR and SNP-rectal cancer associations. **Figure S29.** Forest plot showing individual SNP (black) and combined MR estimates (red; Egger and IVW) for the effect of WHR on CRC. **Figure S30.** Forest plot showing individual SNP (black) and combined MR estimates (red; Egger and IVW) for the effect of WHR on CRC (female specific). **Figure S31.** Forest plot showing individual SNP (black) and combined MR estimates (red; Egger and IVW) for the effect of WHR on CRC (male specific). **Figure S32.** Forest plot showing individual SNP (black) and combined MR estimates (red; Egger and IVW) for the effect of WHR on colon cancer. **Figure S33.** Forest plot showing individual SNP (black) and combined MR estimates (red; Egger and IVW) for the effect of WHR on proximal colon cancer. **Figure S34.** Forest plot showing individual SNP (black) and combined MR estimates (red; Egger and IVW) for the effect of WHR on distal colon cancer. **Figure S35.** Forest plot showing individual SNP (black) and combined MR estimates (red; Egger and IVW) for the effect of WHR on rectal cancer. **Figure S36.** Leave-one-out plot showing the association between WHR and CRC, following SNP-by-SNP removal from the model. **Figure S37.** Leave-one-out plot showing the association between WHR and CRC, following SNP-by-SNP removal from the model (female specific). **Figure S38.** Leave-one-out plot showing the association between WHR and CRC, following SNP-by-SNP removal from the model (male specific). **Figure S39.** Leave-one-out plot showing the association between WHR and colon cancer, following SNP-by-SNP removal from the model. **Figure S40.** Leave-one-out plot showing the association between WHR and proximal colon cancer, following SNP-by-SNP removal from the model. **Figure S41.** Leave-one-out plot showing the association between WHR and distal colon cancer, following SNP-by-SNP removal from the model. **Figure S42.** Leave-one-out plot showing the association between WHR and rectal cancer, following SNP-by-SNP removal from the model. **Figure S43.** Effects of BMI and WHR on circulating metabolite levels (NMR-detected metabolites, 1 of 5), based on two-sample MR (IVW models) in summary GWAS consortia data. **Figure S44.** Effects of BMI and WHR on circulating metabolite levels (NMR-detected metabolites, 2 of 5), based on two-sample MR (IVW models) in summary GWAS consortia data. **Figure S45.** Effects of BMI and WHR on circulating metabolite levels (NMR-detected metabolites, 3 of 5), based on two-sample MR (IVW models) in summary GWAS consortia data. **Figure S46.** Effects of BMI and WHR on circulating metabolite levels (NMR-detected metabolites, 4 of 5), based on two-sample MR (IVW models) in summary GWAS consortia data. **Figure S47.** Effects of BMI and WHR on circulating metabolite levels (NMR-detected metabolites, 5 of 5), based on two-sample MR (IVW models) in summary GWAS consortia data. **Figure S48.** Power curves for MR analyses, based on samples sizes for colorectal cancer in the present study (black), Thrift et al., 2015 (blue) and Jarvis et al., 2016 (purple). Upper and lower power curves describe genetic instruments explaining 5% and 0.3% of variance respectively for each study.

## Data Availability

The summary-level GWAS data on outcomes used in this study are available following an application to the Genetics and Epidemiology of Colorectal Cancer Consortium (GECCO): https://www.fredhutch.org/en/research/divisions/public-health-sciences-division/research/cancer-prevention/genetics-epidemiology-colorectal-cancer-consortium-gecco.html. A copy of the code used in this analysis is available at https://github.com/cb12104/adiposity_metabolites_crc.

## References

[1] Sung H, Siegel RL, Rosenberg PS, Jemal A. Emerging cancer trends among young adults in the USA: analysis of a population-based cancer registry. Lancet Public Health. 2019;4(3):E137-E147. 10.1016/S2468-2667(18)30267-6. 10.1016/S2468-2667(18)30267-6 30733056

[2] Mauri G, Sartore-Bianchi A, Russo AG, Marsoni S, Bardelli A, Siena S (2019). Early-onset colorectal cancer in young individuals. Mol Oncol.

[3] World Cancer Research Fund/American Institute for Cancer Research. Continuous Update Project Expert Report. Diet, nutrition, physical activity and colorectal cancer. 2018.

[4] Lauby-Secretan B, Scoccianti C, Loomis D, Grosse Y, Bianchini F, Straif K (2016). Body fatness and cancer—viewpoint of the IARC Working Group. N Engl J Med.

[5] Luo J, Hendryx M, Manson JE, Figueiredo JC, LeBlanc ES, Barrington W (2019). Intentional weight loss and obesity-related cancer risk. JNCI Cancer Spectrum.

[6] Davey Smith G, Ebrahim S (2003). ‘Mendelian randomization’: can genetic epidemiology contribute to understanding environmental determinants of disease?. Int J Epidemiol.

[7] Thrift AP, Gong J, Peters U, Chang-Claude J, Rudolph A, Slattery ML (2015). Mendelian randomization study of body mass index and colorectal cancer risk. Cancer Epidemiol Biomark Prev.

[8] Jarvis D, Mitchell JS, Law PJ, Palin K, Tuupanen S, Gylfe A (2016). Mendelian randomisation analysis strongly implicates adiposity with risk of developing colorectal cancer. Br J Cancer.

[9] Gao C, Patel CJ, Michailidou K, Peters U, Gong J, Schildkraut J (2016). Mendelian randomization study of adiposity-related traits and risk of breast, ovarian, prostate, lung and colorectal cancer. Int J Epidemiol.

[10] Gunter MJ, Riboli E (2018). Obesity and gastrointestinal cancers—where do we go from here?. Nature Rev Gastroenterol Hepatol.

[11] Dombrowski SU, Knittle K, Avenell A, Araujo-Soares V, Sniehotta FF (2014). Long term maintenance of weight loss with non-surgical interventions in obese adults: systematic review and meta-analyses of randomised controlled trials. BMJ..

[12] World Cancer Research Fund/American Institute for Cancer Research. Diet, nutrition, physical activity and colorectal cancer: continuous update project. 2017.

[13] Lawlor DA (2016). Commentary: two-sample Mendelian randomization: opportunities and challenges. Int J Epidemiol.

[14] Aschard H, Vilhjálmsson BJ, Joshi AD, Price AL, Kraft P (2015). Adjusting for heritable covariates can bias effect estimates in genome-wide association studies. Am J Hum Genet.

[15] Holmes MV, Ala-Korpela M, Davey SG (2017). Mendelian randomization in cardiometabolic disease: challenges in evaluating causality. Nat Rev Cardiol.

[16] Hartwig FP, Tilling K, Davey-Smith G, Lawlor DA, Borges M-CJB. Bias in two-sample Mendelian randomization by using covariable-adjusted summary associations. bioRxiv. 2019. p. 816363. 10.1093/ije/dyaa266 PMC8580279 33619569

[17] Holmes MV, Davey SG (2019). Problems in interpreting and using GWAS of conditional phenotypes illustrated by ‘alcohol GWAS’. Mol Psych.

[18] Rosen ED, Spiegelman BM (2014). What we talk about when we talk about fat. Cell..

[19] Kahn SE, Hull RL, Utzschneider KM (2006). Mechanisms linking obesity to insulin resistance and type 2 diabetes. Nature..

[20] Würtz P, Wang Q, Kangas AJ, Richmond RC, Skarp J, Tiainen M (2014). Metabolic signatures of adiposity in young adults: Mendelian randomization analysis and effects of weight change. PLoS Med.

[21] Rodriguez-Broadbent H, Law PJ, Sud A, Palin K, Tuupanen S, Gylfe A (2017). Mendelian randomisation implicates hyperlipidaemia as a risk factor for colorectal cancer. Int J Cancer.

[22] Song M, Lu Y, Gunter M, Murphy N, Banbury BL, Ma W (2018). Type 2 diabetes and glycemic traits in relation to colorectal cancer risk: a Mendelian randomization study.

[23] May-Wilson S, Sud A, Law PJ, Palin K, Tuupanen S, Gylfe A (2017). Pro-inflammatory fatty acid profile and colorectal cancer risk: a Mendelian randomisation analysis. Eur J Cancer.

[24] Würtz P, Kangas AJ, Soininen P, Lawlor DA, Davey Smith G, Ala-Korpela M. Quantitative serum NMR metabolomics in large-scale epidemiology: a primer on-omic technology. Am J Epidemiol. 2017:kwx016. 10.1093/aje/kwx016 PMC5860146 29106475

[25] Kettunen J, Demirkan A, Würtz P, Draisma HH, Haller T, Rawal R (2016). Genome-wide study for circulating metabolites identifies 62 loci and reveals novel systemic effects of LPA. Nat Commun.

[26] Huyghe JR, Bien SA, Harrison TA, Kang HM, Chen S, Schmit SL (2019). Discovery of common and rare genetic risk variants for colorectal cancer. Nature Genet.

[27] Davey Smith G, Hemani G (2014). Mendelian randomization: genetic anchors for causal inference in epidemiological studies. Hum Mol Gen.

[28] Bowden J, Davey Smith G, Haycock PC, Burgess S (2016). Consistent estimation in Mendelian randomization with some invalid instruments using a weighted median estimator. Genet Epidemiol.

[29] Hartwig FP, Davey Smith G, Bowden J (2017). Robust inference in summary data Mendelian randomization via the zero modal pleiotropy assumption. Int J Epidemiol.

[30] Bowden J, Davey Smith G, Burgess S (2015). Mendelian randomization with invalid instruments: effect estimation and bias detection through Egger regression. Int J Epidemiol.

[31] Sterne JA, Davey SG (2001). Sifting the evidence—what’s wrong with significance tests?. BMJ..

[32] Wasserstein RL, Lazar NA (2016). The ASA’s statement on p-values: context, process, and purpose. Am Statistician.

[33] Pulit SL, Stoneman C, Morris AP, Wood AR, Glastonbury CA, Tyrrell J, et al. Metaanalysis of genome-wide association studies for body fat distribution in 694 649 individuals of European ancestry. Hum Mol Gen. 2019;28(1):166–74. 10.1093/hmg/ddy327 PMC6298238 30239722

[34] Shim H, Chasman DI, Smith JD, Mora S, Ridker PM, Nickerson DA (2015). A multivariate genome-wide association analysis of 10 LDL subfractions, and their response to statin treatment, in 1868 Caucasians. PLoS One.

[35] Haycock PC, Burgess S, Wade KH, Bowden J, Relton C, Davey SG (2016). Best (but oft-forgotten) practices: the design, analysis, and interpretation of Mendelian randomization studies. Am J Clin Nutr.

[36] Hemani G, Zheng J, Elsworth B, Wade KH, Haberland V, Baird D (2018). The MR-base platform supports systematic causal inference across the human phenome. eLife..

[37] Burgess S, Bowden J, Fall T, Ingelsson E, Thompson SG (2017). Sensitivity analyses for robust causal inference from Mendelian randomization analyses with multiple genetic variants. Epidemiology..

[38] Wald A (1940). The fitting of straight lines if both variables are subject to error. Ann Mathematical Statistics.

[39] Bowden J, Hemani G, Davey Smith GJAjoe. Invited commentary: Detecting individual and global horizontal pleiotropy in Mendelian randomization—a job for the humble heterogeneity statistic? 2018;187(12):2681–5. 10.1093/aje/kwy185 PMC6269239 30188969

[40] Zheng J, Baird D, Borges M-C, Bowden J, Hemani G, Haycock P (2017). Recent developments in Mendelian randomization studies. Curr Epidemiol Rep.

[41] Benjamini Y, Hochberg Y (1995). Controlling the false discovery rate: a practical and powerful approach to multiple testing. J Royal Statistic Soc: Series B (Methodological).

[42] Sanderson E, Davey Smith G, Windmeijer F, Bowden J. An examination of multivariable Mendelian randomization in the single-sample and two-sample summary data settings. Int J Epidemiol. 2018;dyy262:1–15. 10.1093/ije/dyy262 PMC6734942 30535378

[43] Kujala UM, Mäkinen V-P, Heinonen I, Soininen P, Kangas AJ, Leskinen TH, et al. Long-term leisure-time physical activity and serum metabolome. Circulation. 2012:CIRCULATIONAHA. 112.105551. 10.1161/CIRCULATIONAHA.112.105551 23258601

[44] Locke AE, Kahali B, Berndt SI, Justice AE, Pers TH, Day FR (2015). Genetic studies of body mass index yield new insights for obesity biology. Nature..

[45] Shungin D, Winkler TW, Croteau-Chonka DC, Ferreira T, Locke AE, Mägi R (2015). New genetic loci link adipose and insulin biology to body fat distribution. Nature..

[46] Sanderson E, Spiller W, Bowden J. Testing and correcting for weak and pleiotropic instruments in two-sample multivariable Mendelian randomisation. BioRxiv. 2020. 10.1101/2020.04.02.021980. 10.1002/sim.9133 PMC9479726 34338327

[47] Murphy N, Jenab M, Gunter MJ (2018). Adiposity and gastrointestinal cancers: epidemiology, mechanisms and future directions. Nat Rev Gastroenterol Hepatol.

[48] Wells JC (2007). Sexual dimorphism of body composition. Best Pract Res Clin Endocrinol Metab.

[49] Lovejoy J, Champagne C, De Jonge L, Xie H, Smith S (2008). Increased visceral fat and decreased energy expenditure during the menopausal transition. Int J Obes.

[50] Bell JA, Carslake D, O’Keeffe LM, Frysz M, Howe LD, Hamer M (2018). Associations of body mass and fat indexes with cardiometabolic traits. J Am Coll Cardiol.

[51] Flegal KM, Shepherd JA, Looker AC, Graubard BI, Borrud LG, Ogden CL (2009). Comparisons of percentage body fat, body mass index, waist circumference, and waist-stature ratio in adults. Am J Clin Nutr.

[52] Wei H-J, Zeng R, Lu J-H, Lai W-FT, Chen W-H, Liu H-Y (2015). Adipose-derived stem cells promote tumor initiation and accelerate tumor growth by interleukin-6 production. Oncotarget..

[53] Hotamisligil G (2006). Inflammation and metabolic disorders. Nature..

[54] Rinaldi S, Cleveland R, Norat T, Biessy C, Rohrmann S, Linseisen J, et al. Serum levels of IGF-I, IGFBP-3 and colorectal cancer risk: results from the EPIC cohort, plus a meta-analysis of prospective studies. Int J Cancer. 2010;126:NA-NA. 10.1002/ijc.24927 19810099

[55] Tran TT, Naigamwalla D, Oprescu AI, Lam L, McKeown-Eyssen G, Bruce WR (2006). Hyperinsulinemia, but not other factors associated with insulin resistance, acutely enhances colorectal epithelial proliferation in vivo. Endocrinol.

[56] Kiunga GA, Raju J, Sabljic N, Bajaj G, Good CK, Bird RP (2004). Elevated insulin receptor protein expression in experimentally induced colonic tumors. Cancer Lett.

[57] Kaaks R, Toniolo P, Akhmedkhanov A, Lukanova A, Biessy C, Dechaud H (2000). Serum C-peptide, insulin-like growth factor (IGF)-I, IGF-binding proteins, and colorectal cancer risk in women. J Natl Cancer Inst.

[58] Murphy N, Carreras-Torres R, Song M, Chan AT, Martin RM, Papadimitriou N, et al. Circulating levels of insulin-like growth factor 1 and insulin-like growth factor binding protein 3 associate with risk of colorectal cancer based on serologic and Mendelian randomization analyses. Gastroenterology. 2019;158(5):1300–312.e20. 10.1053/j.gastro.2019.12.020. 10.1053/j.gastro.2019.12.020 PMC7152801 31884074

[59] Burgess S, Thompson SG, CRP CHD Genetics Collaboration. Avoiding bias from weak instruments in Mendelian randomization studies. International Journal of Epidemiology 2011;40(3):755–64. 10.1093/ije/dyr036 21414999

[60] Gonzalez EC, Roetzheim RG, Ferrante JM, Campbell R (2001). Predictors of proximal vs. distal colorectal cancers. Dis Colon Rectum.

[61] Jacobs ET, Thompson PA, Martínez MaE. Diet, gender, and colorectal neoplasia. J Clin Gastroenterol 2007;41:731–746. 10.1097/MCG.0b013e3180338e56 17700421

[62] Okubo R, Masuda H, Nemoto N. p53 mutation found to be a significant prognostic indicator in distal colorectal cancer. Oncol Rep. 2001;8(3):509-14. 10.3892/or.8.3.509 11295071

[63] Pekow J, Meckel K, Dougherty U, Butun F, Mustafi R, Lim J (2015). Tumor suppressors miR-143 and miR-145 and predicted target proteins API5, ERK5, K-RAS, and IRS-1 are differentially expressed in proximal and distal colon. Am J Physiol-Gastrointestinal Liver Physiol.

[64] Missiaglia E, Jacobs B, D’Ario G, Di Narzo AF, Soneson C, Budinska E (2014). Distal and proximal colon cancers differ in terms of molecular, pathological, and clinical features. Ann Oncol.

[65] Dale KM, Coleman CI, Henyan NN, Kluger J, White CM (2006). Statins and cancer risk: a meta-analysis. JAMA..

[66] Liu Y, Tang W, Wang J, Xie L, Li T, He Y, et al. Association between statin use and colorectal cancer risk: a meta-analysis of 42 studies. Cancer Causes Control. 2014;25(2):237–49. 10.1007/s10552-013-0326-6 24265089

[67] Lytras T, Nikolopoulos G, Bonovas S. Statins and the risk of colorectal cancer: An updated systematic review and meta-analysis of 40 studies. World J Gastroenterol. 2014;20(7):1858–70. 10.3748/wjg.v20.i7.1858 PMC3930985 24587664

[68] Yao X, Tian Z (2015). Dyslipidemia and colorectal cancer risk: a meta-analysis of prospective studies. Cancer Causes Control.

[69] Lee S, Zhang C, Kilicarslan M, Piening BD, Bjornson E, Hallström BM (2016). Integrated network analysis reveals an association between plasma mannose levels and insulin resistance. Cell Metab.

[70] Lawlor DA, Tilling K, Davey SG (2016). Triangulation in aetiological epidemiology. Int J Epidemiol.

[71] Burgess S, Davies NM, Thompson SG (2016). Bias due to participant overlap in two-sample Mendelian randomization. Genet Epidemiol.

