# Supplementary material for: Adiposity, metabolites, and colorectal cancer risk: Mendelian randomization study
Source: BMC Med. 2020 Dec 17;18:396. doi: 10.1186/s12916-020-01855-9 (PMC7745469; doi:10.1186/s12916-020-01855-9)
Supplement: Supplementary file 2 — Additional file 2: Figure S1. Scatter plot of SNP-BMI and SNP-CRC associations. Figure S2. Scatter plot of SNP-BMI and SNP-CRC associations (female specific). Figure S3. Scatter plot of SNP-BMI and SNP-CRC associations (male specific). Figure S4. Scatter plot of SNP-BMI and SNP-colon cancer associations. Figure S5. Scatter plot of SNP-BMI and SNP-proximal colon cancer associations. Figure S6. Scatter plot of SNP-BMI and SNP-distal colon cancer associations. Figure S7. Scatter plot of SNP-BMI and SNP-rectal cancer associations. Figure S8. Forest plot showing individual SNP (black) and combined MR estimates (red; Egger and IVW) for the effect of BMI on CRC. Figure S9. Forest plot showing individual SNP (black) and combined MR estimates (red; Egger and IVW) for the effect of BMI on CRC (female specific). Figure S10. Forest plot showing individual SNP (black) and combined MR estimates (red; Egger and IVW) for the effect of BMI on CRC (male specific). Figure S11. Forest plot showing individual SNP (black) and combined MR estimates (red; Egger and IVW) for the effect of BMI on colon cancer. Figure S12. Forest plot showing individual SNP (black) and combined MR estimates (red; Egger and IVW) for the effect of BMI on proximal colon cancer. Figure S13. Forest plot showing individual SNP (black) and combined MR estimates (red; Egger and IVW) for the effect of BMI on distal colon cancer. Figure S14. Forest plot showing individual SNP (black) and combined MR estimates (red; Egger and IVW) for the effect of BMI on rectal cancer. Figure S15. Leave-one-out plot showing the association between BMI and CRC, following SNP-by-SNP removal from the model. Figure S16. Leave-one-out plot showing the association between BMI and CRC (femalespecific), following SNP-by-SNP removal from the model. Figure S17. Leave-one-out plot showing the association between BMI and CRC (malespecific), following SNP-by-SNP removal from the model. Figure S18. Leave-one-out plot showing the association between [file 12916_2020_1855_MOESM2_ESM.pdf]

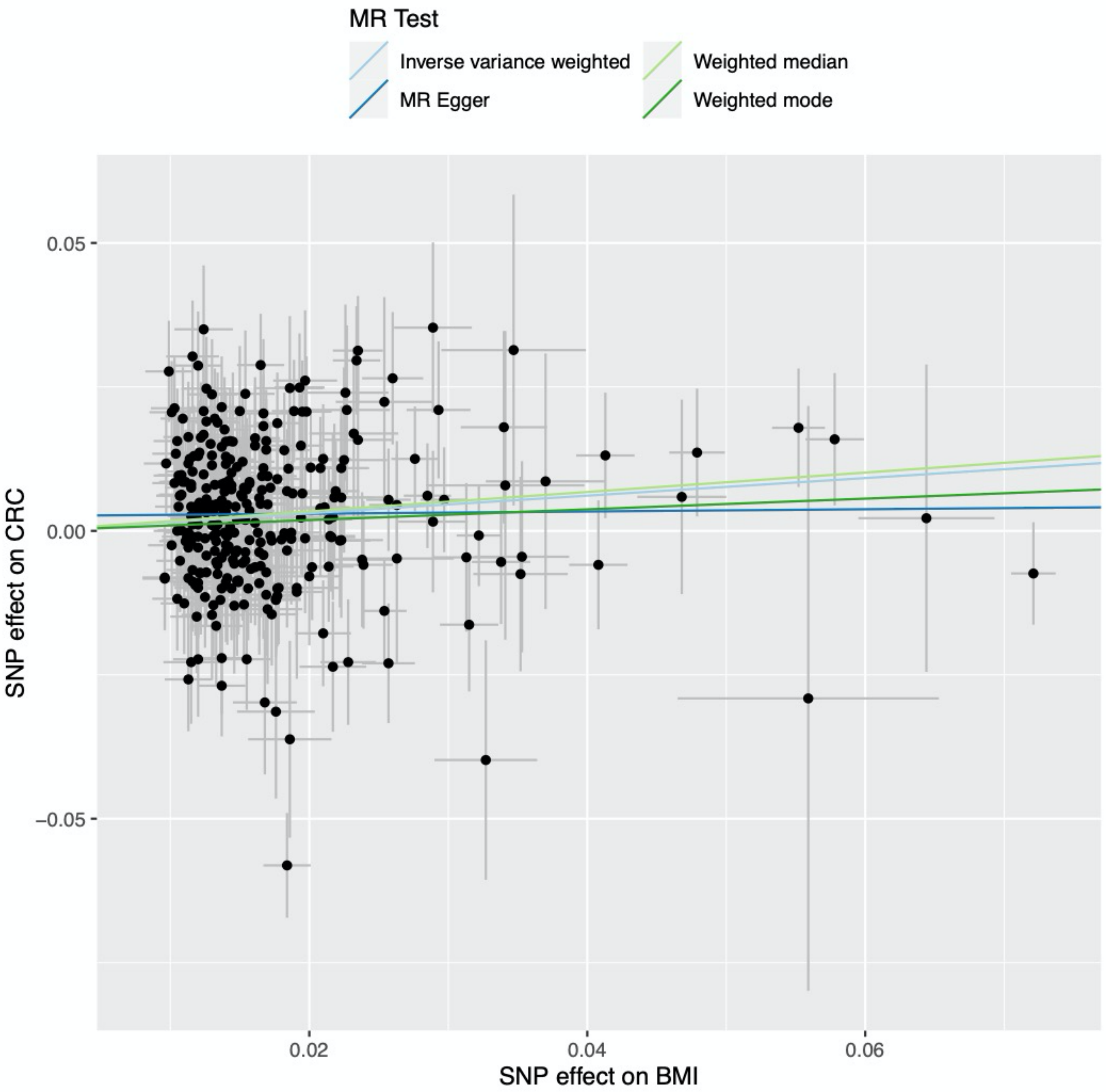

Figure S1. Scatter plot of SNP-BMI and SNP-CRC associations

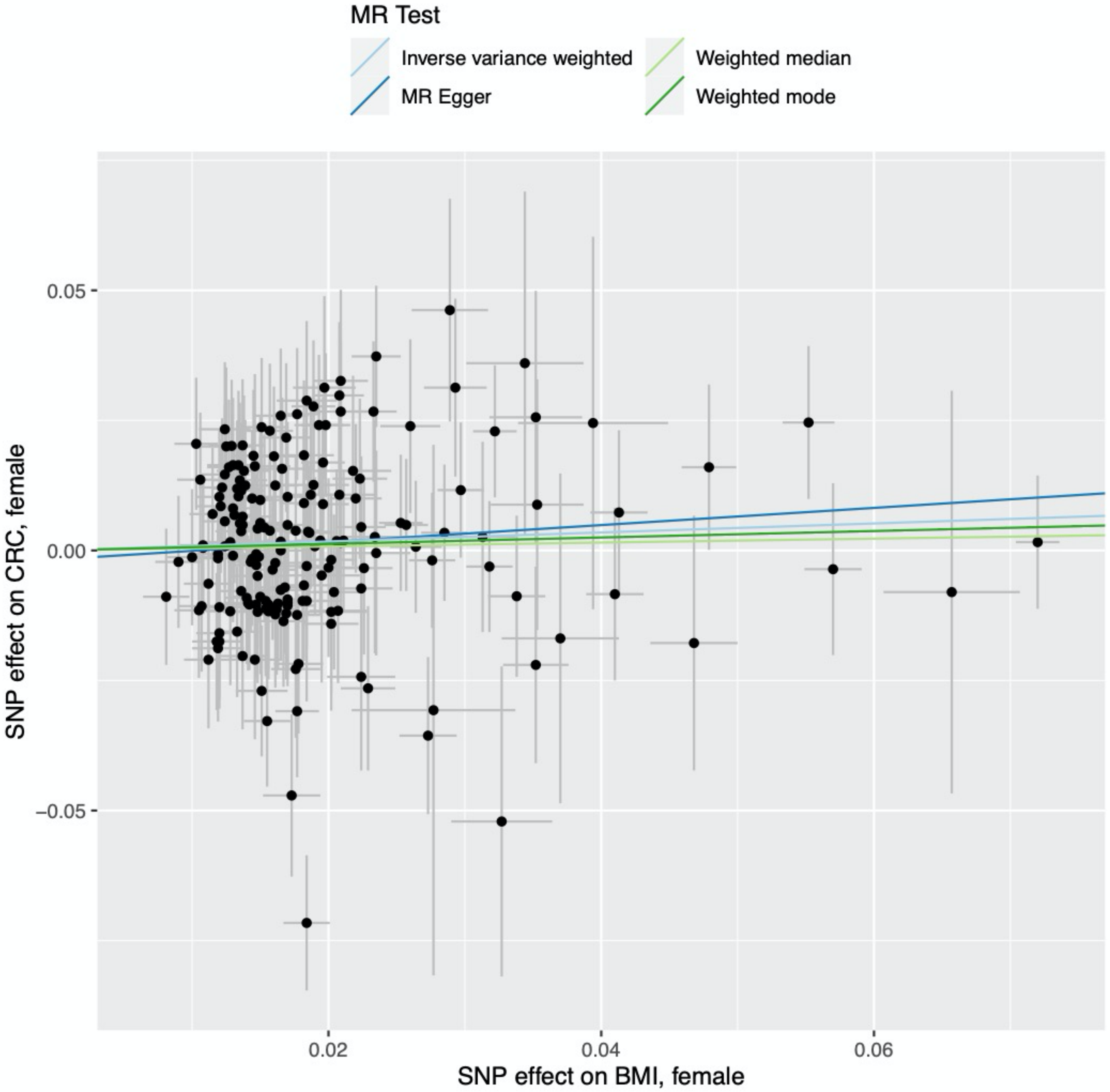

Figure S2. Scatter plot of SNP-BMI and SNP-CRC associations (female specific)

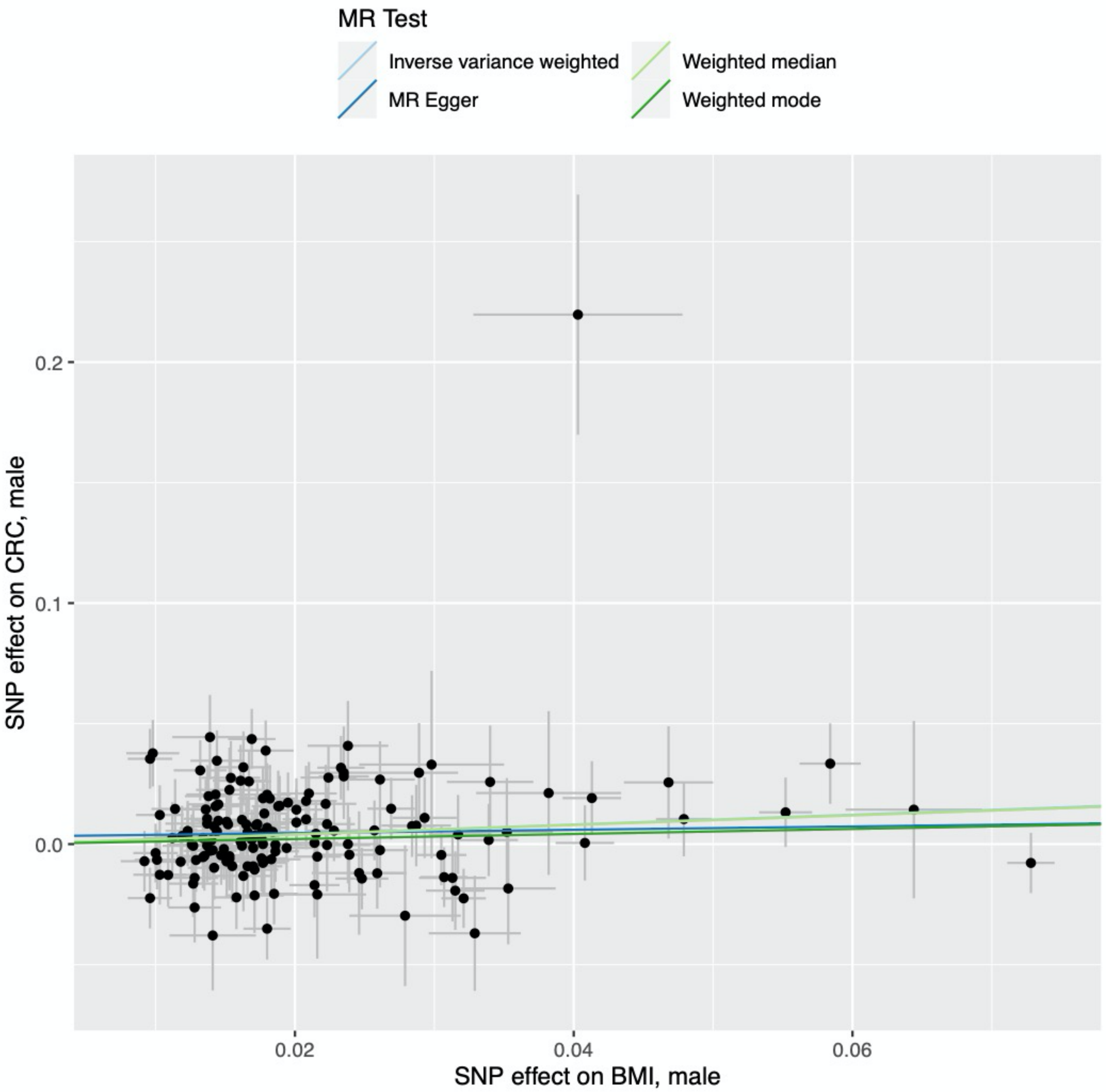

Figure S3. Scatter plot of SNP-BMI and SNP-CRC associations (male specific)

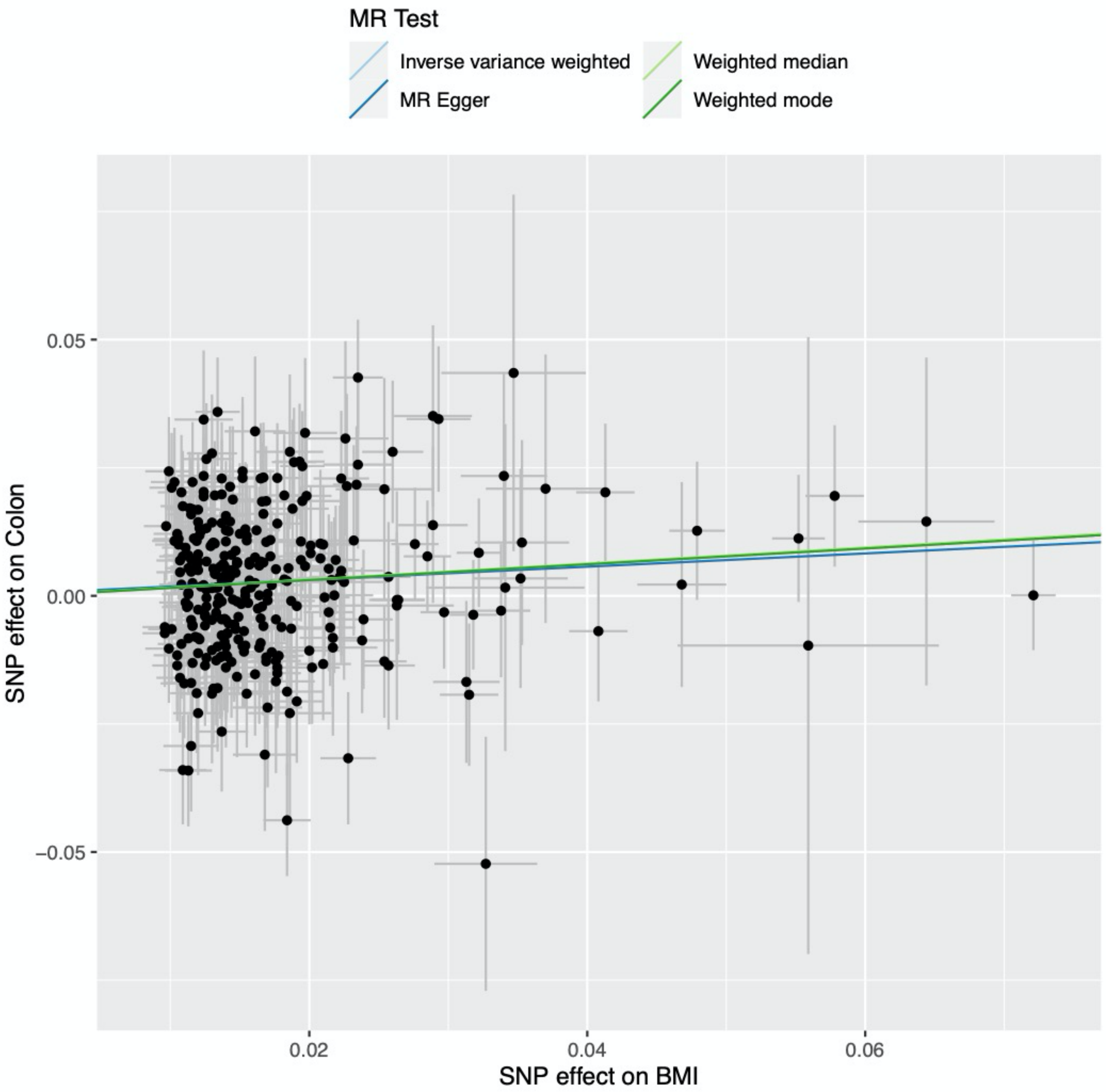

Figure S4. Scatter plot of SNP-BMI and SNP-colon cancer associations

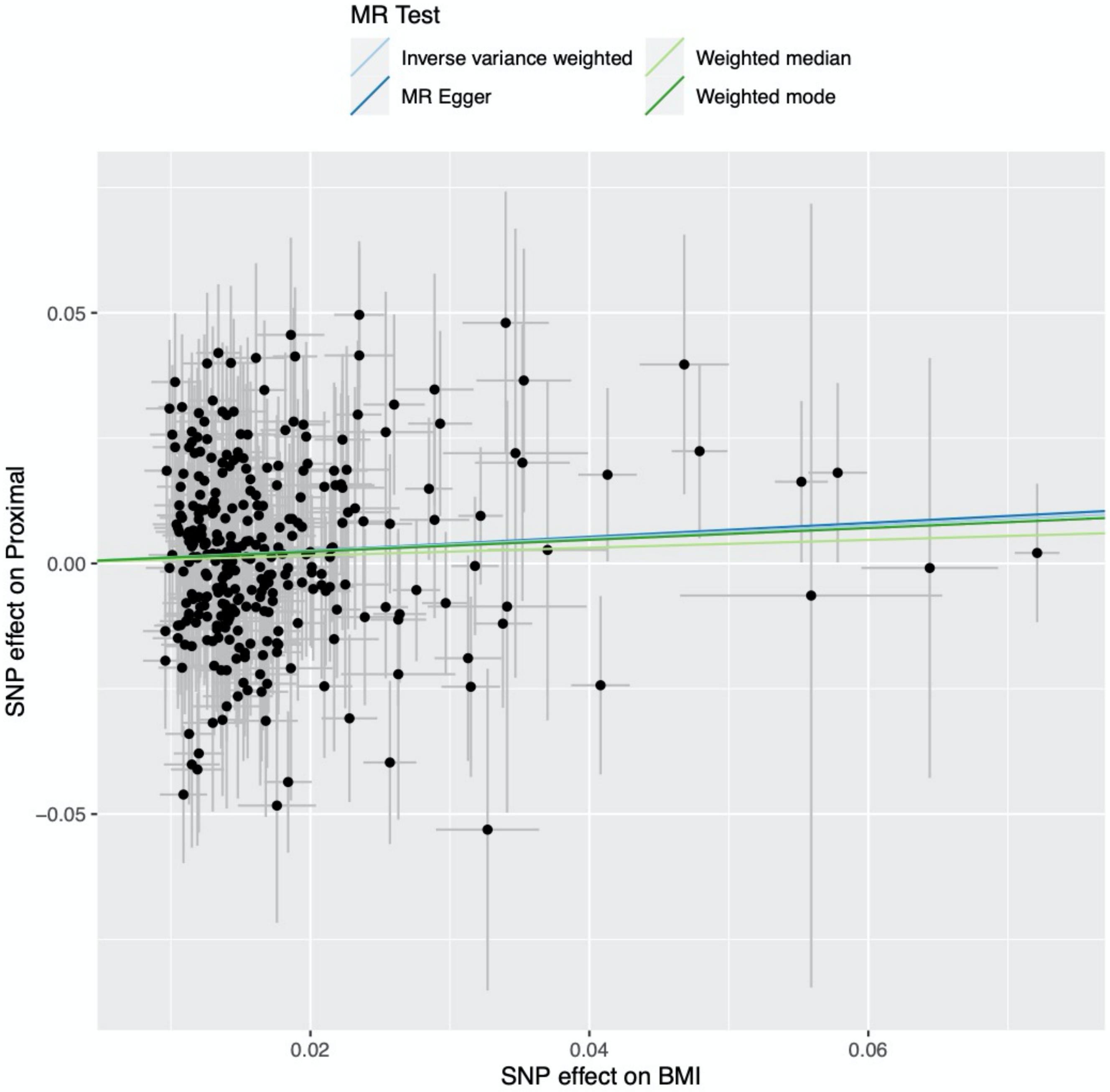

Figure S5. Scatter plot of SNP-BMI and SNP-proximal colon cancer associations

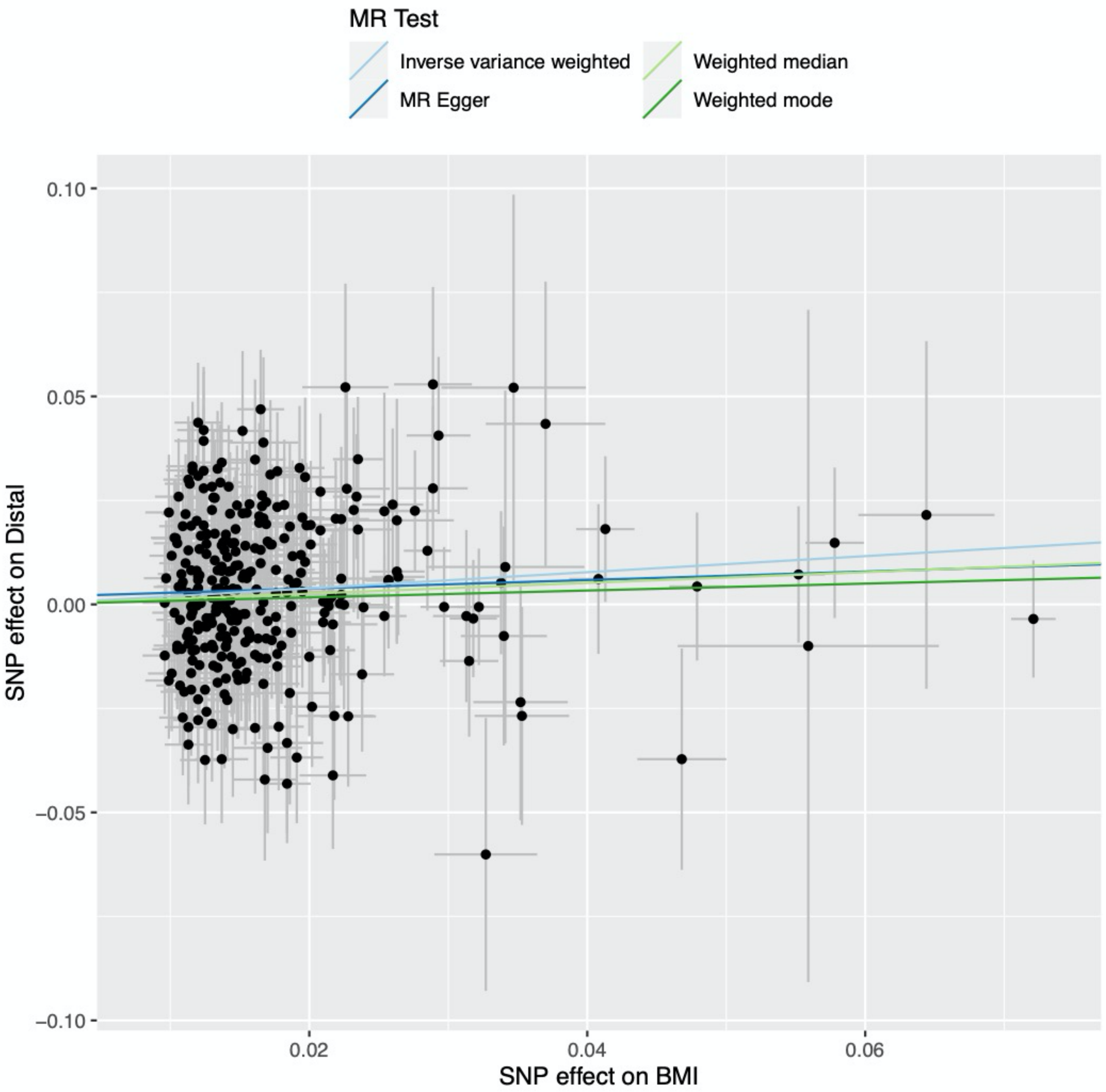

Figure S6. Scatter plot of SNP-BMI and SNP-distal colon cancer associations

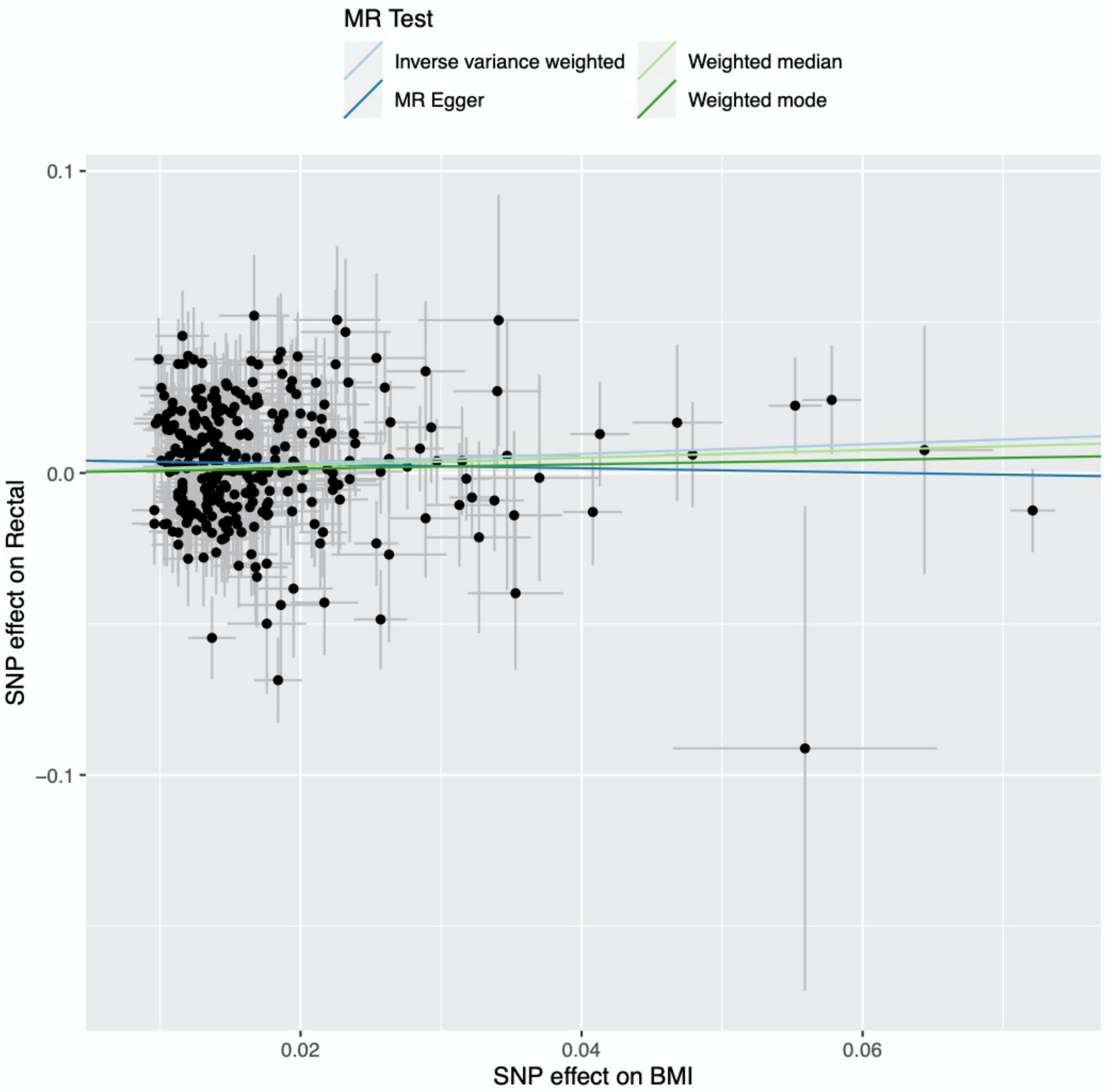

Figure S7. Scatter plot of SNP-BMI and SNP-rectal cancer associations

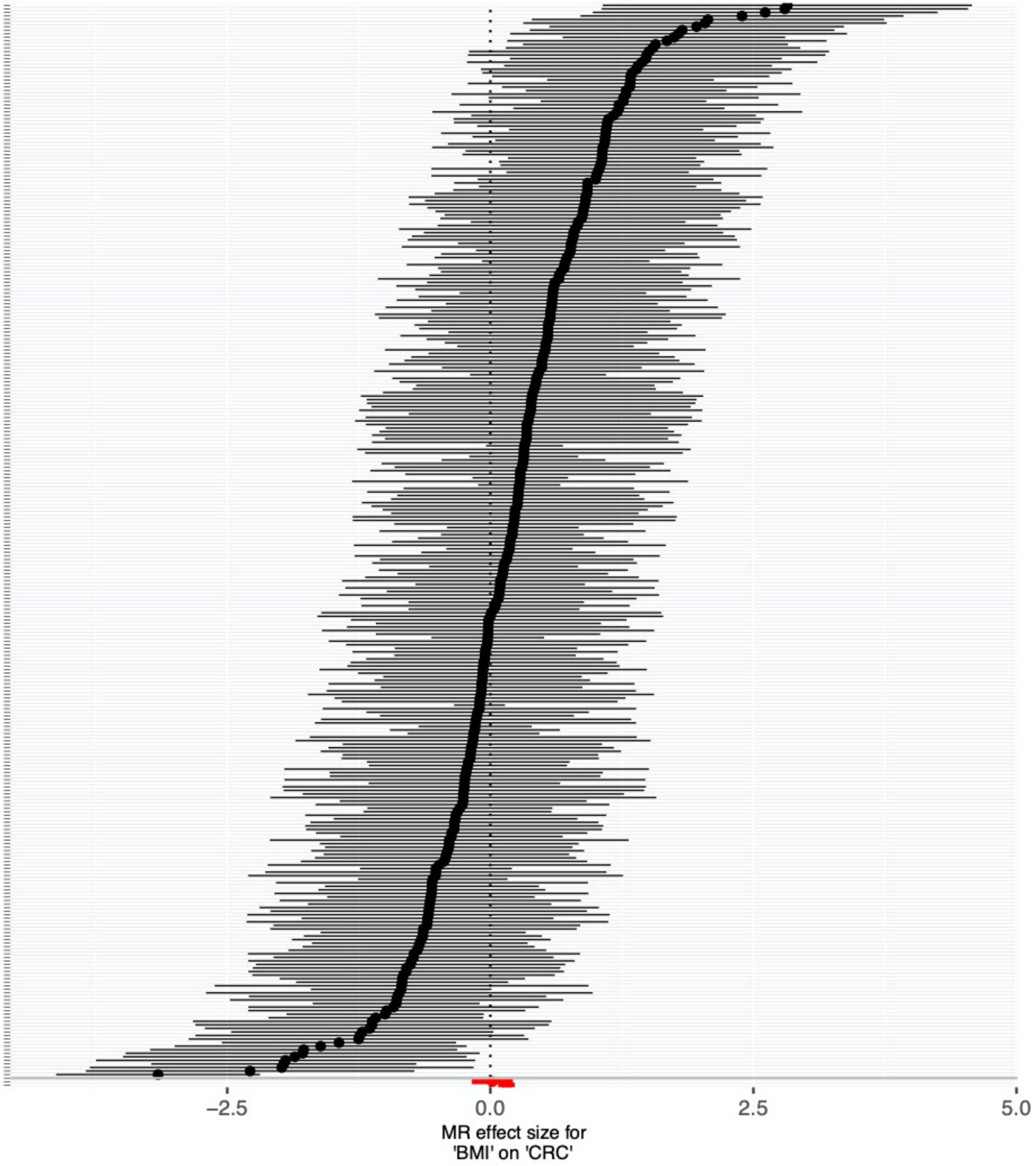

Figure S8. Forest plot showing individual SNP (black) and combined MR estimates (red; Egger and IVW) for the effect of BMI on CRC

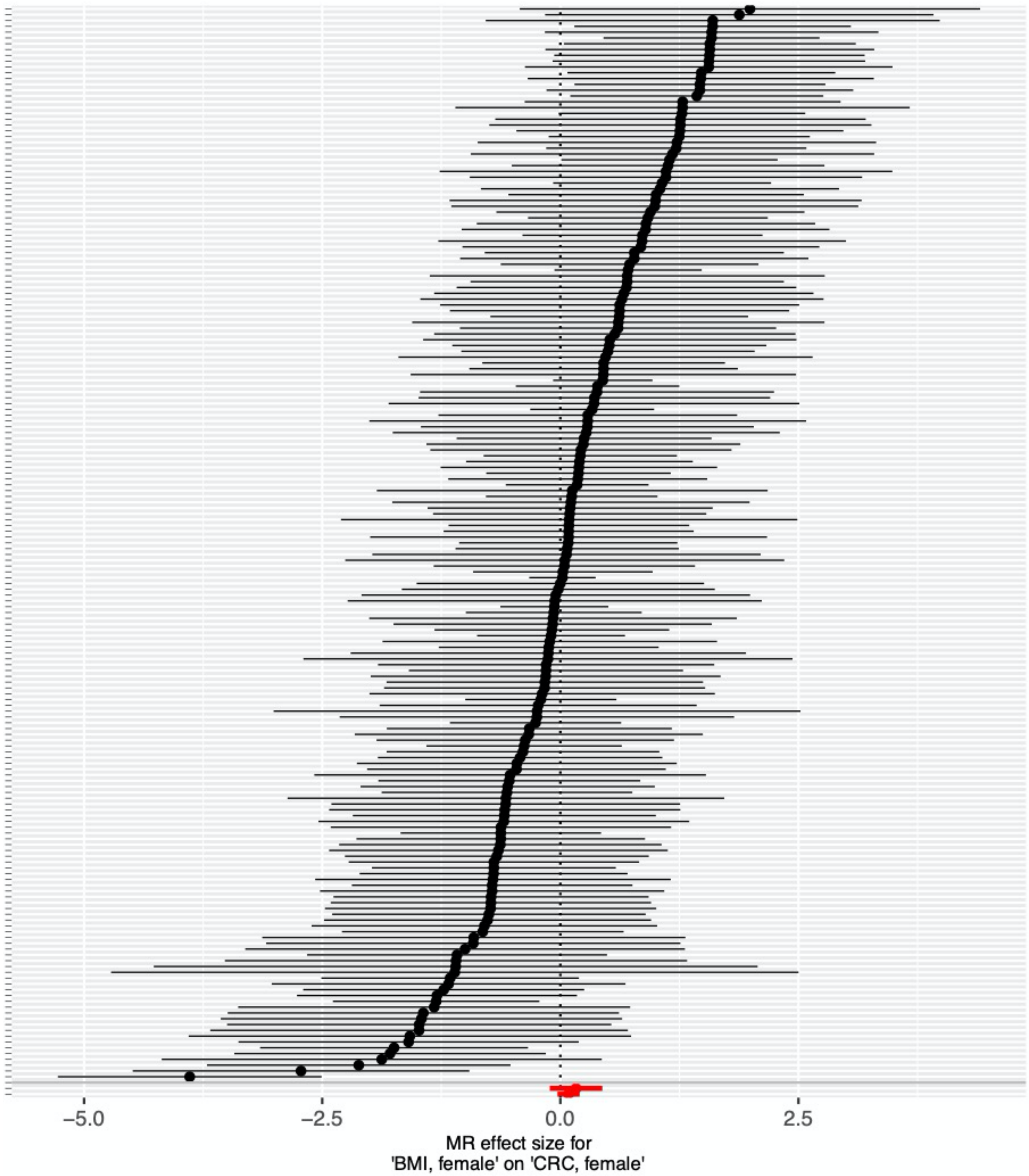

Figure S9. Forest plot showing individual SNP (black) and combined MR estimates (red; Egger and IVW) for the effect of BMI on CRC (female specific)

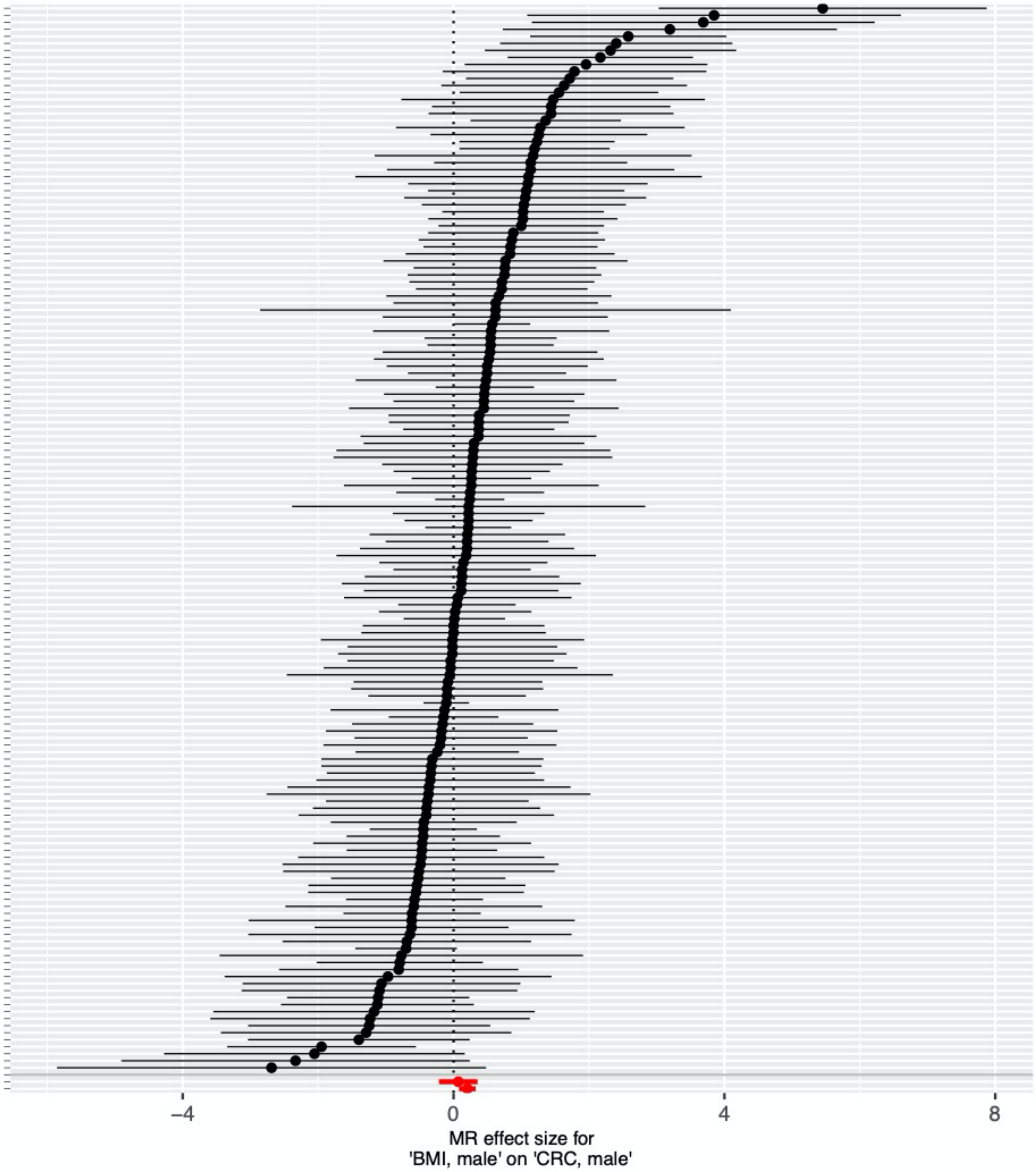

Figure S10. Forest plot showing individual SNP (black) and combined MR estimates (red; Egger and IVW) for the effect of BMI on CRC (male specific)

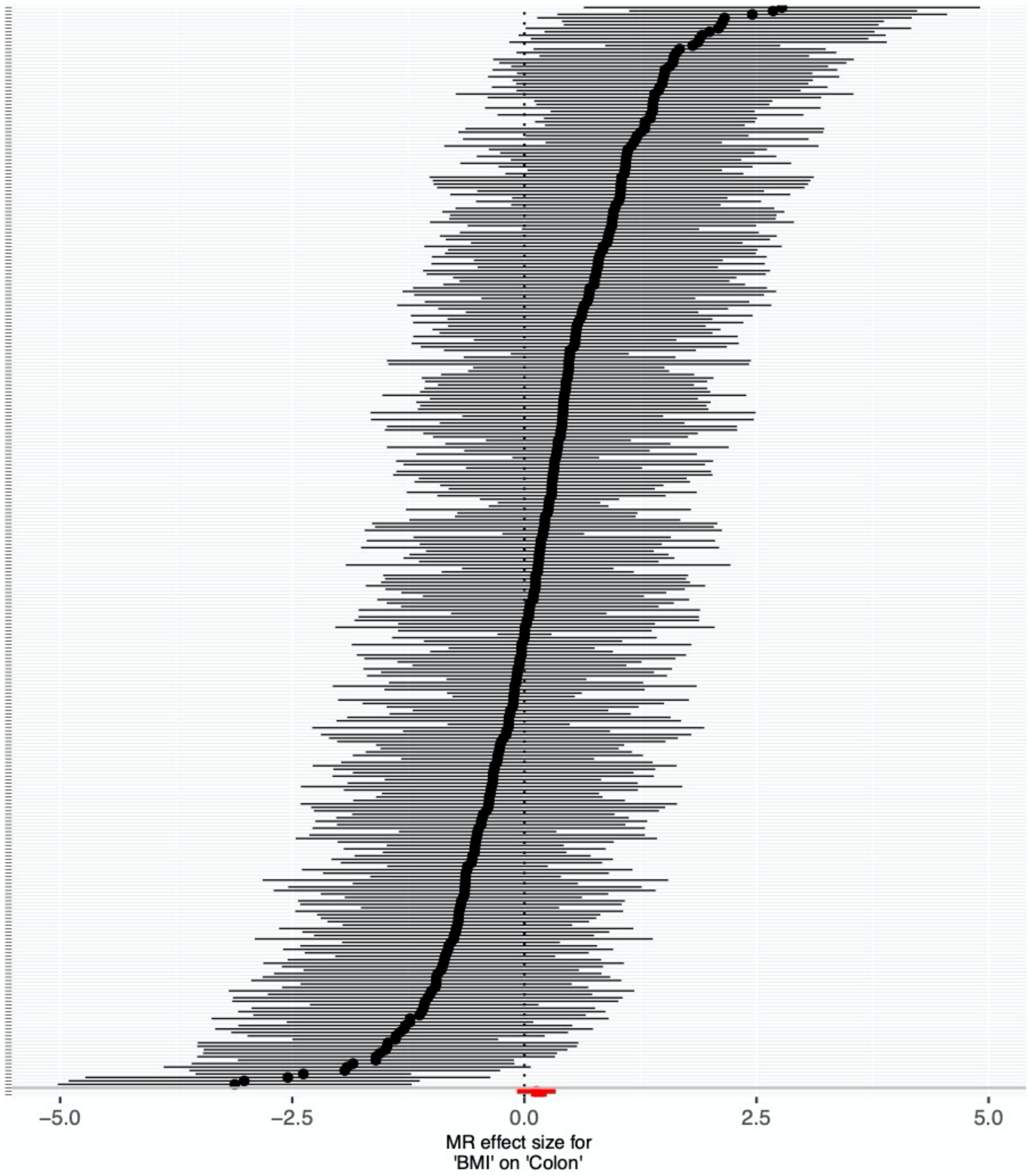

Figure S11. Forest plot showing individual SNP (black) and combined MR estimates (red; Egger and IVW) for the effect of BMI on colon cancer

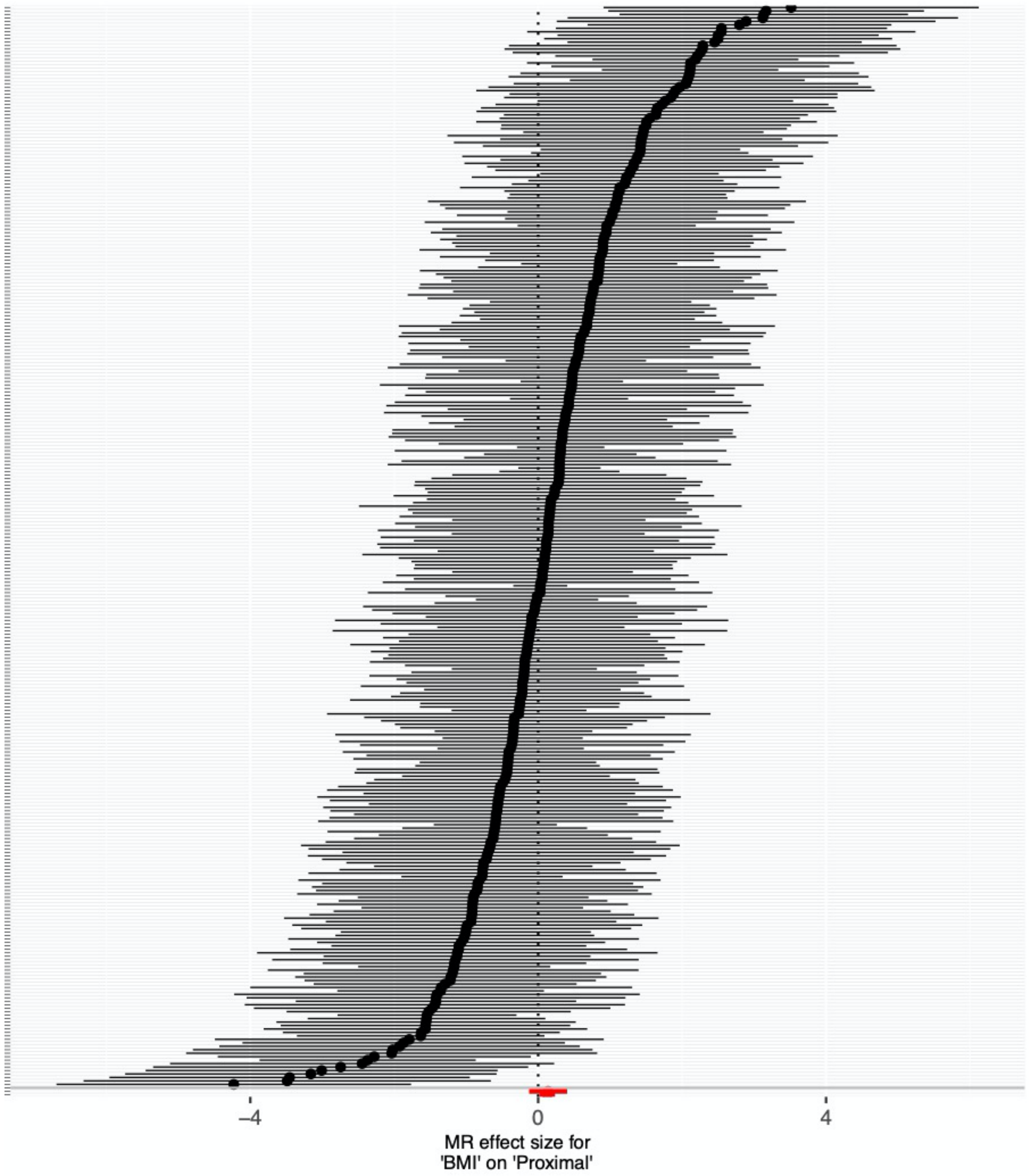

Figure S12. Forest plot showing individual SNP (black) and combined MR estimates (red; Egger and IVW) for the effect of BMI on proximal colon cancer

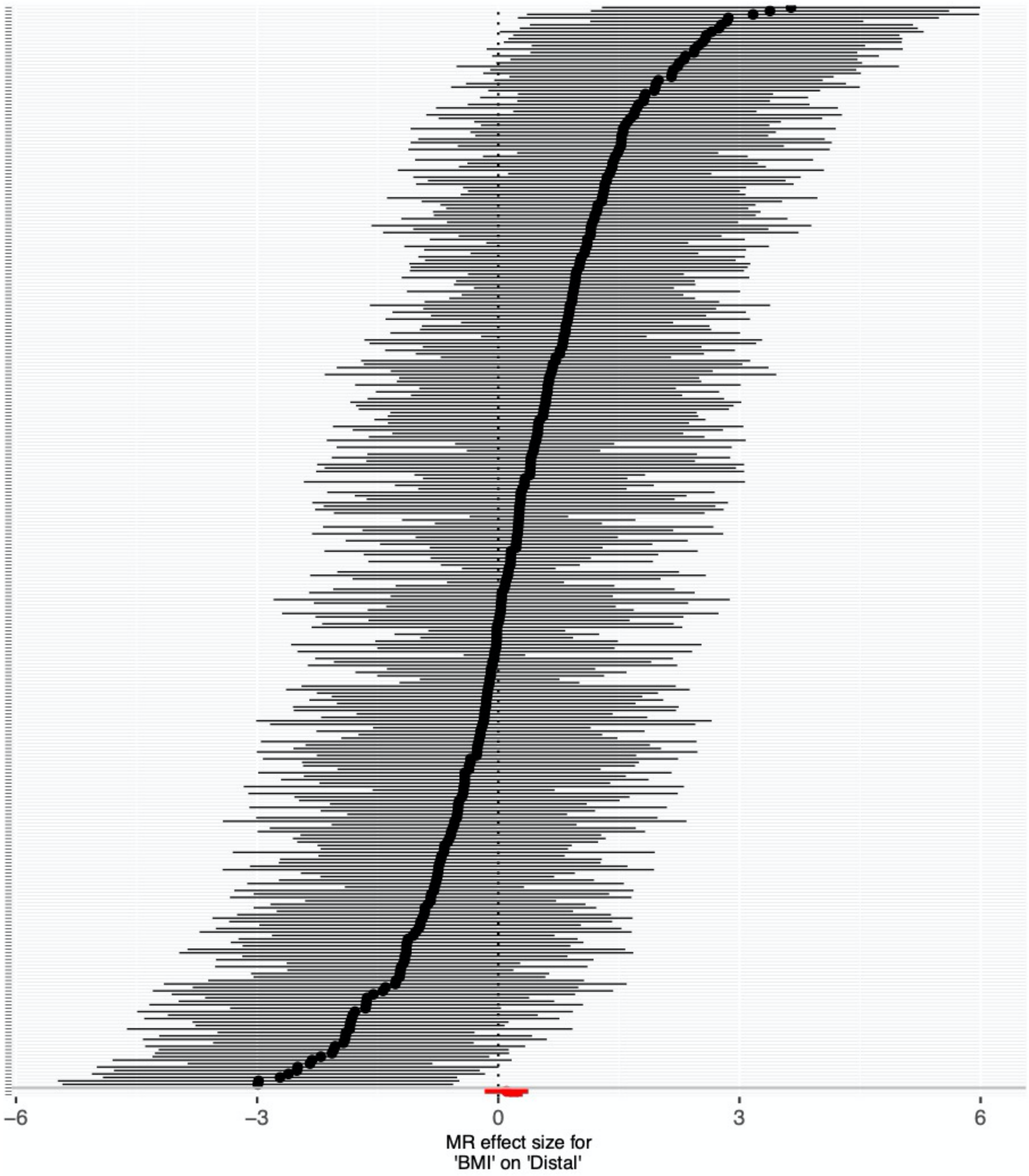

Figure S13. Forest plot showing individual SNP (black) and combined MR estimates (red; Egger and IVW) for the effect of BMI on distal colon cancer

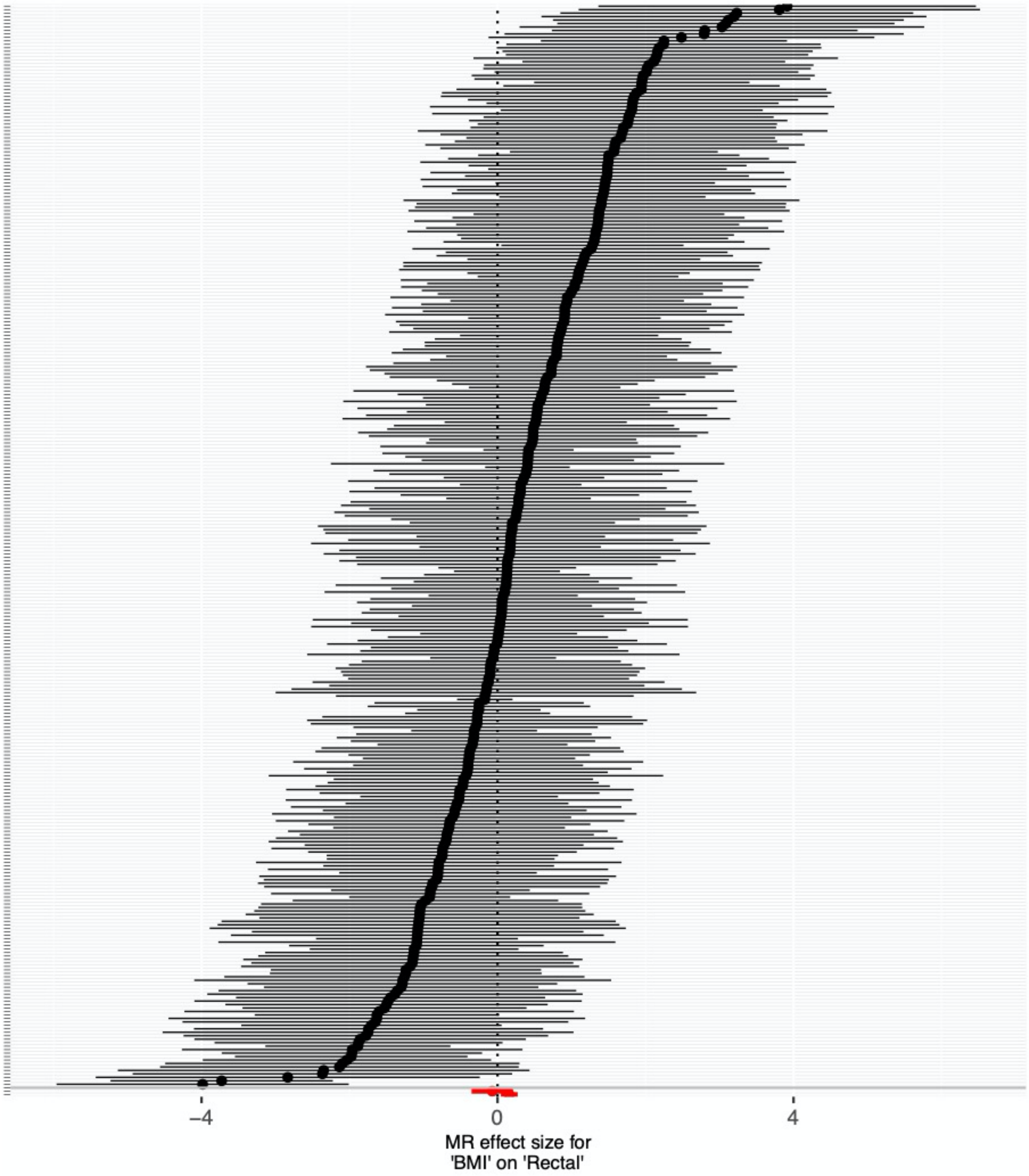

Figure S14. Forest plot showing individual SNP (black) and combined MR estimates (red; Egger and IVW) for the effect of BMI on rectal cancer

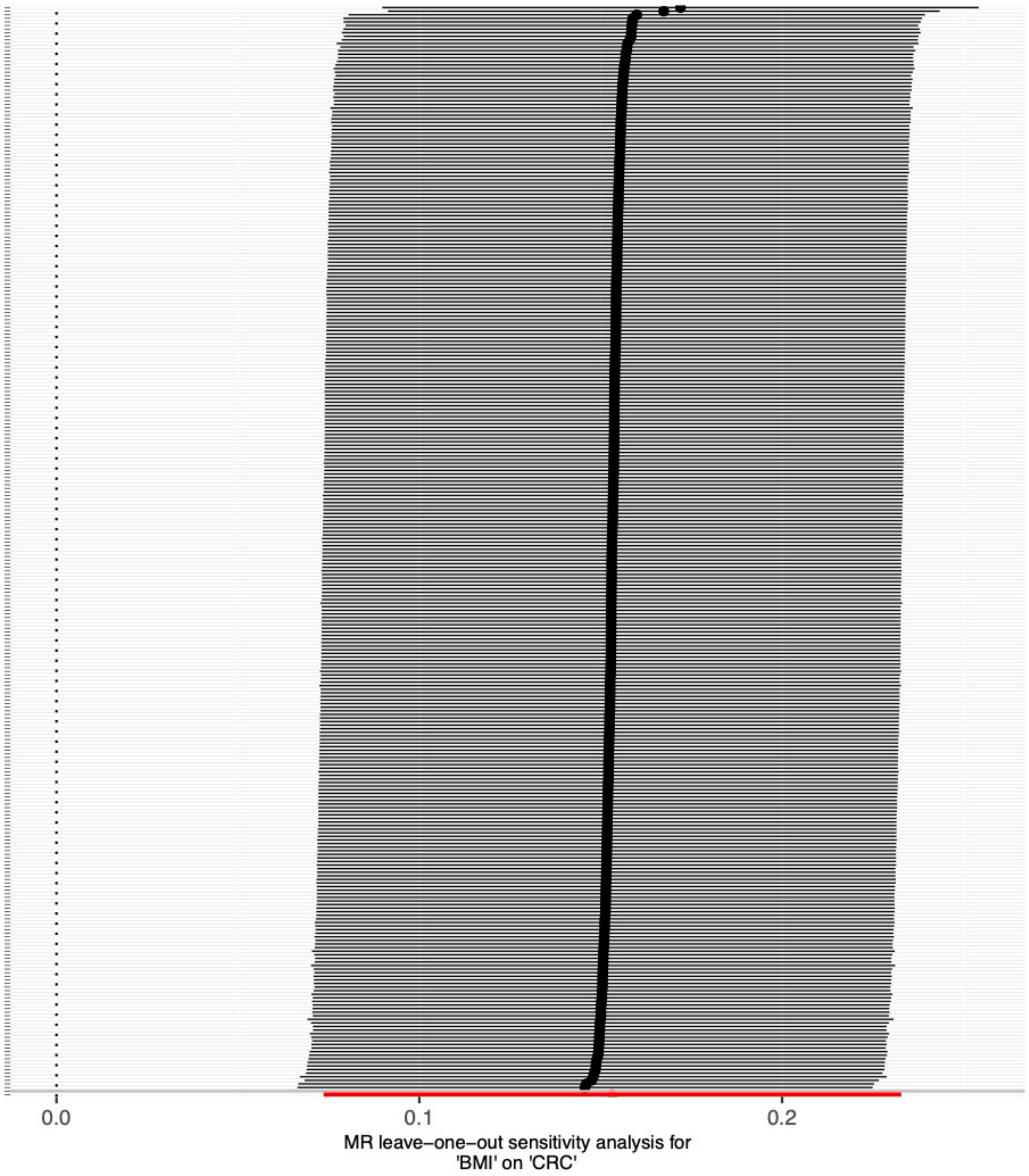

Figure S15. Leave-one-out plot showing the association between BMI and CRC, following SNP-by-SNP removal from the model

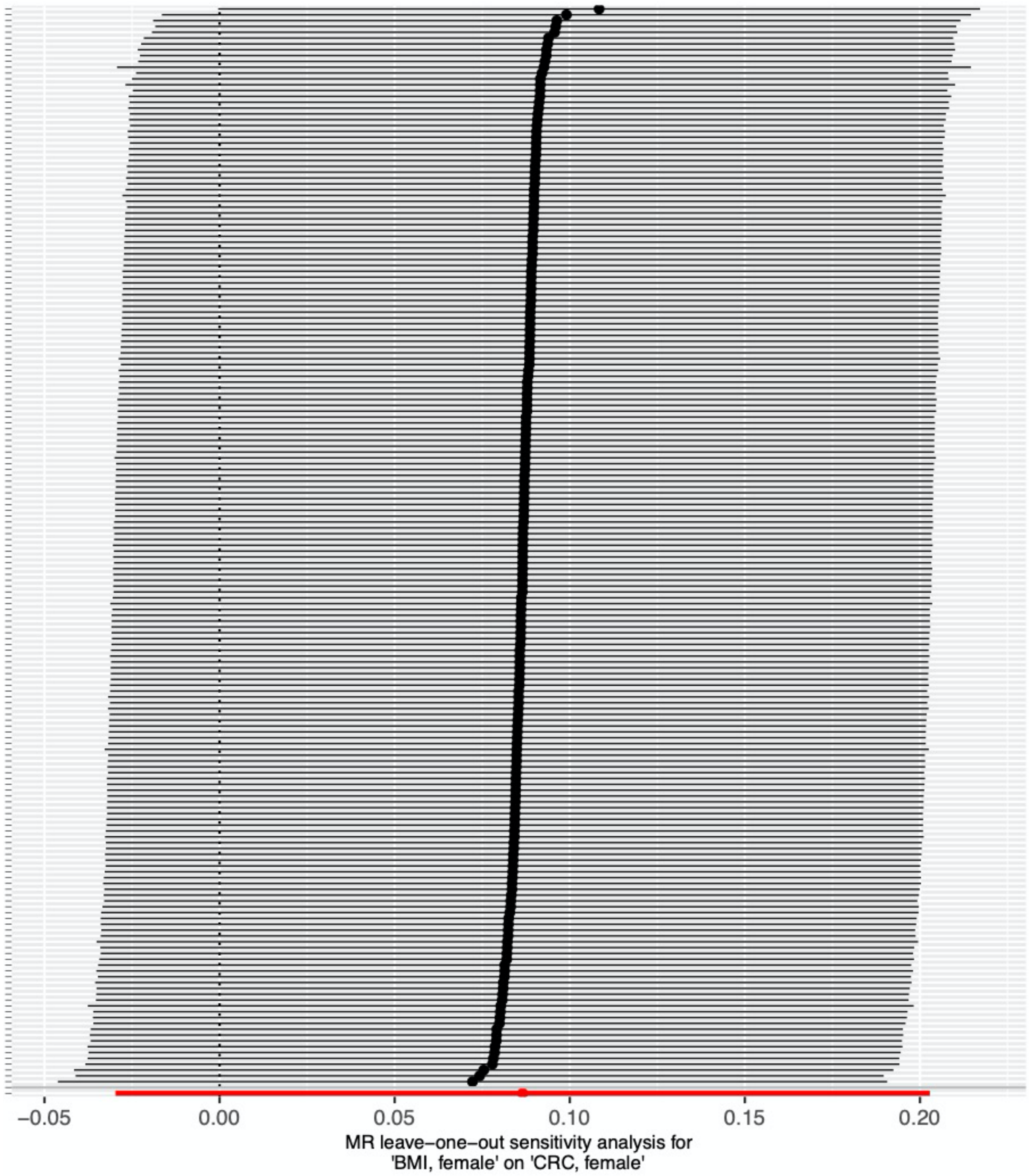

Figure S16. Leave-one-out plot showing the association between BMI and CRC (female-specific), following SNP-by-SNP removal from the model

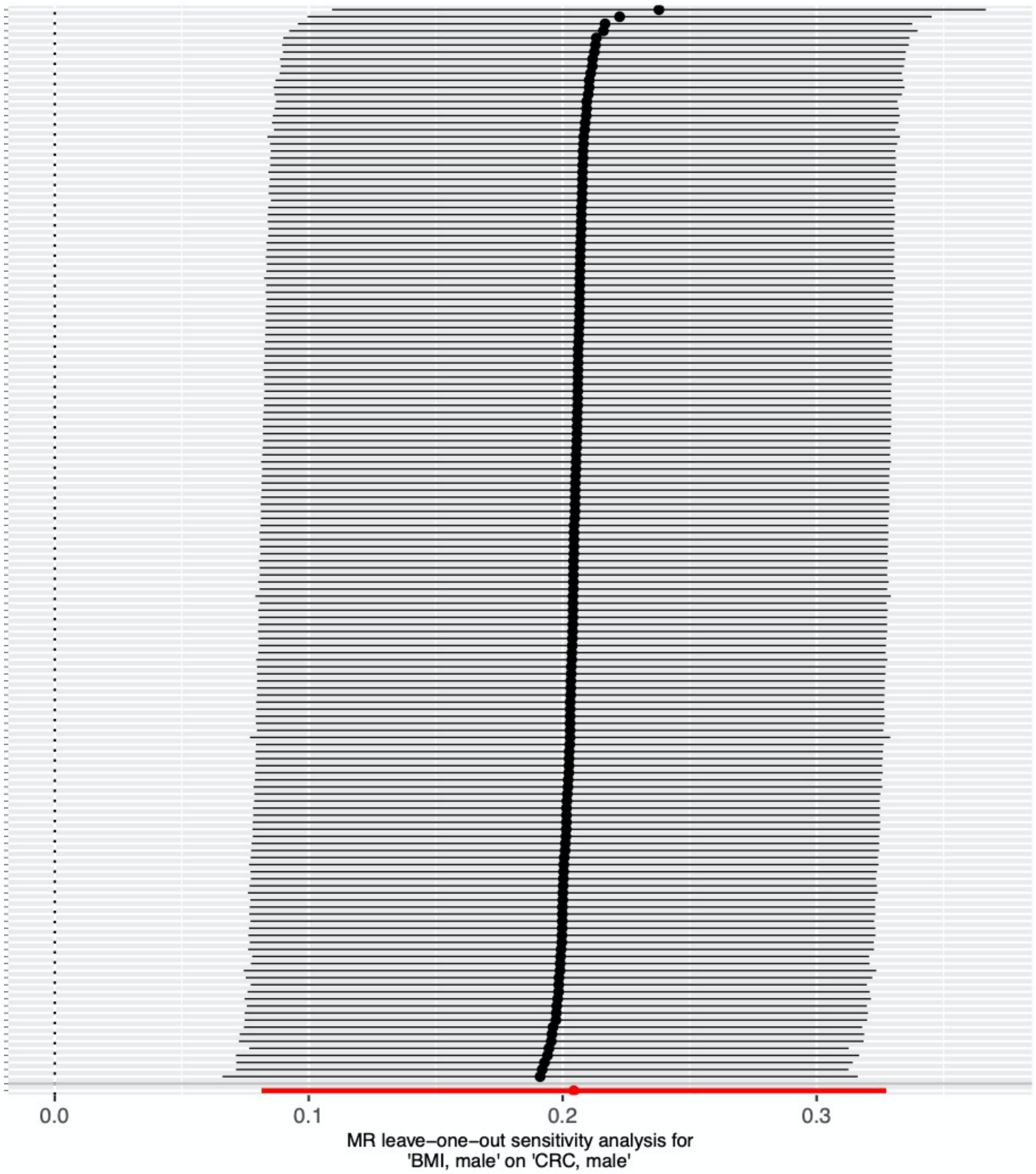

Figure S17. Leave-one-out plot showing the association between BMI and CRC (male-specific), following SNP-by-SNP removal from the model

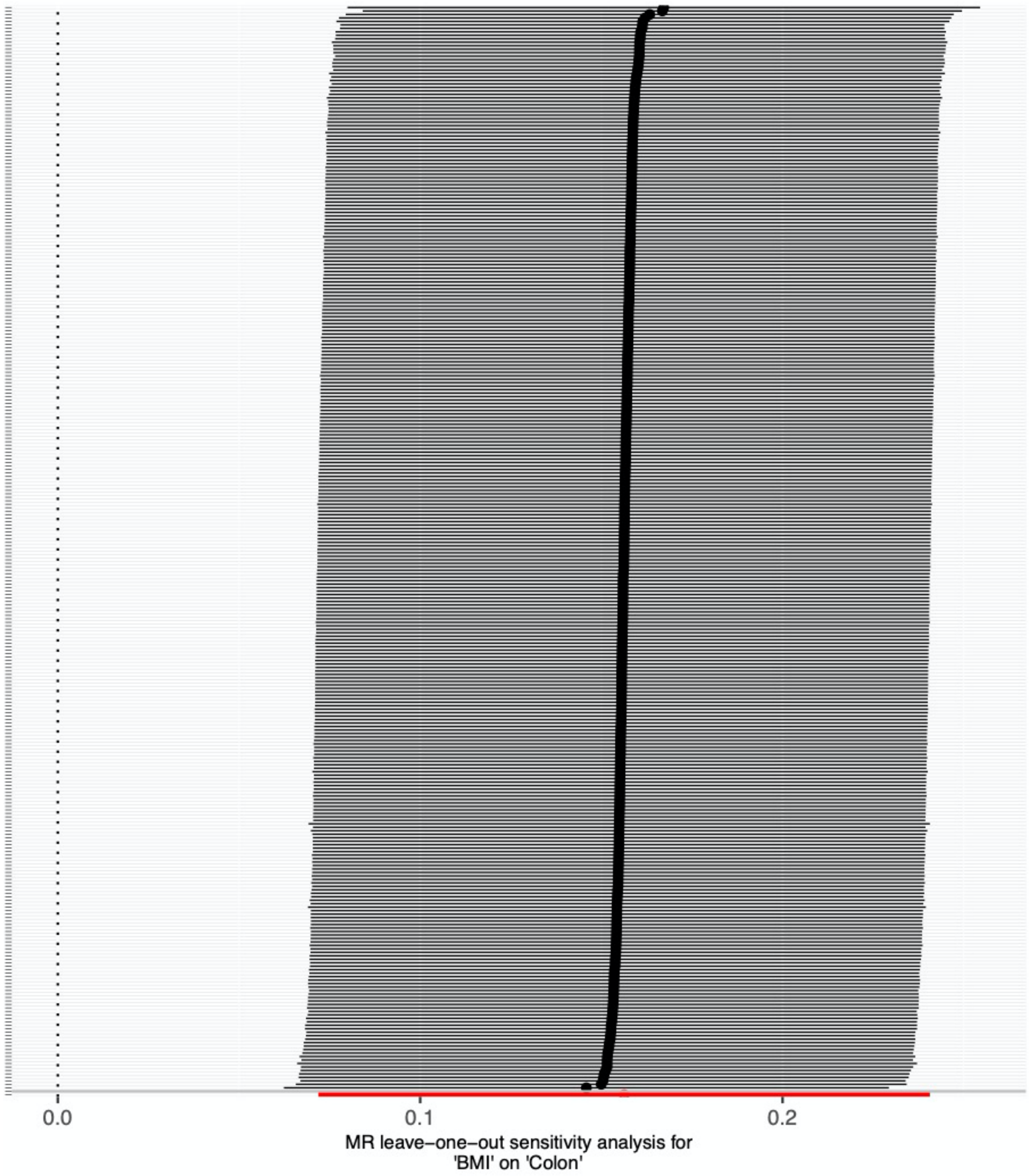

Figure S18. Leave-one-out plot showing the association between BMI and colon cancer, following SNP-by-SNP removal from the model

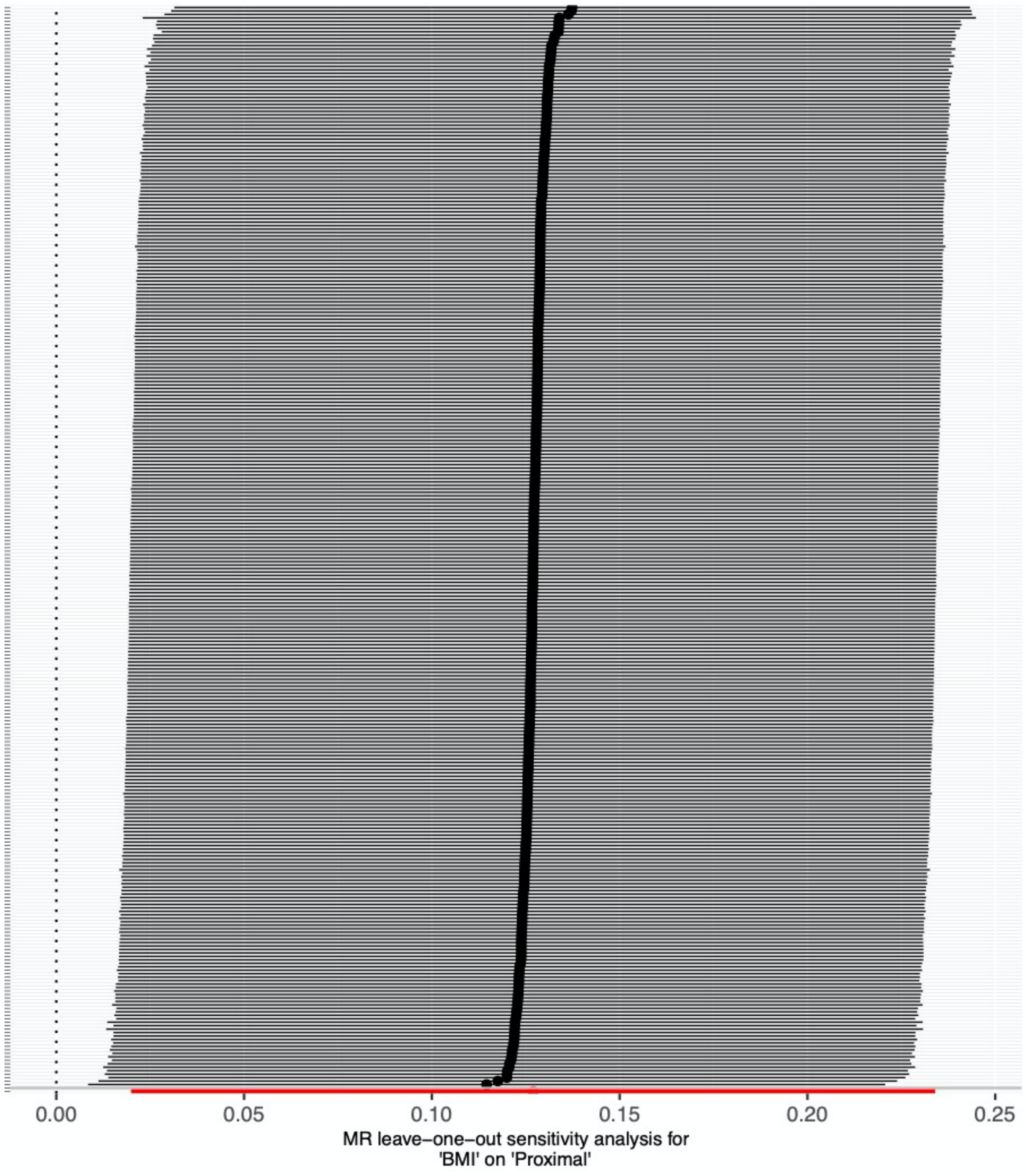

Figure S19. Leave-one-out plot showing the association between BMI and proximal colon cancer, following SNP-by-SNP removal from the model

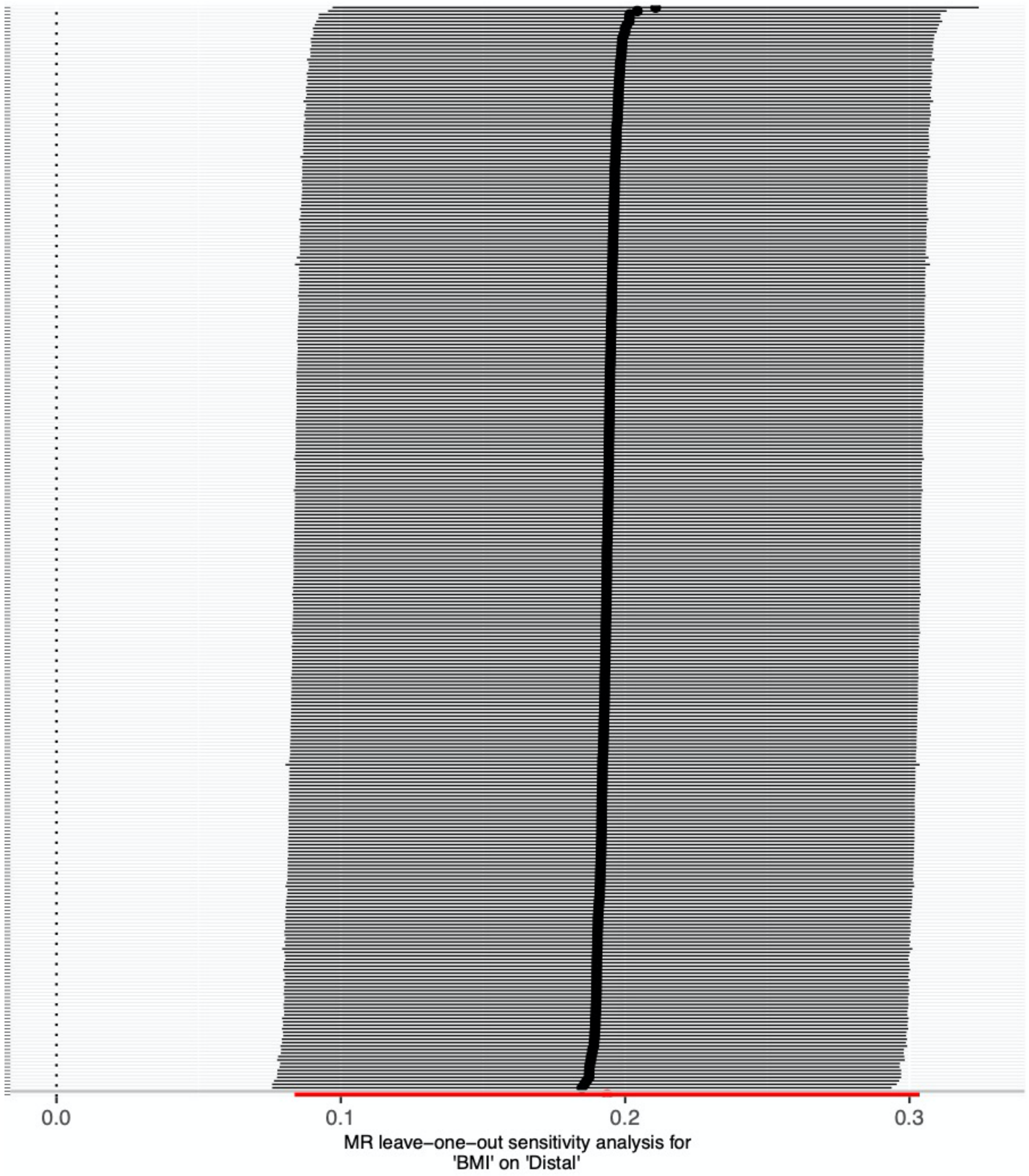

Figure S20. Leave-one-out plot showing the association between BMI and distal colon cancer, following SNP-by-SNP removal from the model

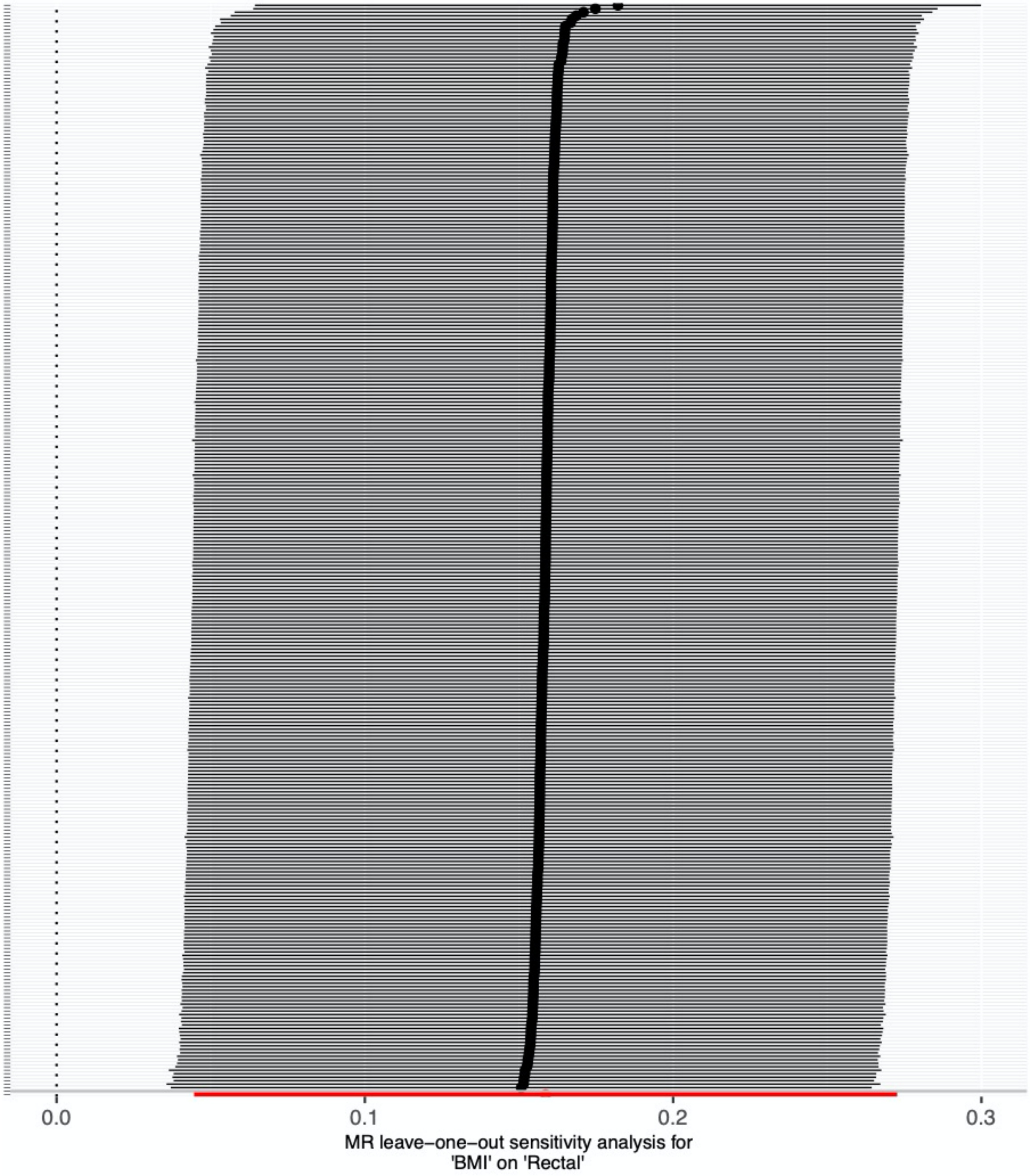

Figure S21. Leave-one-out plot showing the association between BMI and rectal cancer, following SNP-by-SNP removal from the model

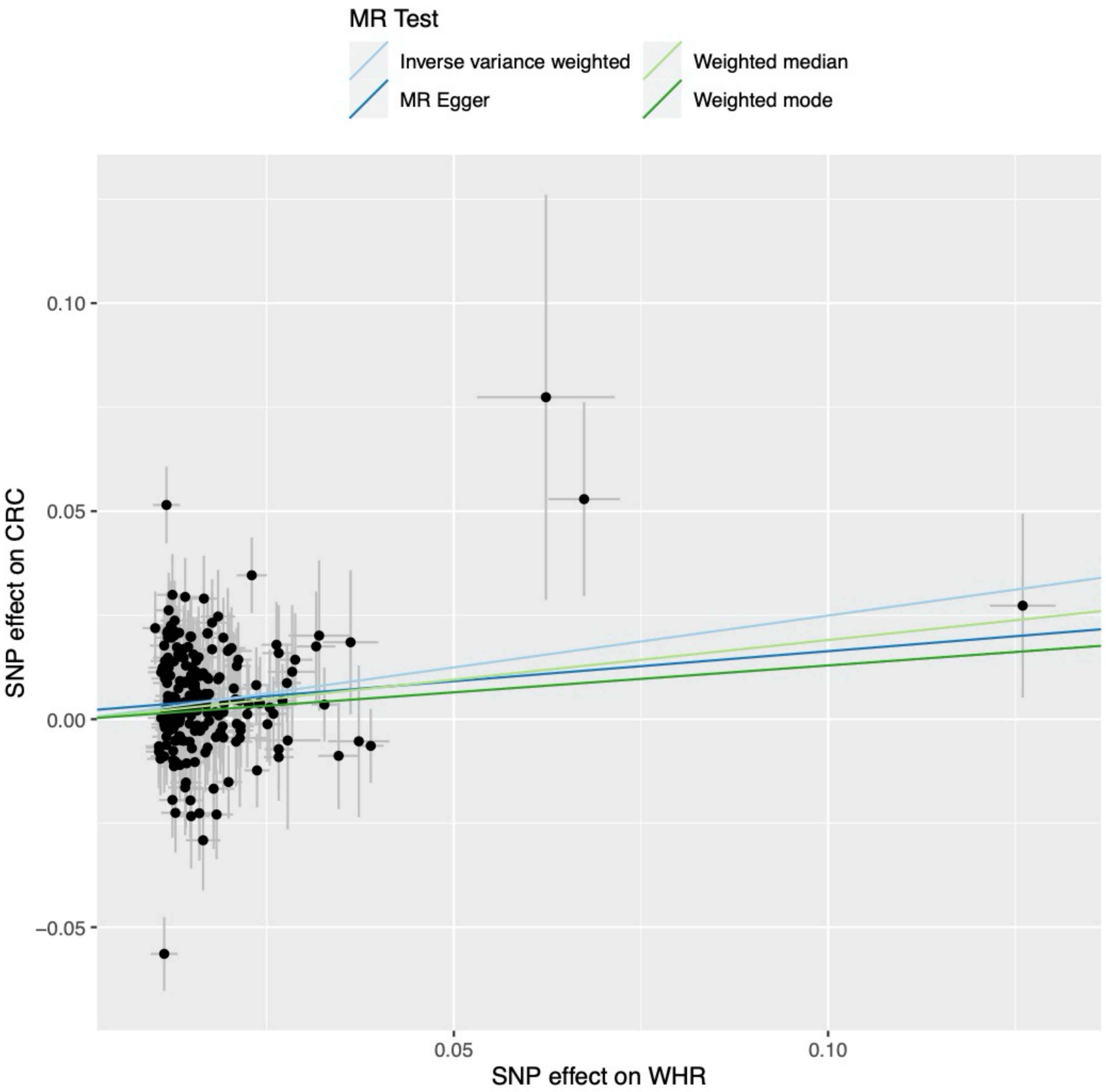

Figure S22. Scatter plot of SNP-WHR and SNP-CRC associations

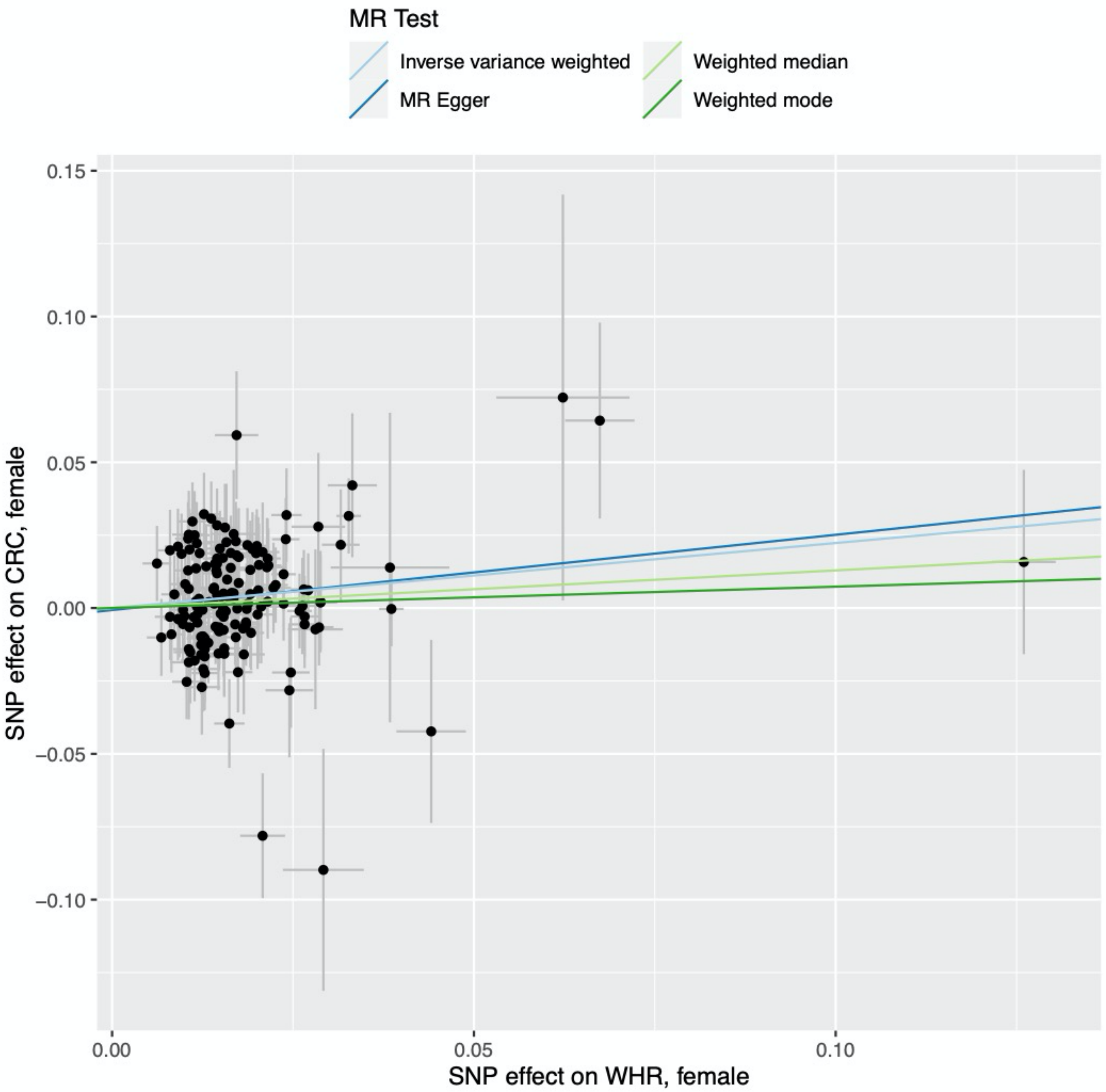

Figure S23. Scatter plot of SNP-WHR and SNP-CRC associations (female specific)

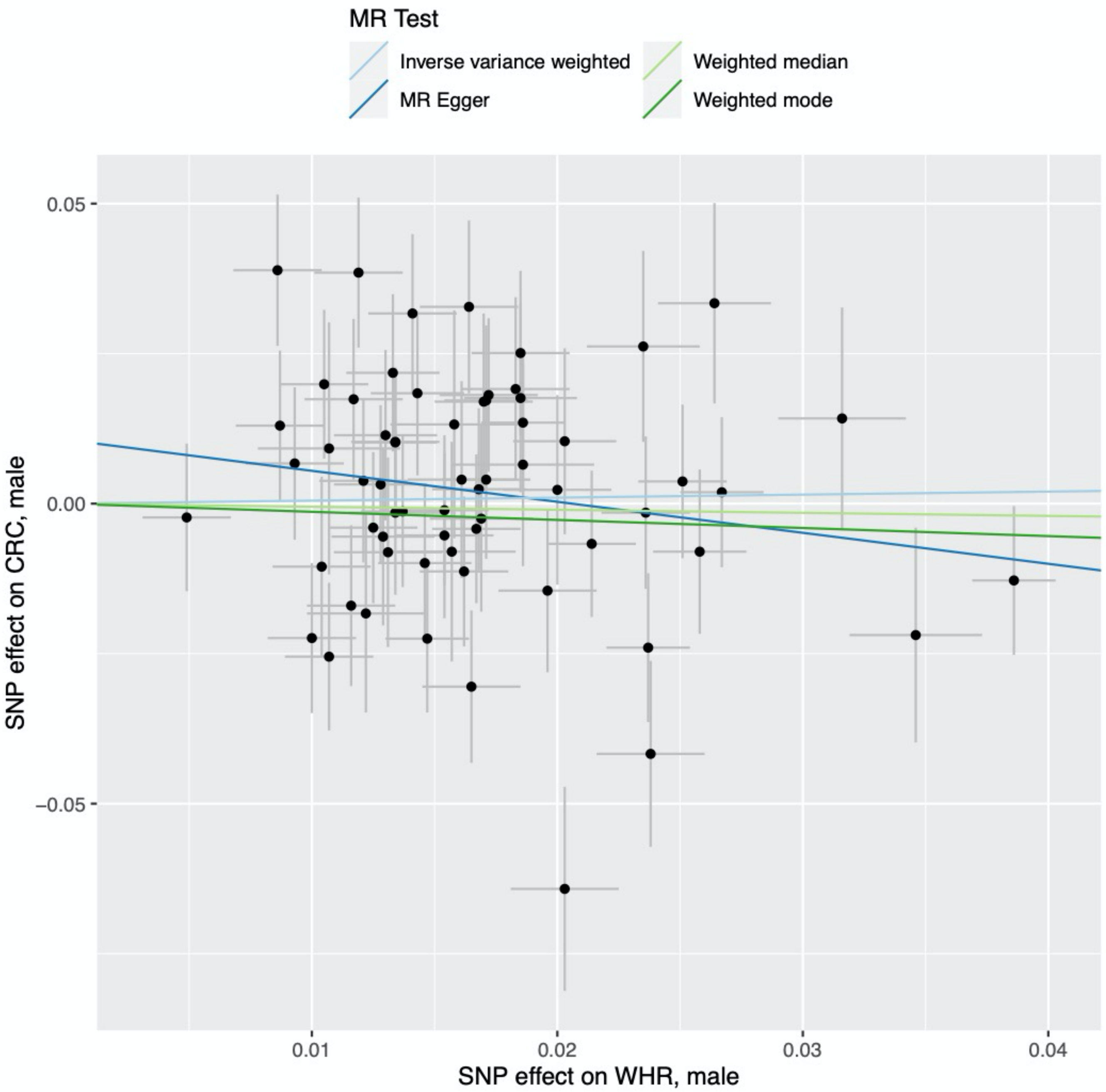

Figure S24. Scatter plot of SNP-WHR and SNP-CRC associations (male specific)

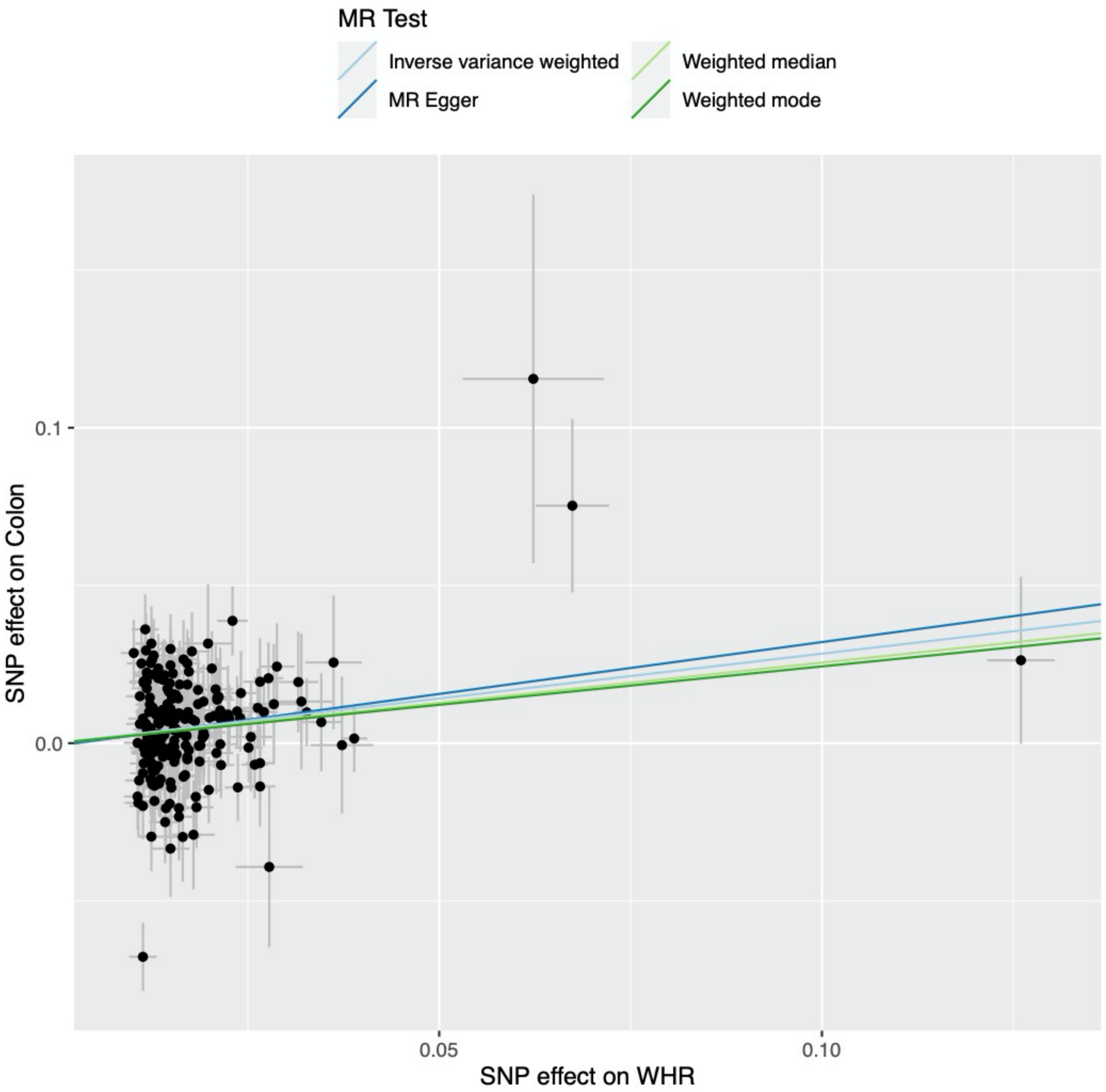

Figure S25. Scatter plot of SNP-WHR and SNP-colon cancer associations

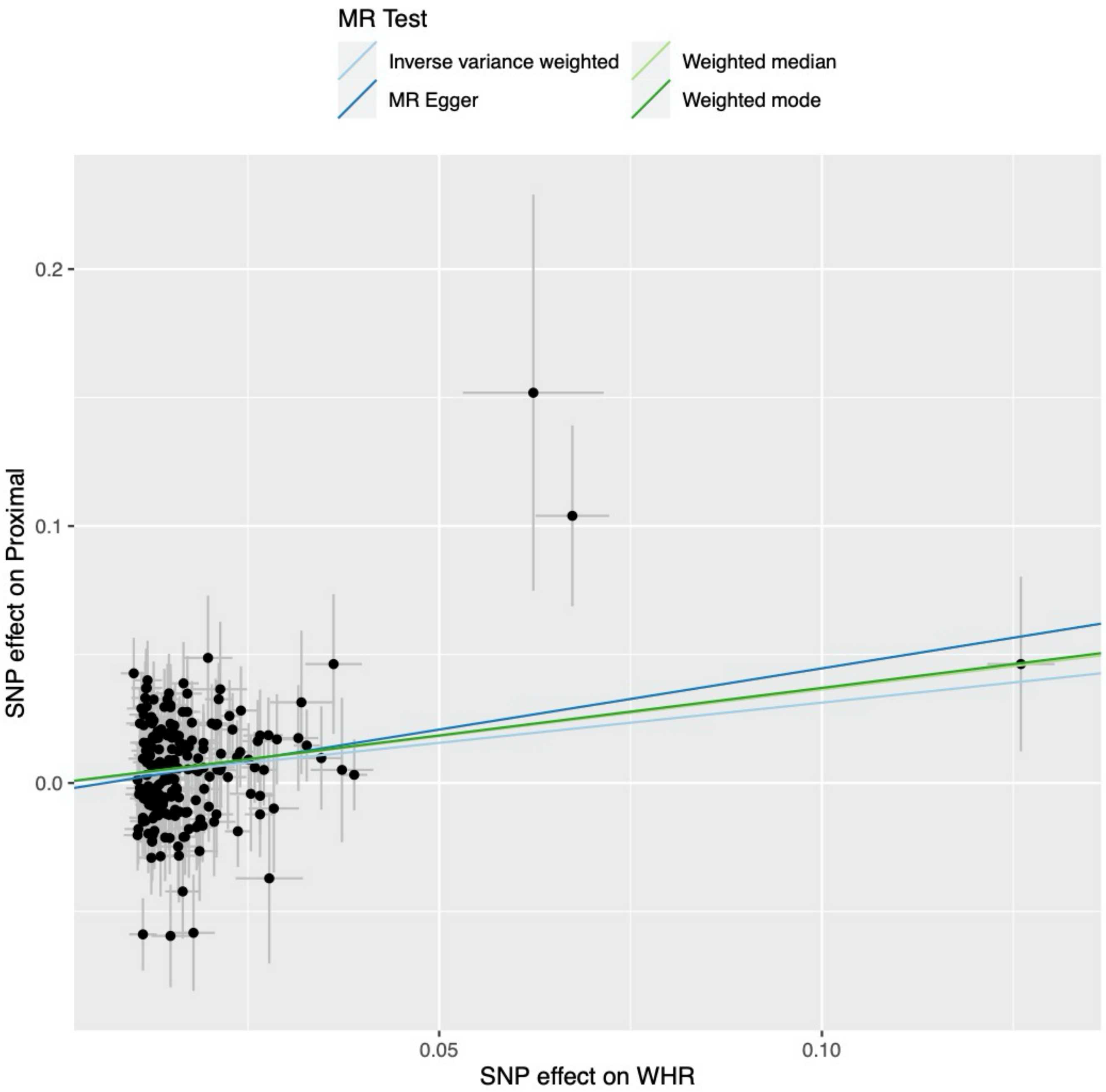

Figure S26. Scatter plot of SNP-WHR and SNP-proximal colon cancer associations

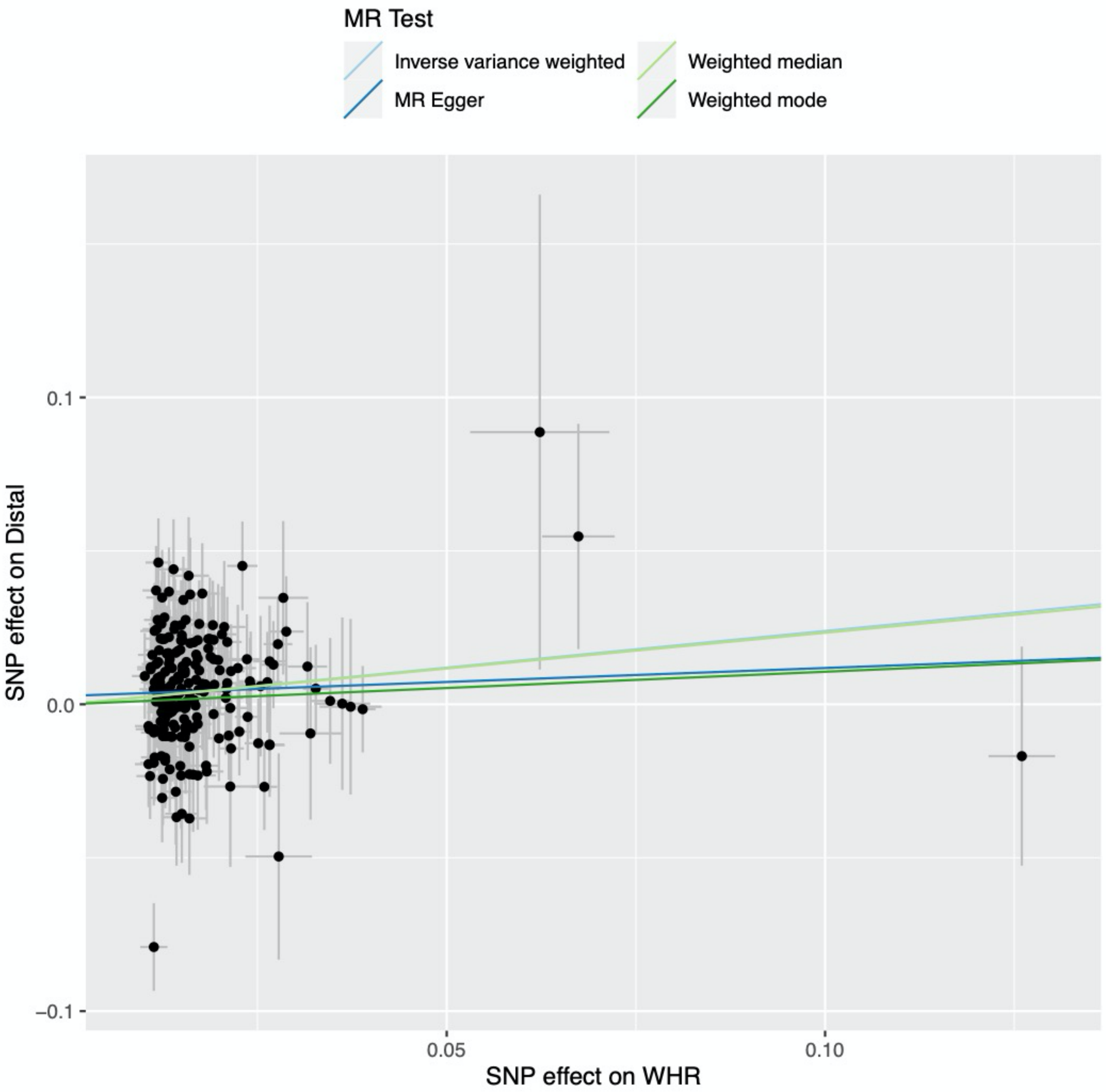

Figure S27. Scatter plot of SNP-WHR and SNP-distal colon cancer associations

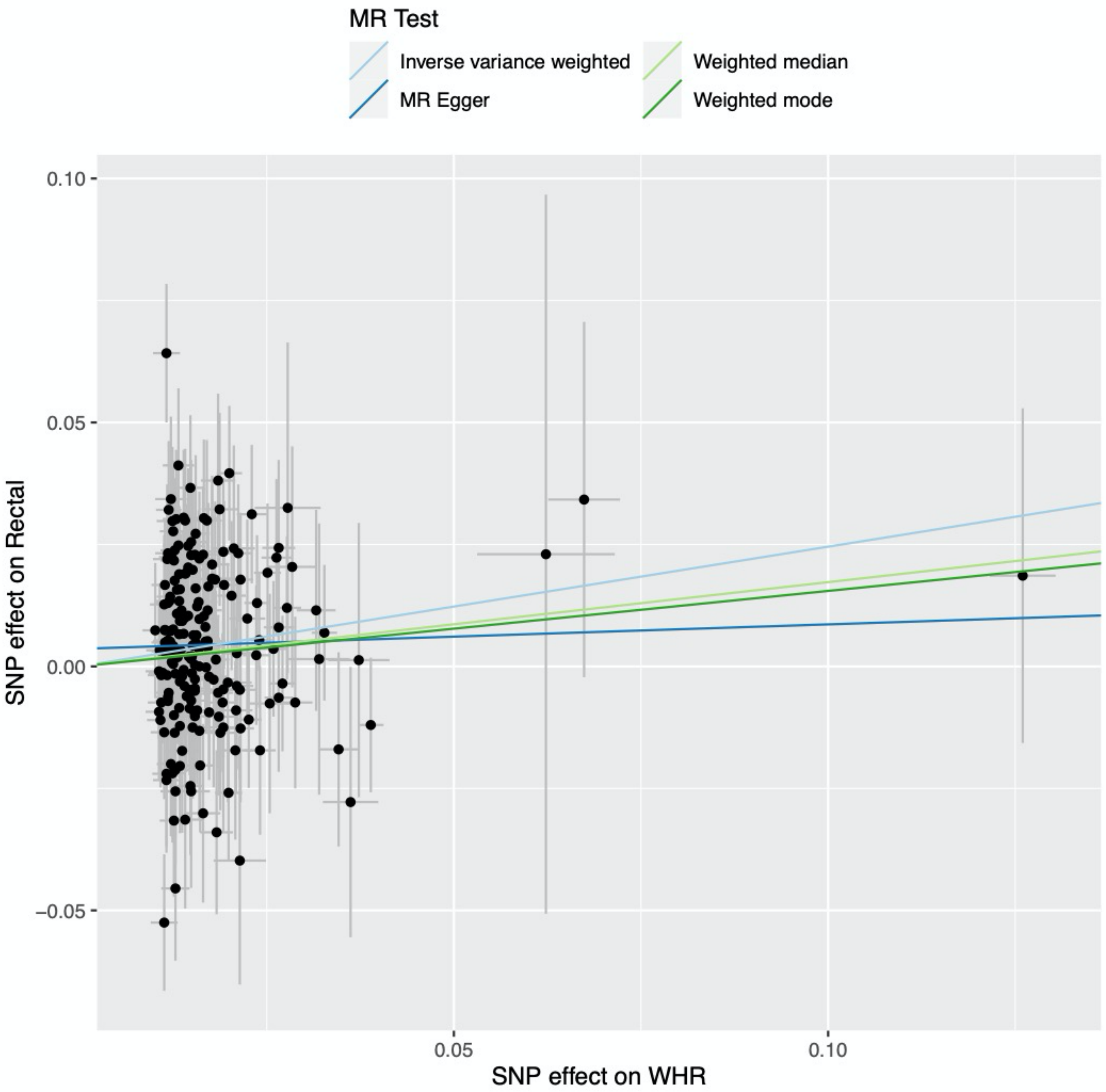

Figure S28. Scatter plot of SNP-WHR and SNP-rectal cancer associations

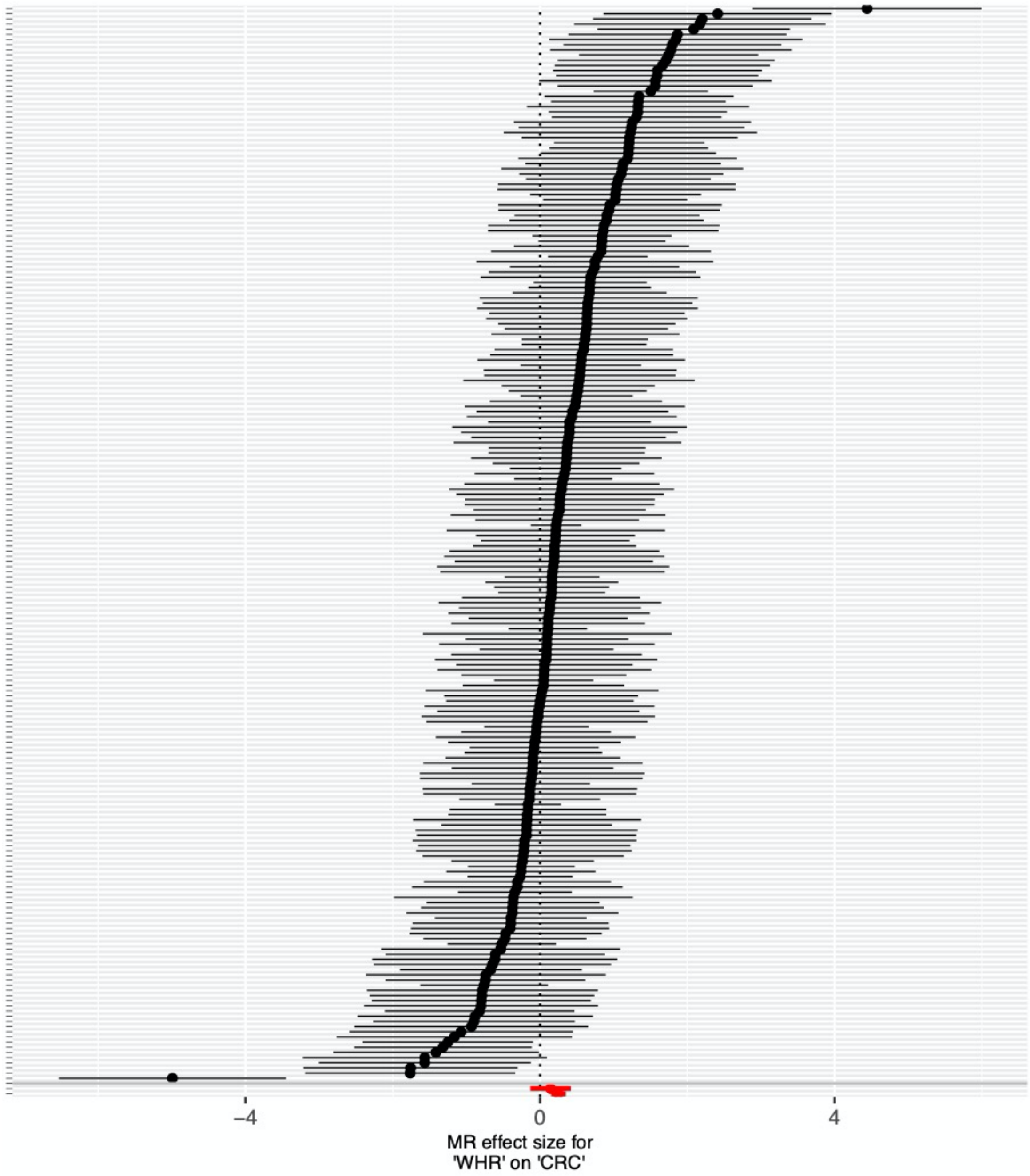

Figure S29. Forest plot showing individual SNP (black) and combined MR estimates (red; Egger and IVW) for the effect of WHR on CRC

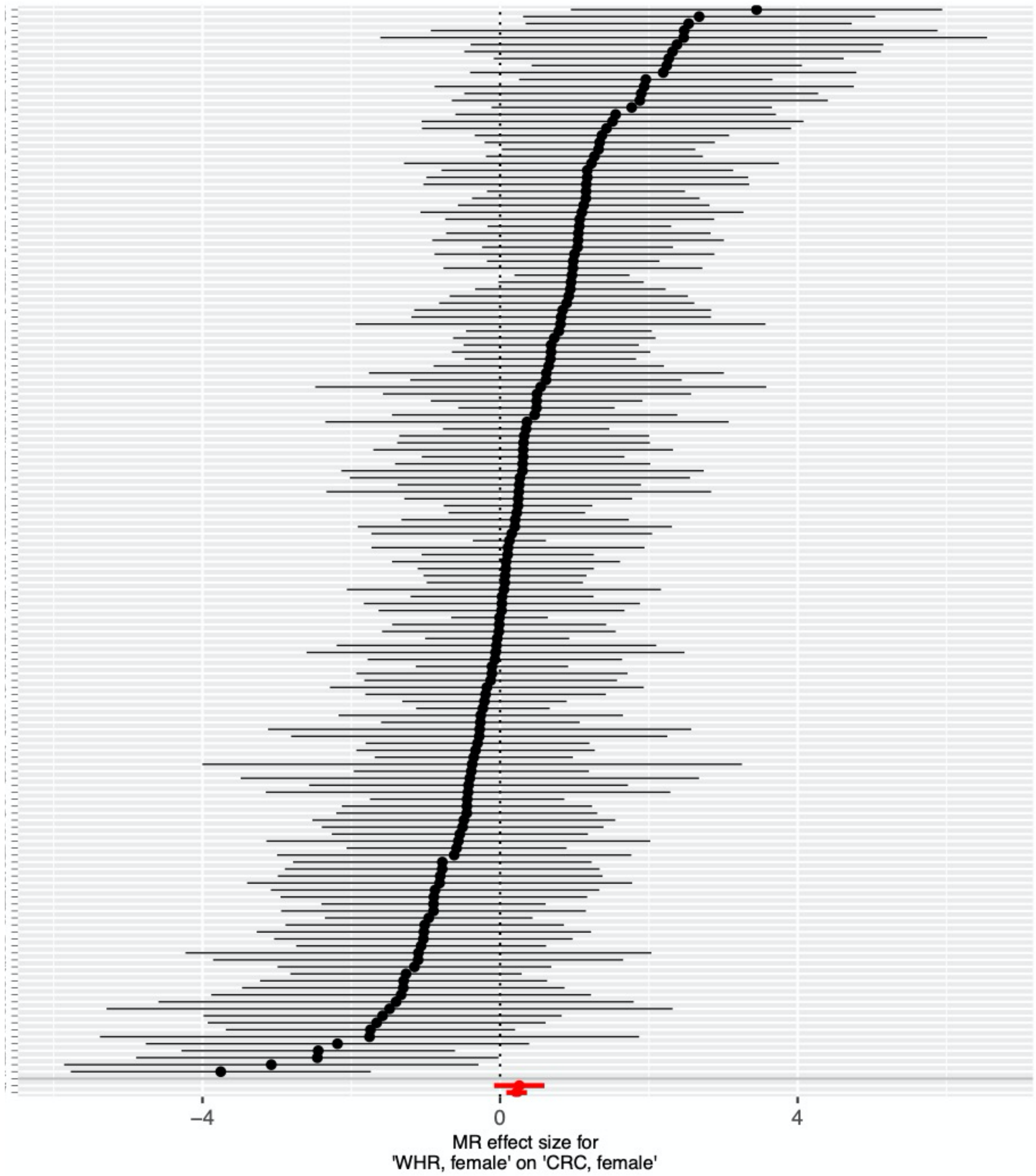

Figure S30. Forest plot showing individual SNP (black) and combined MR estimates (red; Egger and IVW) for the effect of WHR on CRC (female specific)

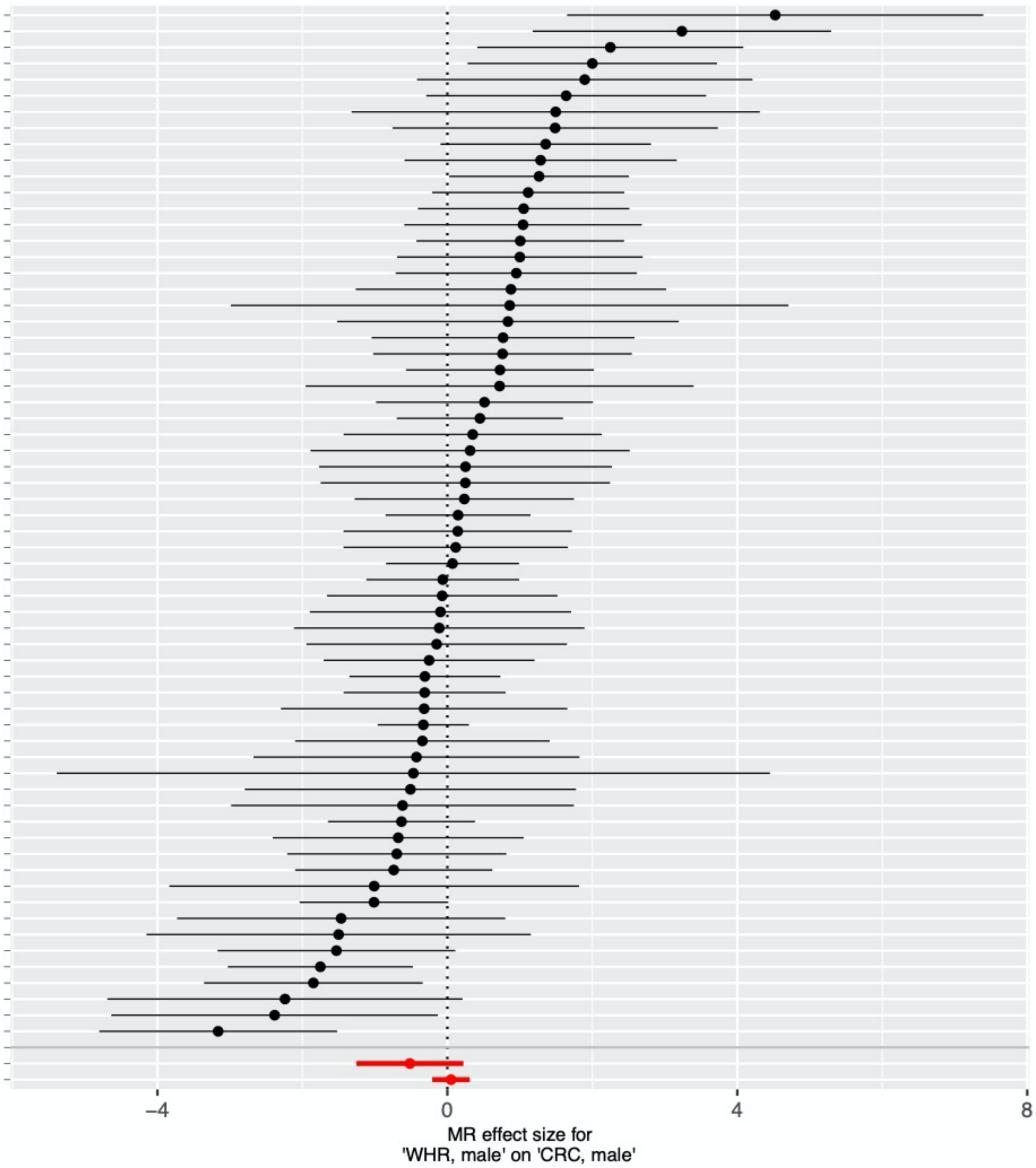

Figure S31. Forest plot showing individual SNP (black) and combined MR estimates (red; Egger and IVW) for the effect of WHR on CRC (male specific)

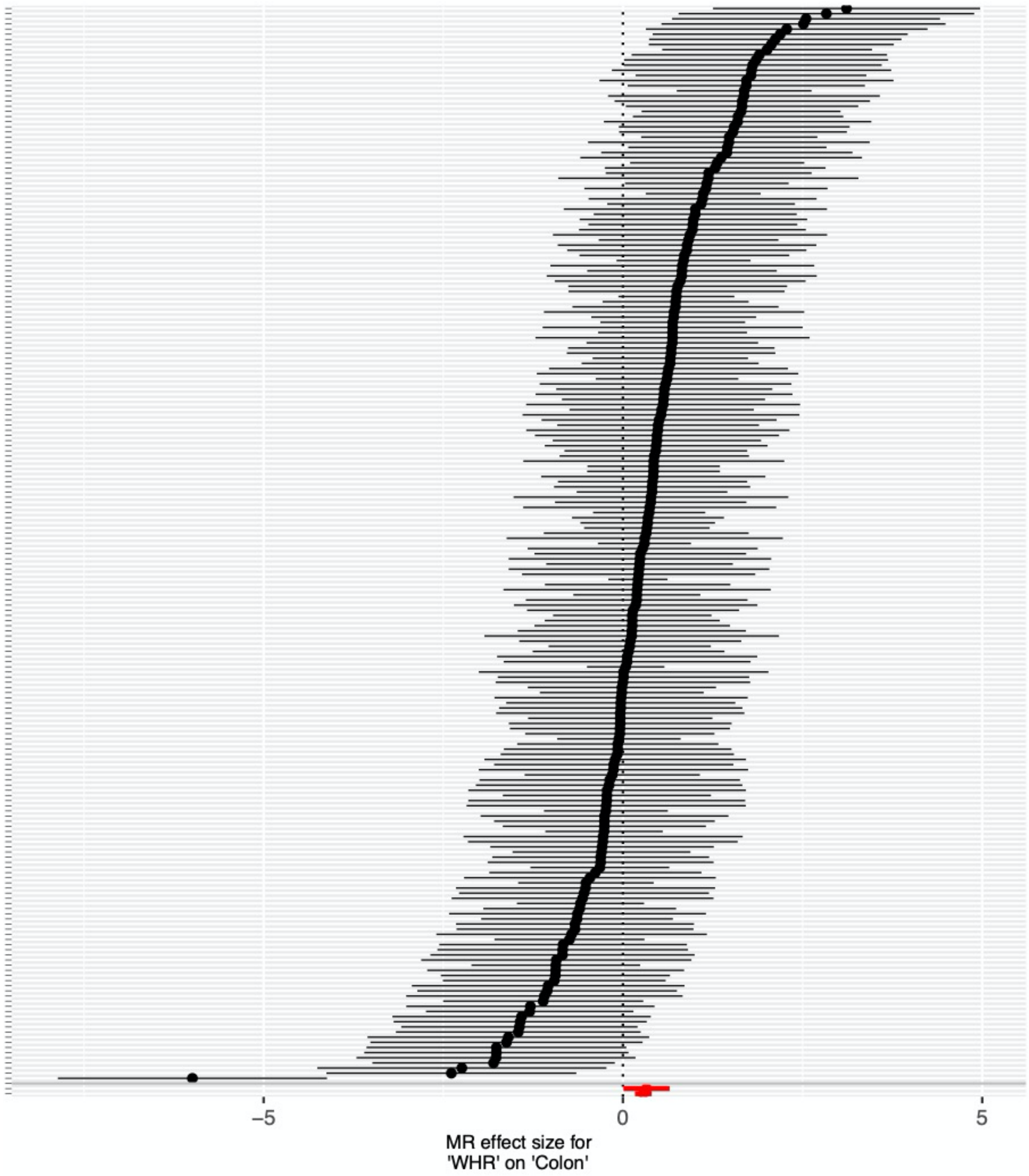

Figure S32. Forest plot showing individual SNP (black) and combined MR estimates (red; Egger and IVW) for the effect of WHR on colon cancer

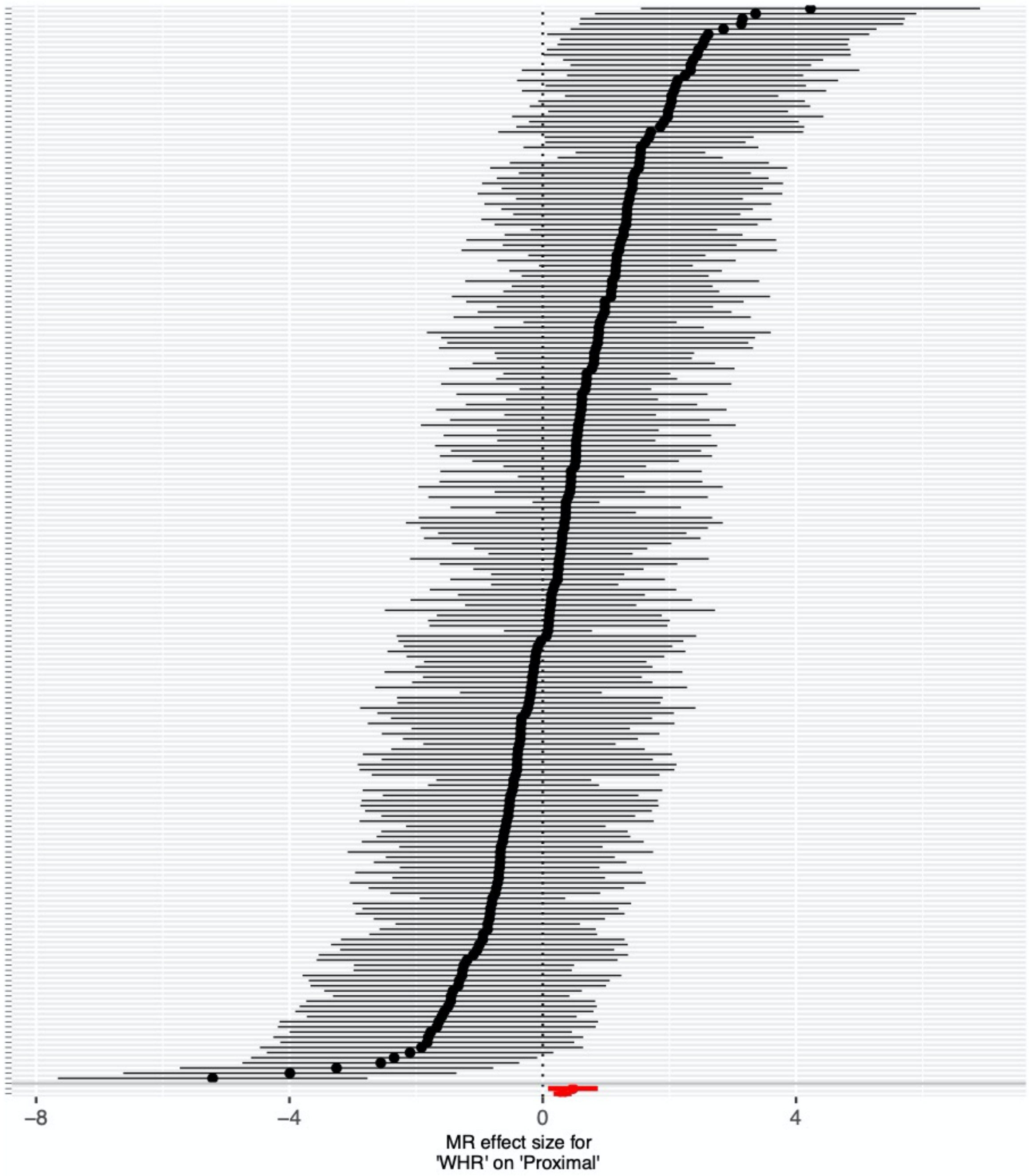

Figure S33. Forest plot showing individual SNP (black) and combined MR estimates (red; Egger and IVW) for the effect of WHR on proximal colon cancer

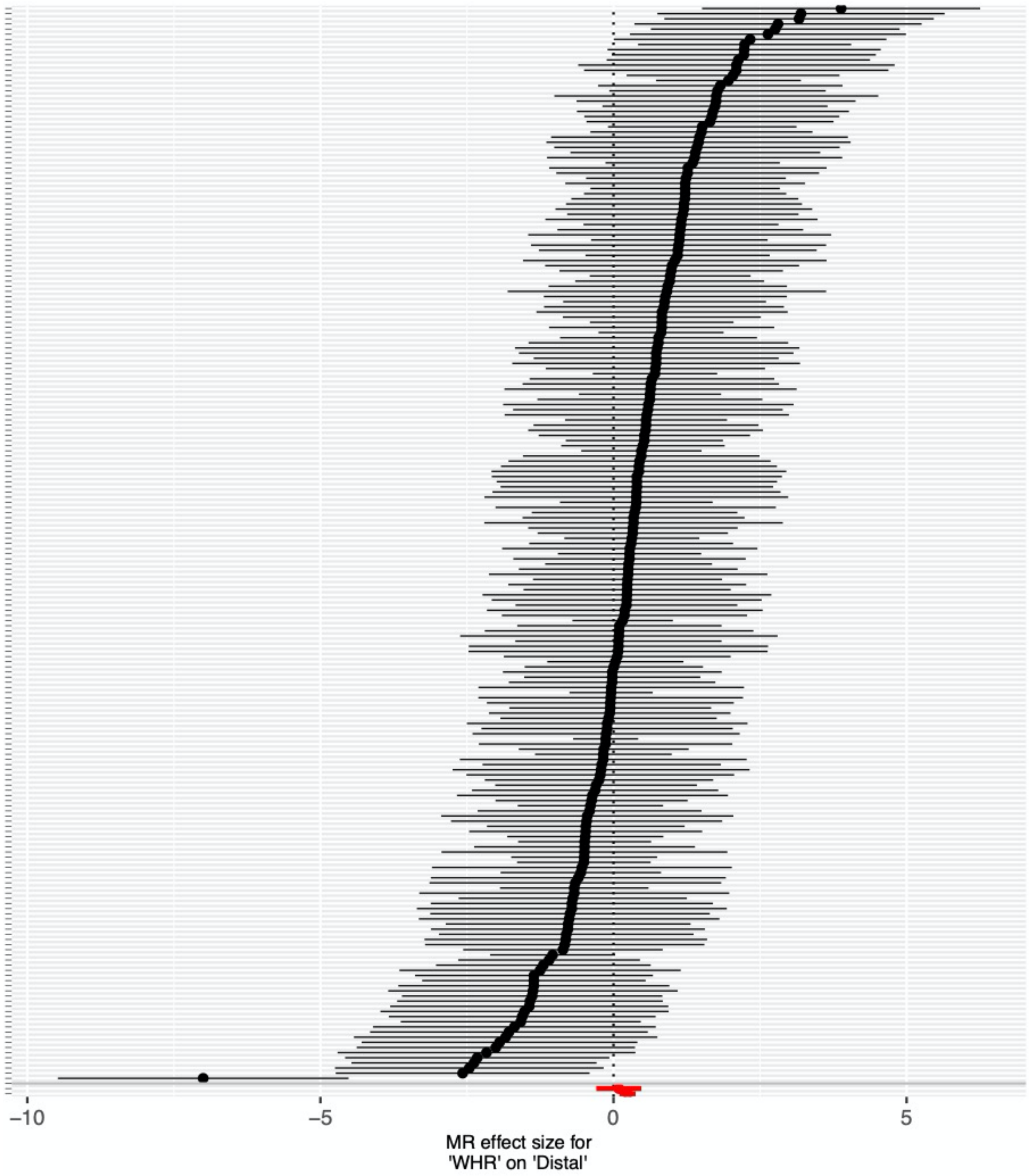

Figure S34. Forest plot showing individual SNP (black) and combined MR estimates (red; Egger and IVW) for the effect of WHR on distal colon cancer

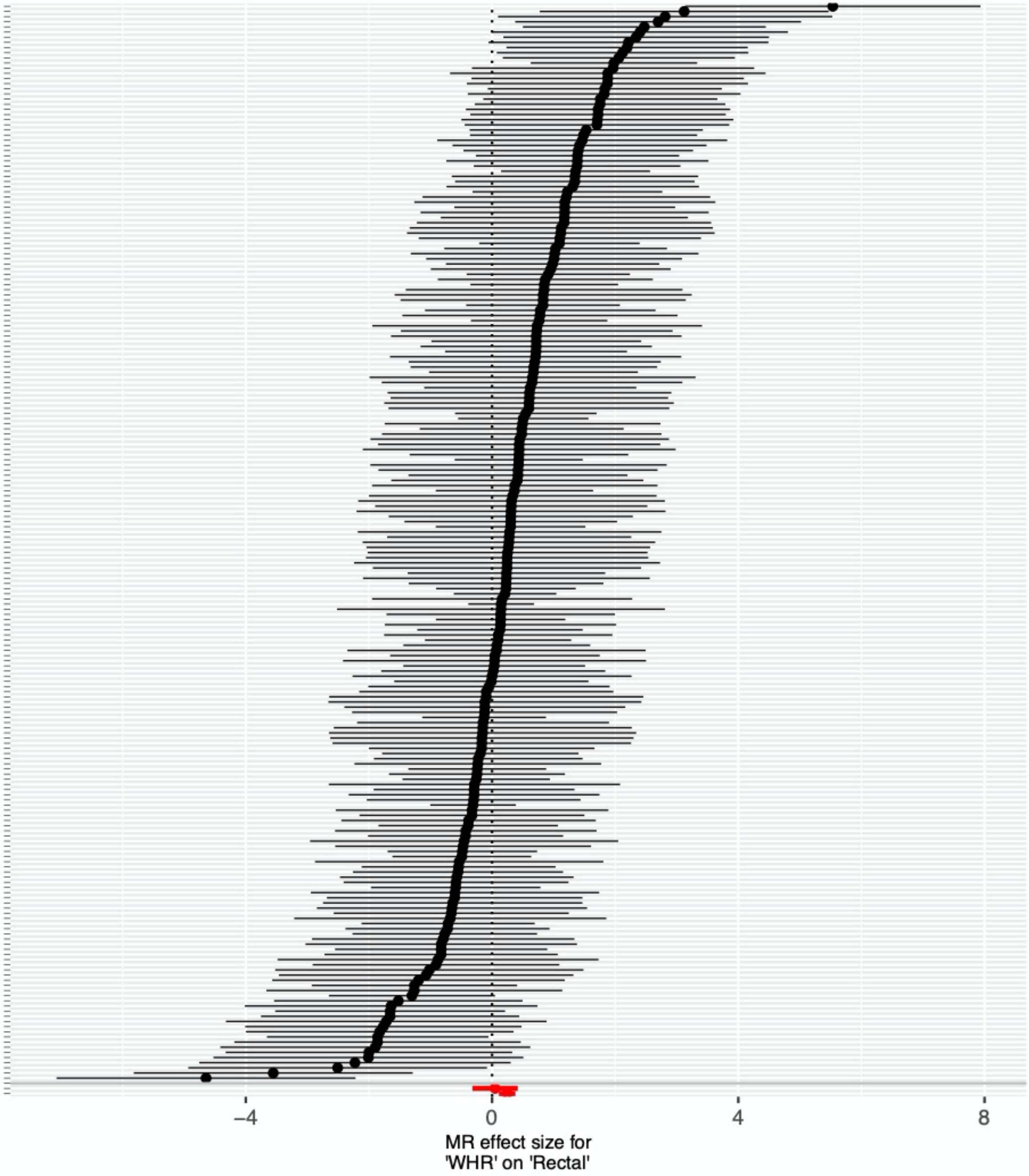

Figure S35. Forest plot showing individual SNP (black) and combined MR estimates (red; Egger and IVW) for the effect of WHR on rectal cancer

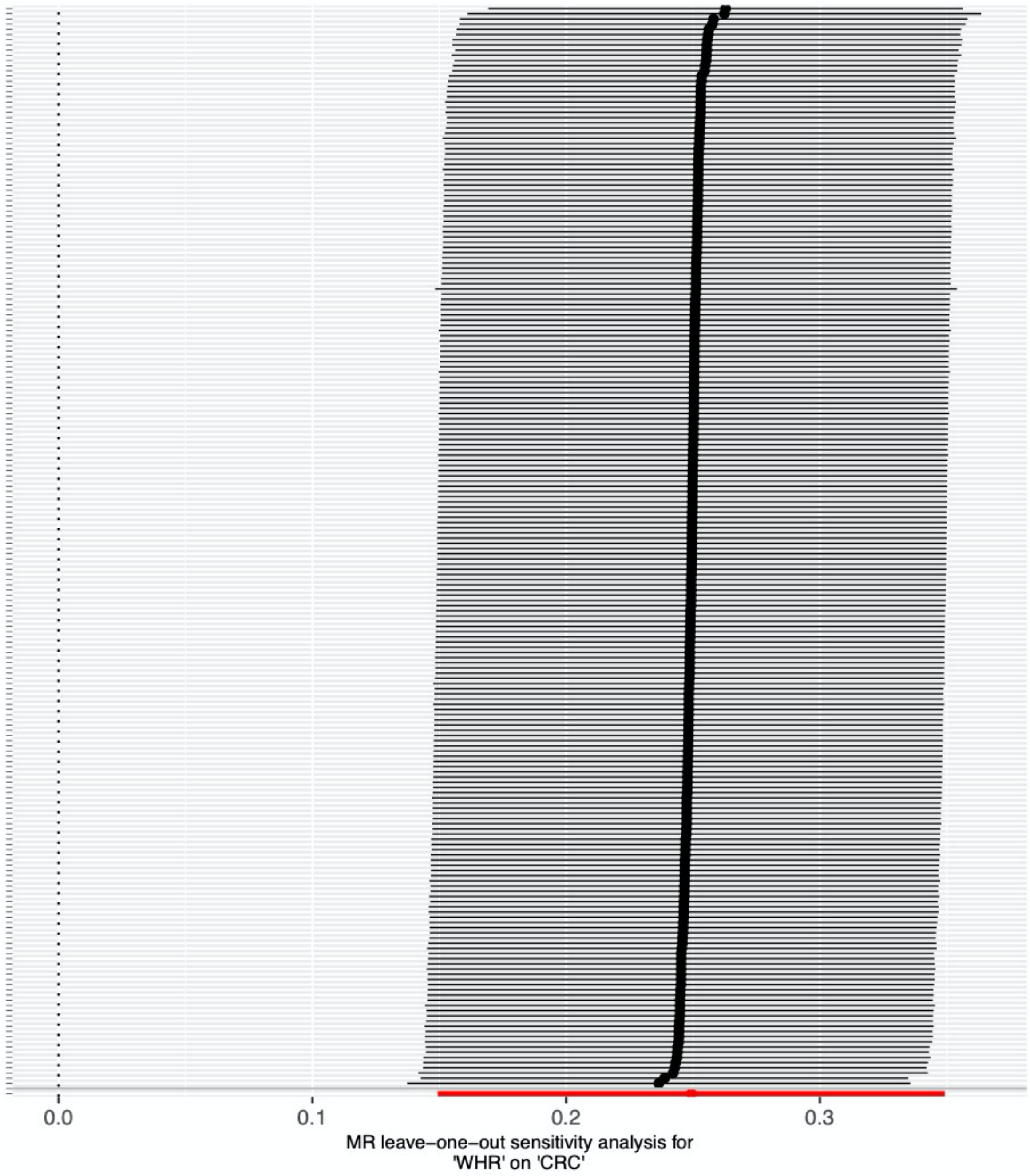

Figure S36. Leave-one-out plot showing the association between WHR and CRC, following SNP-by-SNP removal from the model

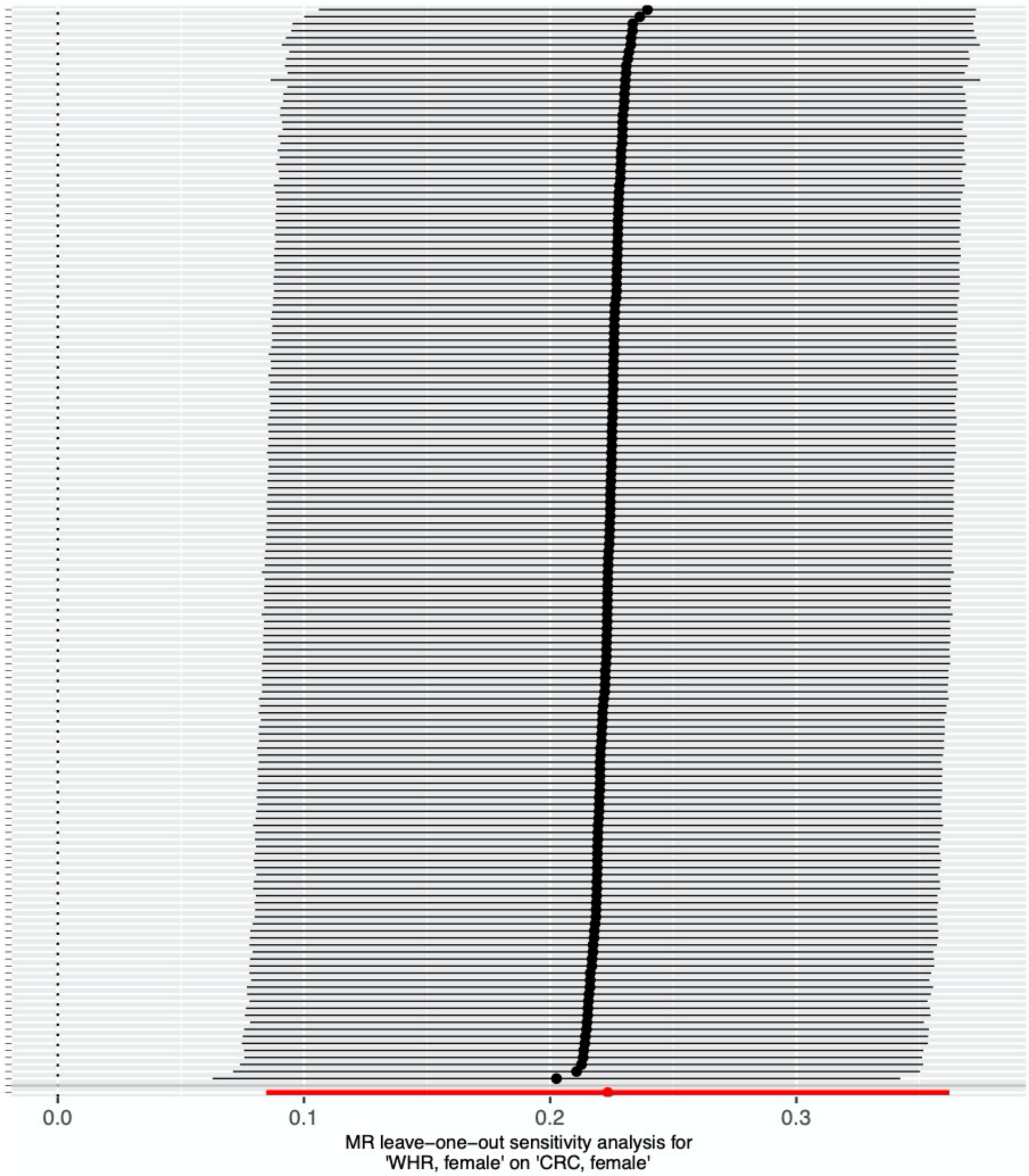

Figure S37. Leave-one-out plot showing the association between WHR and CRC, following SNP-by-SNP removal from the model (female specific)

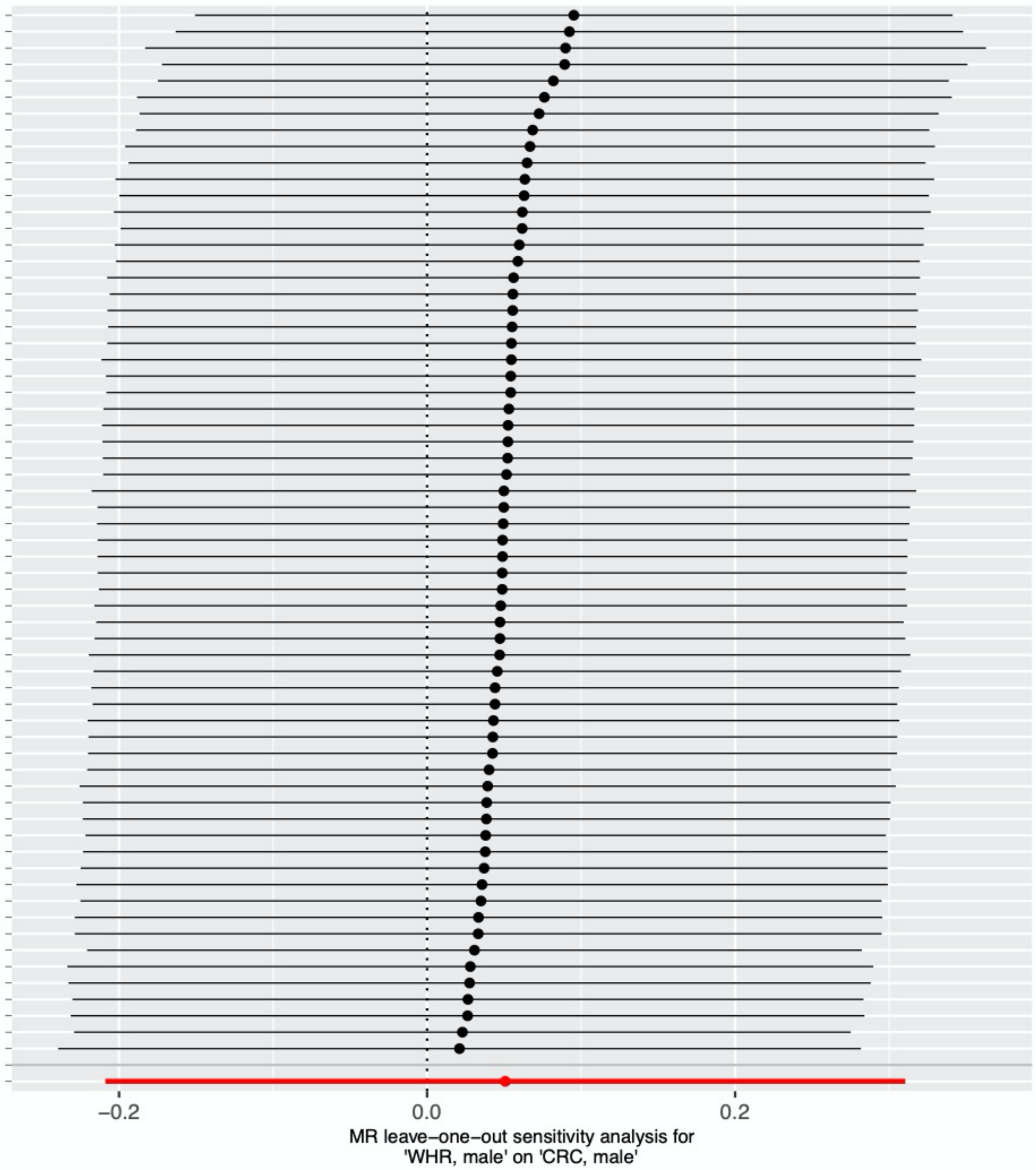

Figure S38. Leave-one-out plot showing the association between WHR and CRC, following SNP-by-SNP removal from the model (male specific)

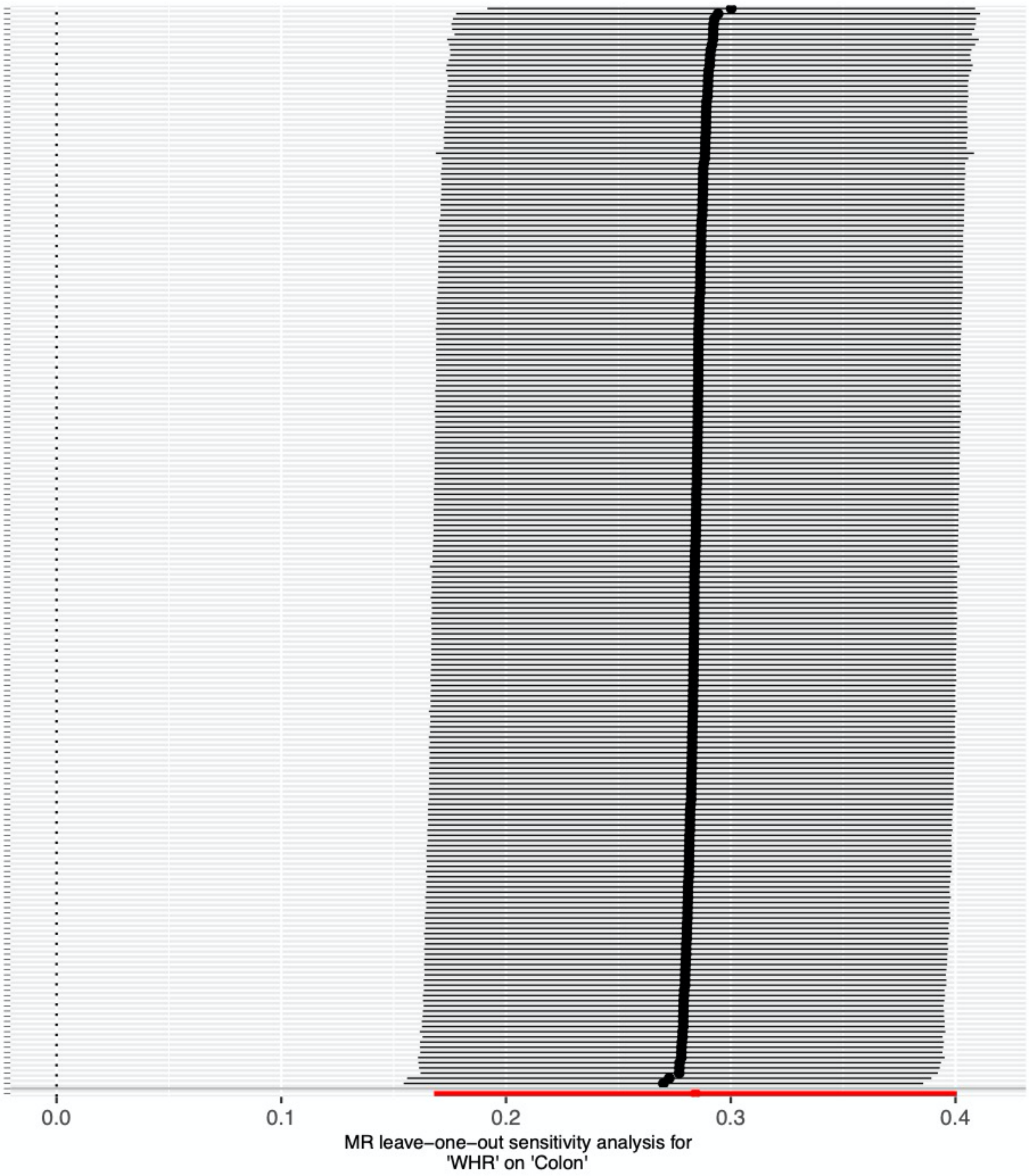

Figure S39. Leave-one-out plot showing the association between WHR and colon cancer, following SNP-by-SNP removal from the model

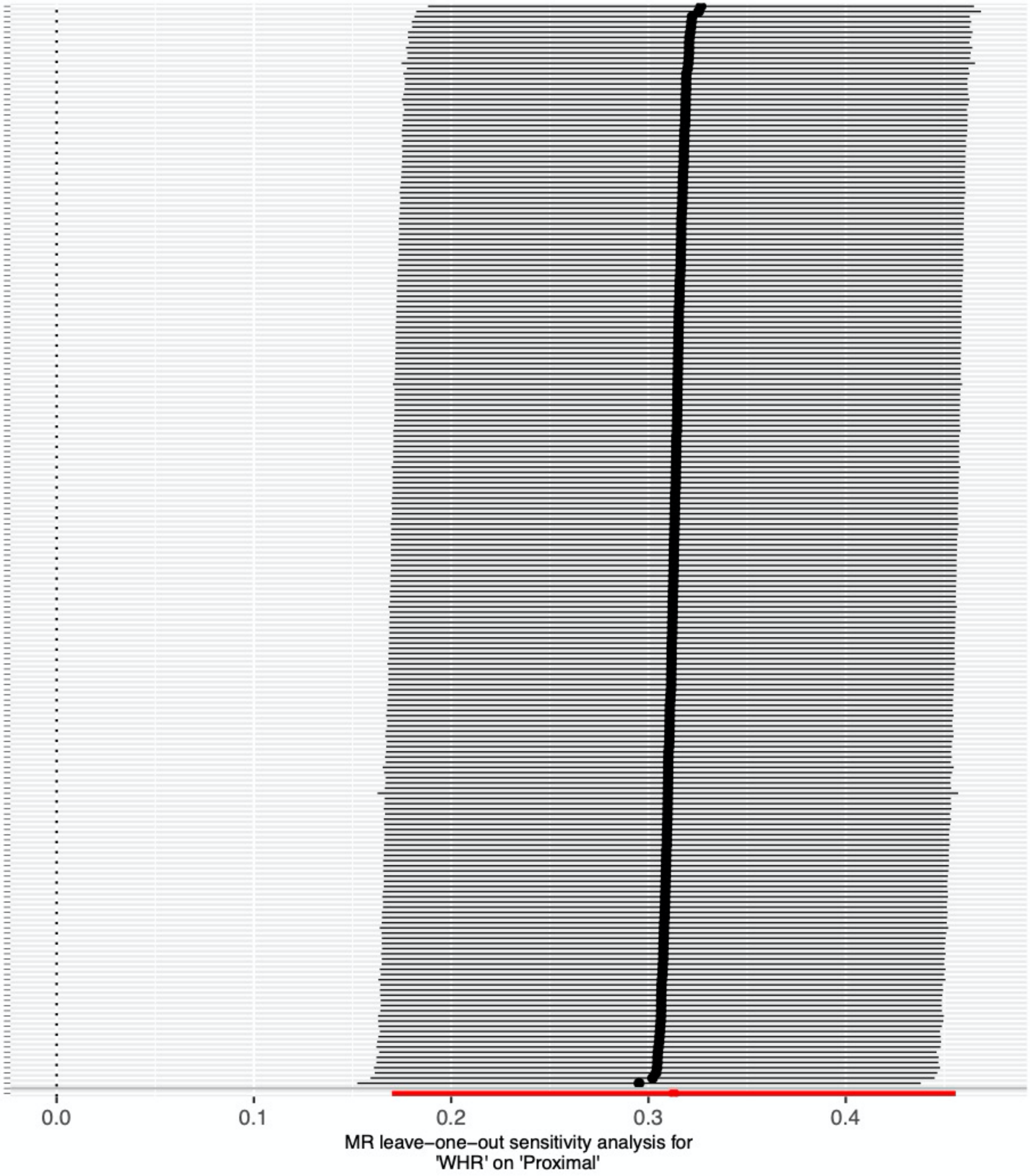

Figure S40. Leave-one-out plot showing the association between WHR and proximal colon cancer, following SNP-by-SNP removal from the model

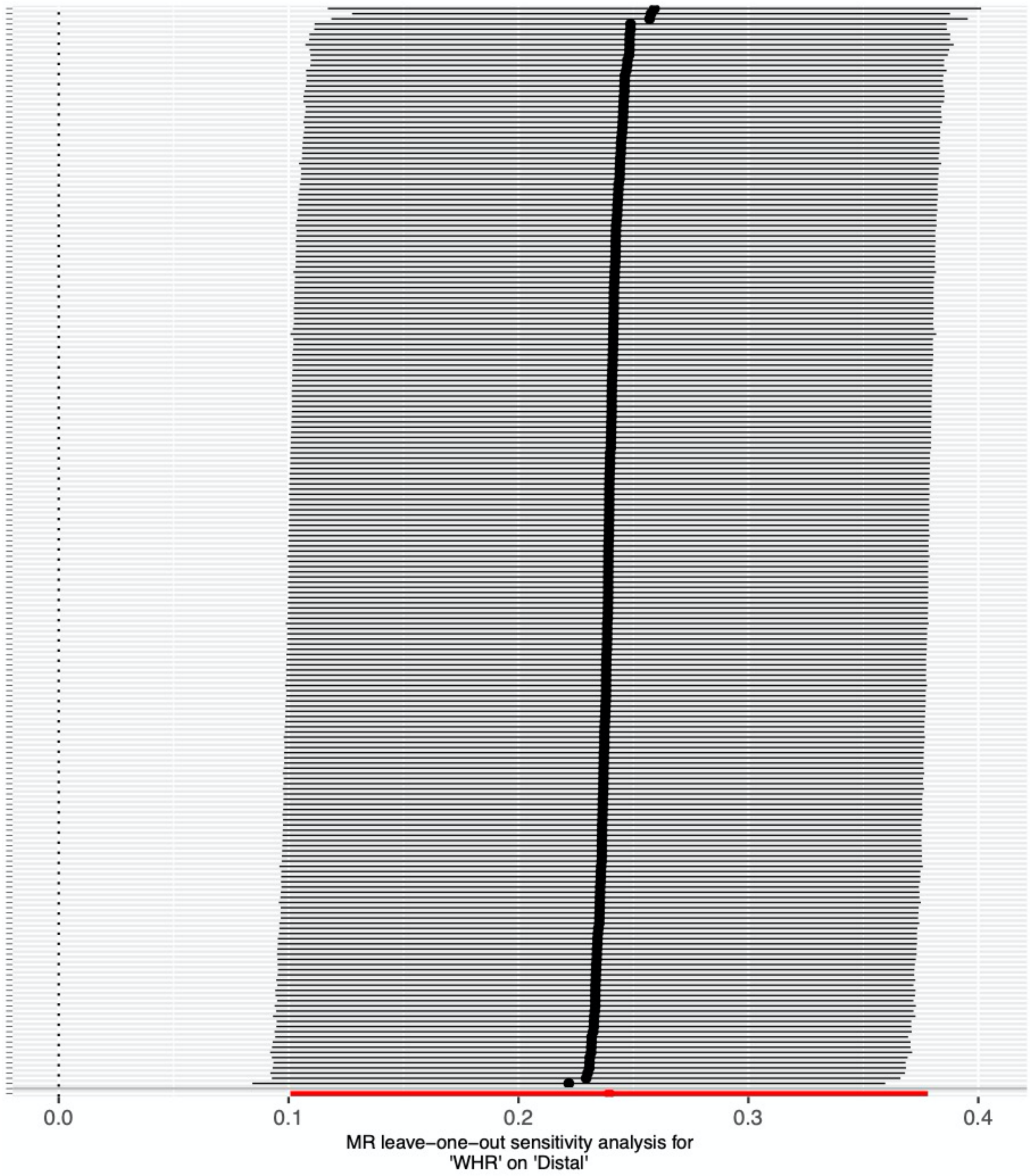

Figure S41. Leave-one-out plot showing the association between WHR and distal colon cancer, following SNP-by-SNP removal from the model

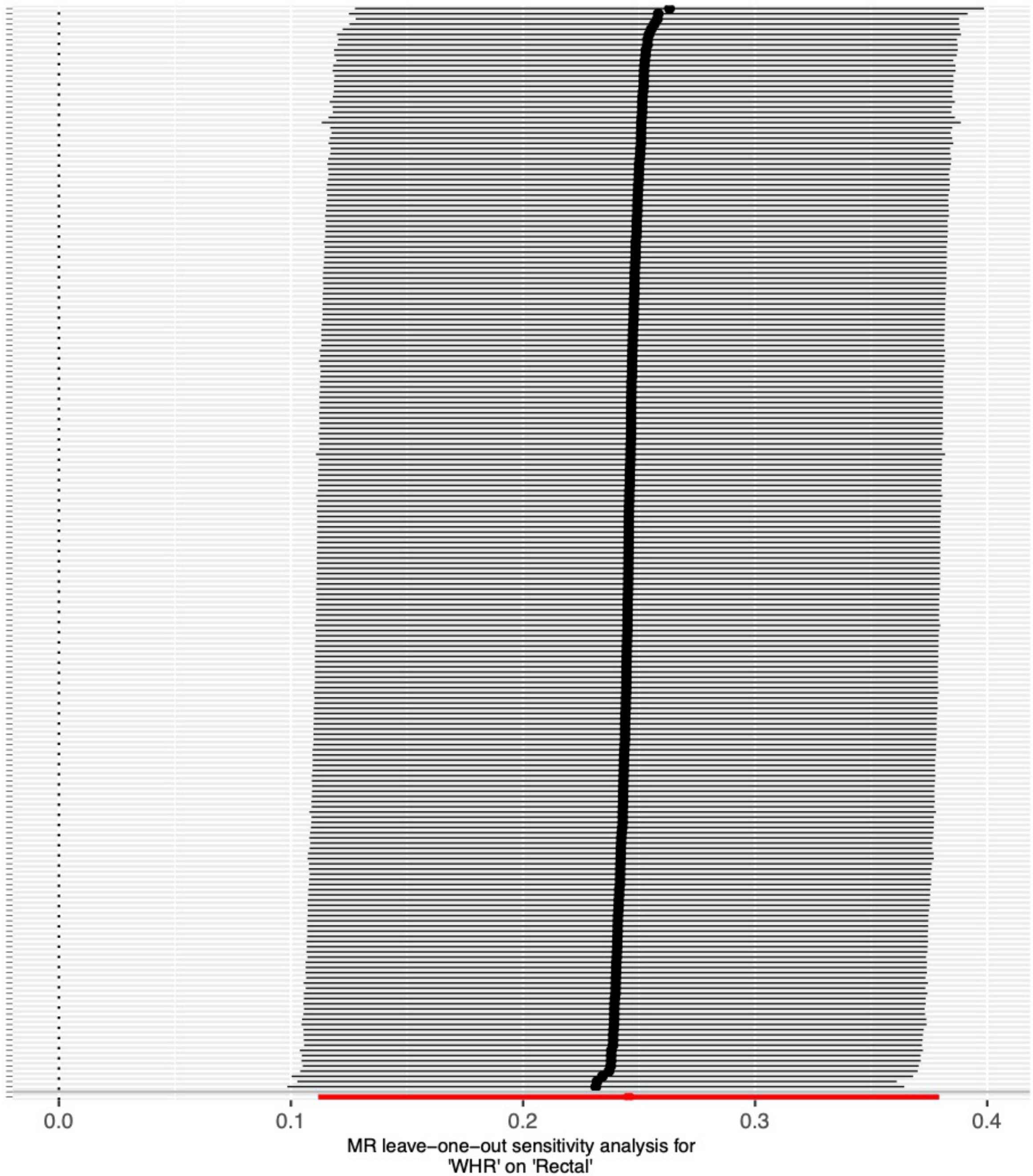

Figure S42. Leave-one-out plot showing the association between WHR and rectal cancer, following SNP-by-SNP removal from the model

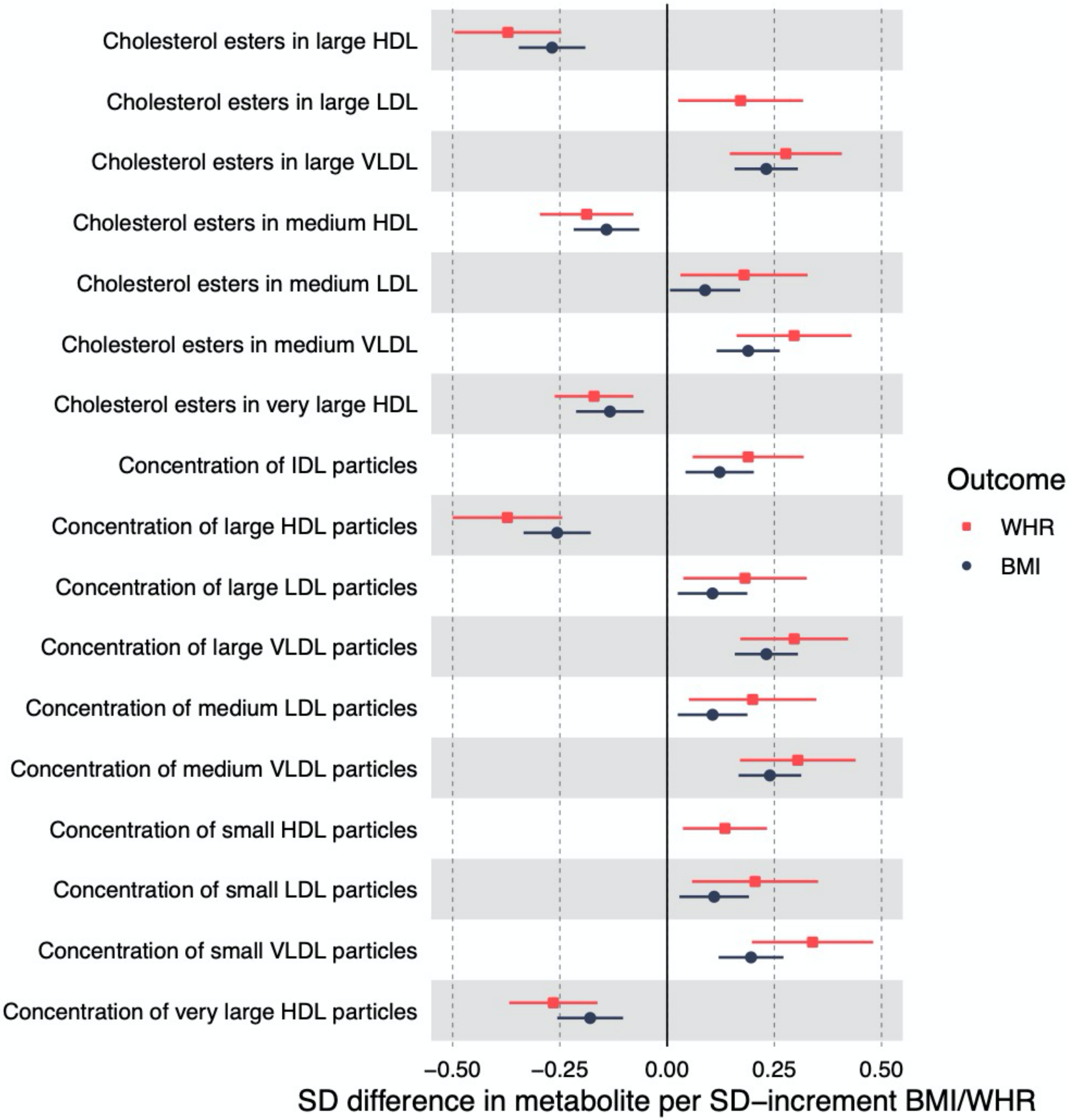

Figure S43. Effects of BMI and WHR on circulating metabolite levels (NMR-detected metabolites, 1 of 5), based on two-sample MR (IVW models) in summary GWAS consortia data.

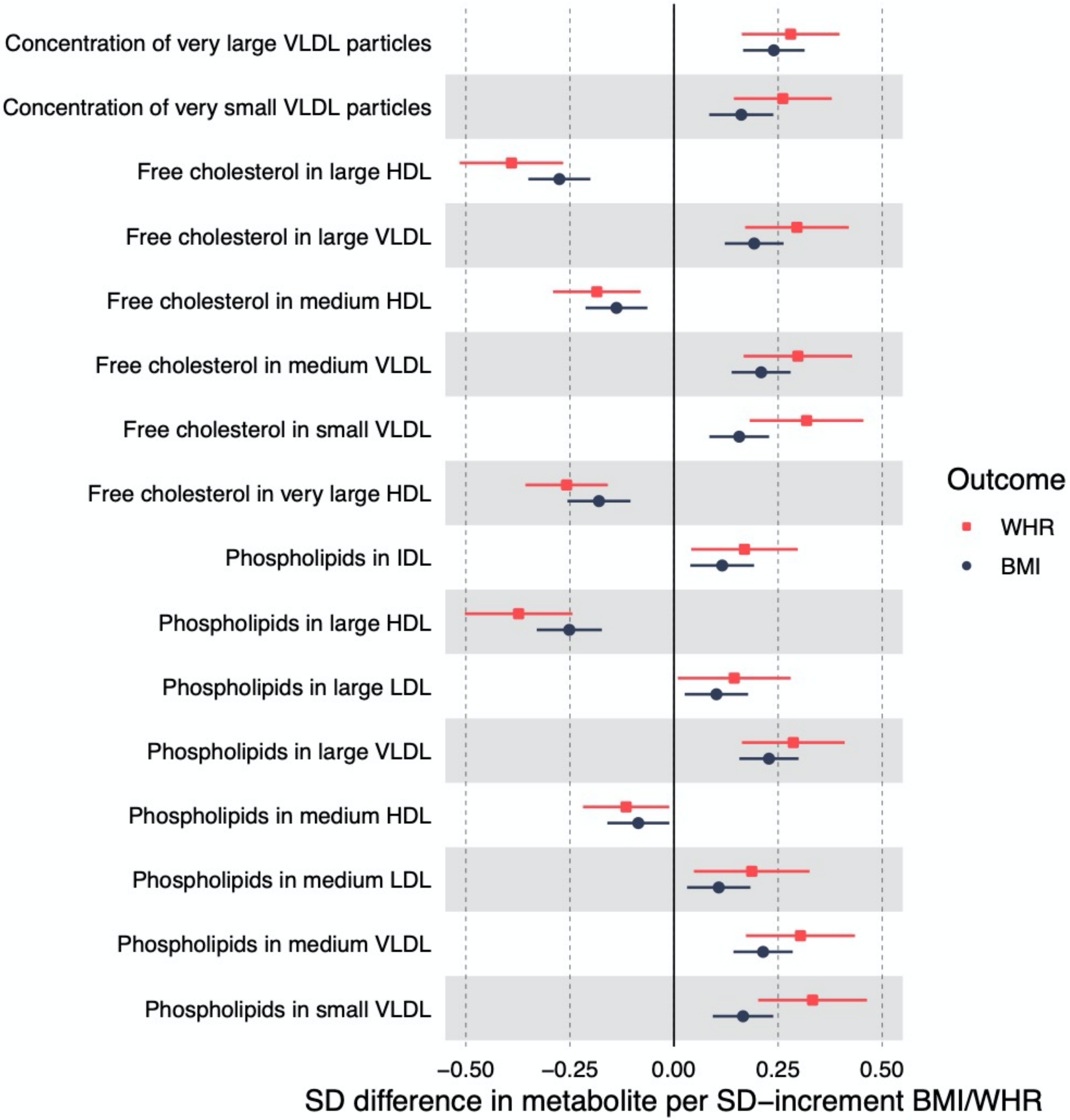

Figure S44. Effects of BMI and WHR on circulating metabolite levels (NMR-detected metabolites, 2 of 5), based on two-sample MR (IVW models) in summary GWAS consortia data.

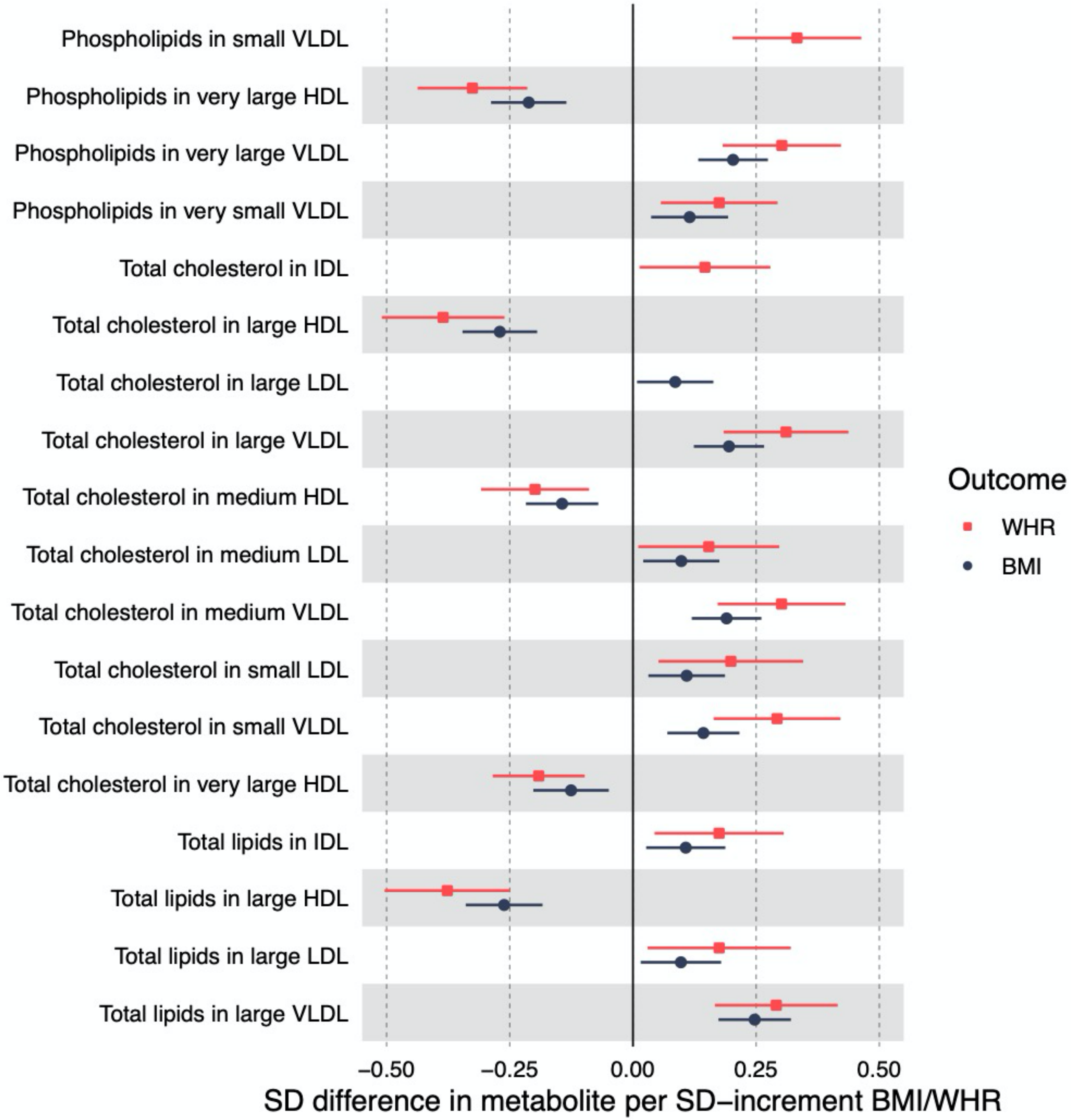

Figure S45. Effects of BMI and WHR on circulating metabolite levels (NMR-detected metabolites, 3 of 5), based on two-sample MR (IVW models) in summary GWAS consortia data.

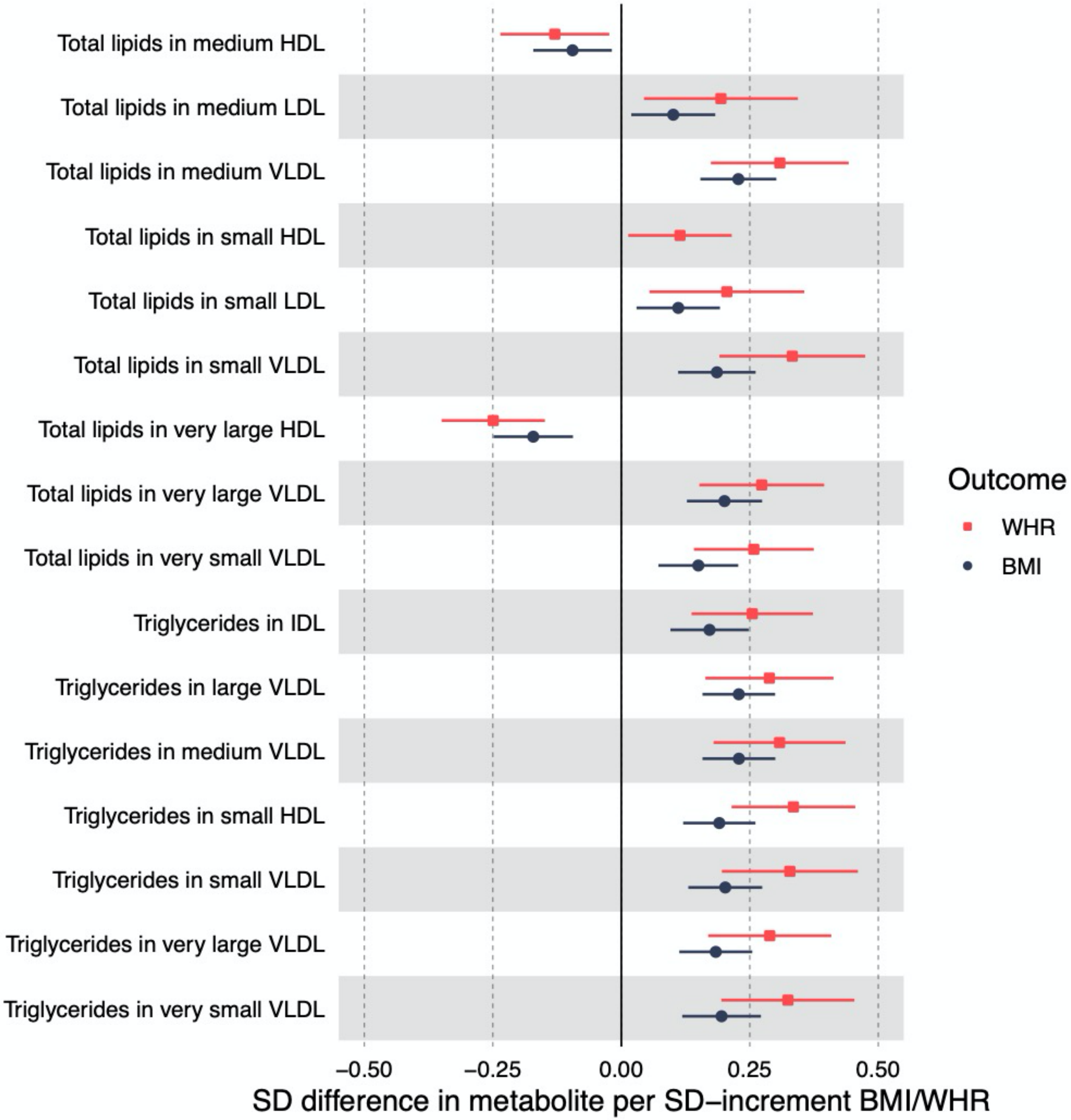

Figure S46. Effects of BMI and WHR on circulating metabolite levels (NMR-detected metabolites, 4 of 5), based on two-sample MR (IVW models) in summary GWAS consortia data.

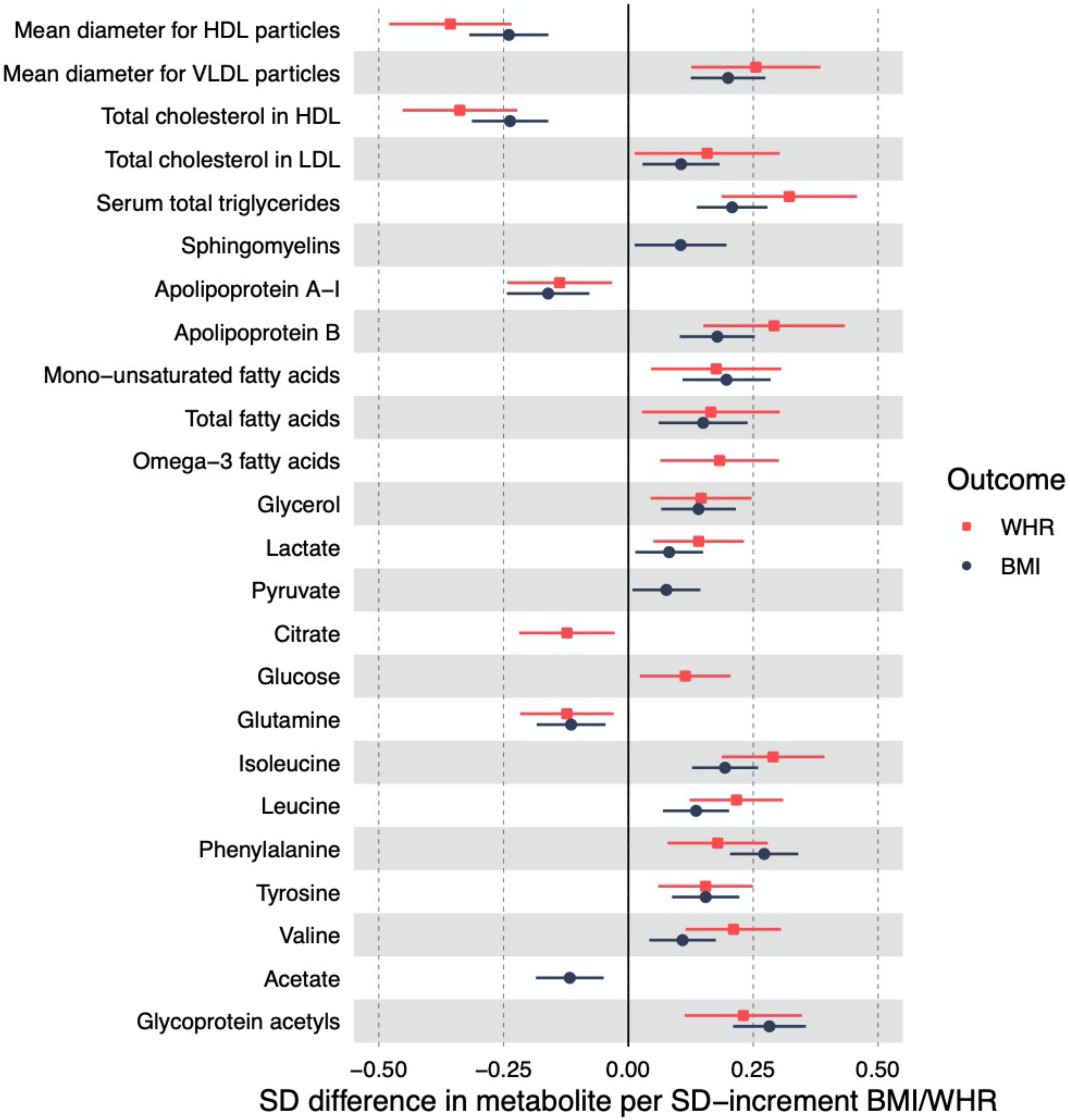

Figure S47. Effects of BMI and WHR on circulating metabolite levels (NMR-detected metabolites, 5 of 5), based on two-sample MR (IVW models) in summary GWAS consortia data.

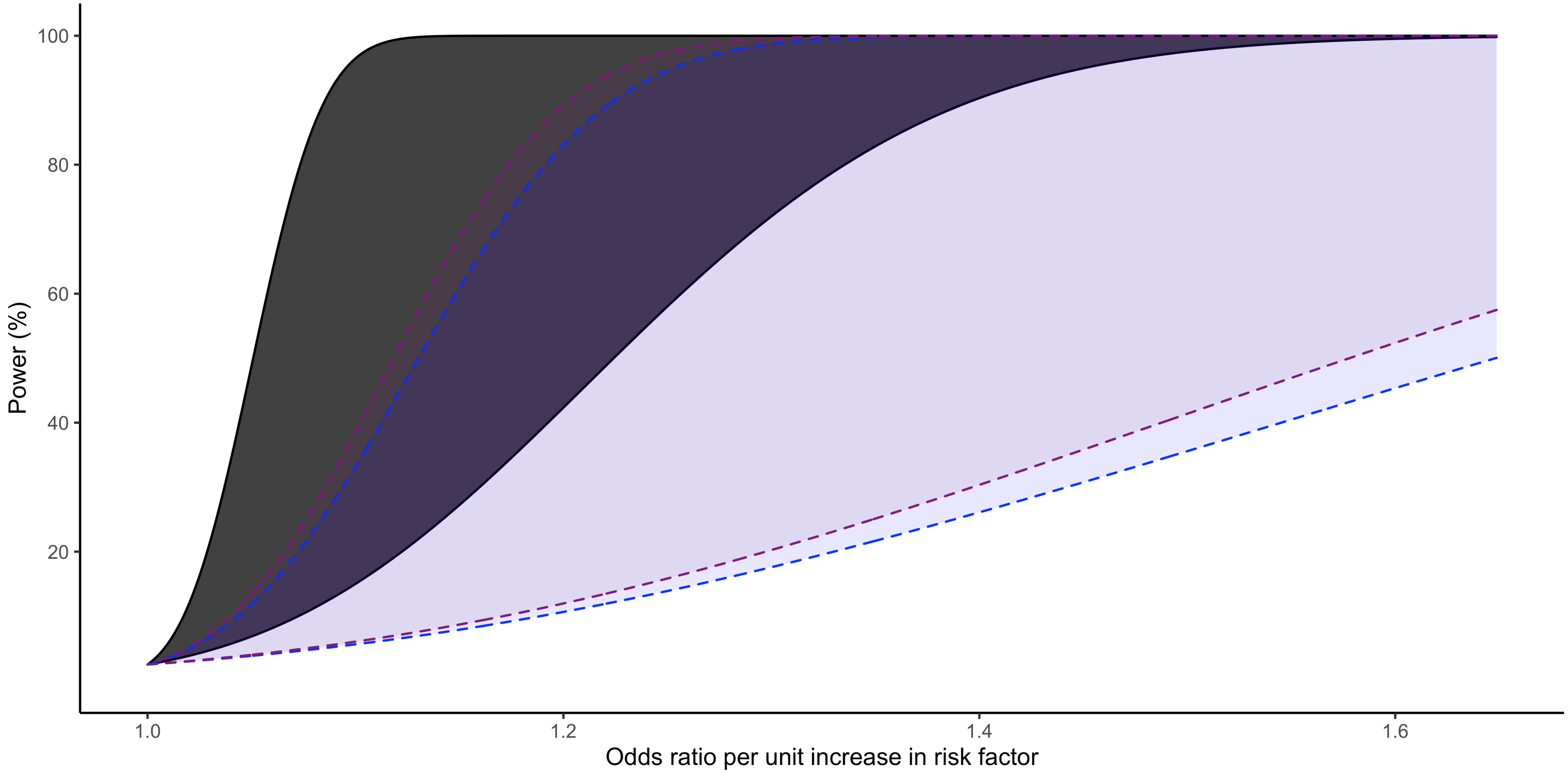

Figure S48. Power curves for MR analyses, based on samples sizes for colorectal cancer in the present study (black), Thrift et al., 2015 (blue) and Jarvis et al., 2016 (purple). Upper and lower power curves describe genetic instruments explaining 5% and 0.3% of variance respectively for each study
